# How Does the Ni–Ga Alloy Structure Tune Methanol Productivity and Selectivity?

**DOI:** 10.1021/acscatal.5c02008

**Published:** 2025-08-01

**Authors:** Nora K. Zimmerli, Andrés F. Usuga, Stefano Checchia, Aleix Comas-Vives, Christoph R. Müller, Paula M. Abdala

**Affiliations:** † Department of Mechanical and Process Engineering, 27219 ETH Zürich, Leonhardstrasse 21, 8092 Zurich, Switzerland; ‡ Departament de Química, 16719 Universitat Autònoma de Barcelona, 08193 Cerdanyola del Vallès, Catalonia, Spain; § Institute of Materials Chemistry, TU Wien, Getreidemarkt 9/165, 1060 Vienna, Austria; ∥ ESRF − The European Synchrotron, 71 Avenue des Martyrs, 38000 Grenoble, France

**Keywords:** Bimetallic catalysts, CO_2_ hydrogenation, nickel, gallium, operando, X-ray absorption spectroscopy, X-ray total scattering, pair distribution function

## Abstract

In this work, we assess how the structure of SiO_2_-supported, Ni–Ga alloys determines their activity and selectivity for the hydrogenation of CO_2_ to methanol. Using a hydrothermal deposition-precipitation approach followed by activation at 700 °C in H_2_, we synthesize catalysts containing α-Ni, α-Ni_9_Ga, α’-Ni_3_Ga, or δ-Ni_5_Ga_3_ phases supported on amorphous SiO_2_. Operando X-ray pair distribution function analysis and X-ray absorption spectroscopy confirm unequivocally the structure of all phases and their stability under reaction conditions; additionally, all catalysts contain GaO_*x*_ species in varying amounts. We observe that the catalysts α’-Ni_3_Ga/SiO_2_ and δ-Ni_5_Ga_3_/SiO_2_ exhibit high methanol formation rates (∼0.8 mmol_MeOH_ mol_Ni_^–1^ s^–1^), which are 27 times greater than those of α-Ni_9_Ga/SiO_2_ and α-Ni/SiO_2_. Notably, α’-Ni_3_Ga/SiO_2_ shows the highest selectivity for methanol at 71%, compared to 55% for δ-Ni_5_Ga_3_/SiO_2_ and 11% for α-Ni_9_Ga/SiO_2_, which challenges the conventional view of α’-Ni_3_Ga being a poor catalyst for methanol synthesis. To explain the high methanol selectivity and productivity of α’-Ni_3_Ga/SiO_2_ compared to the other alloy phases, DFT calculations were performed. It was found that the Ni-rich step sites in α’-Ni_3_Ga effectively stabilize key reaction intermediates (HCOO* and CH_3_O*) for the formation of methanol. However, such Ni-rich step sites in α’-Ni_3_Ga also favor CO* dissociation, which could facilitate methane formation, yet the presence of GaO_*x*_ decreases the stability of CO* on α’-Ni_3_Ga, explaining ultimately the promotion of HCOO* formation. This study highlights the importance of Ga species (both metallic and oxidic) in modulating the electronic properties of heterogeneous catalysts, providing a versatile toolbox to stabilize key reaction intermediates, leading ultimately to high product selectivity.

## Graphical abstract

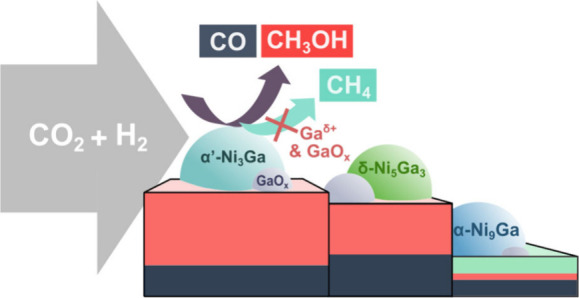

## Introduction

A strategy to reduce anthropogenic CO_2_ emissions is the direct hydrogenation of CO_2_ to methanol (*CO*_2_ + 3*H*_2_ ↔ *CH*_3_ *OH* + *H*_2_ *O*, Δ*H*_298*K*_ = −49.5*kJmol* ^–1^). This process is particularly promising due to the high versatility of methanol as both a platform chemical and an energy carrier.^1^ Methanol is produced industrially via the hydrogenation of a mixture of mainly CO with a small amount of CO_2_ over a Cu-ZnO-Al_2_O_3_ (CZA) catalyst. However, when the CZA catalyst is used with a feed containing a mixture of only CO_2_ and H_2_, it exhibits a low methanol formation rate and selectivity, as well as a rapid deactivation due to a reduction of the Cu surface area through sintering.^2,3^ Consequently, efforts have focused on developing catalysts that are active, selective, and stable for the direct hydrogenation of CO_2_ to methanol.^4–7^ These efforts can be categorized as (i) The modification of Cu-based catalysts with promoters (e.g., K, Ce, Zr, Ga, Ti, Ni);^5^ and (ii) the exploration of alternative catalyst families, including, different transition metals in combination with metal oxides e.g., Pd/ZnO, metal oxides such as In_2_O_3_, Zn-ZrO_2_, or intermetallic compounds (IMC), e.g., Pd–Ga, or Ni–Ga based.^1,8^

Among the IMCs, Ni–Ga intermetallics have emerged as promising candidates for this reaction.^9^ However, the structure–activity relationships in Ni–Ga systems remain a topic of debate. For instance, DFT studies have predicted α’-Ni_3_Ga and δ-Ni_5_Ga_3_ IMC to have a high catalytic activity. However, CO_2_ hydrogenation experiments (CO_2_:H_2_ = 1:3, 1–20 bar and 160–300 °C) using a series of SiO_2_-supported Ni–Ga IMC catalysts (viz. α’-Ni_3_Ga/SiO_2_, δ-Ni_5_Ga_3_/SiO_2_, and β-NiGa/SiO_2_) revealed δ-Ni_5_Ga_3_/SiO_2_ as the catalyst with the highest methanol formation rate and selectivity. In contrast, α’-Ni_3_Ga/SiO_2_ favored side reactions, such as CO formation via the reverse water–gas shift reaction (*CO*_2_ + *H*_2_ ↔ *CO* + *H*_2_ *O*, Δ*H*_298*K*_ = 41.2 *kJmol* ^–1^) and CH_4_ formation via the Sabatier reaction (*CO*_2_ + 4*H*_2_ ↔ *CH*_4_ + 2*H*_2_ *O*, Δ*H*_298*K*_ = −165 *kJmol* ^–1^).^9–12^ It was hypothesized that the poor performance of α’-Ni_3_Ga/SiO_2_ was due to the rapid deactivation of Ni surface sites which were poisoned by adsorbed CO and carbon deposition.^9^ A further study exploring the Ni–Ga system, also reported a SiO_2_-supported δ-Ni_5_Ga_3_ phase to be the phase with the highest methanol formation rate, yet it was also observed that this catalyst contained a mixture of δ-Ni_5_Ga_3_ and α’-Ni_3_Ga phases, hence making it difficult to distinguish between the activities of the individual phases.^11^ Indeed, additional studies have shown that although δ-Ni_5_Ga_3_ might have been present in the as-synthesized material, an α’-Ni_3_Ga phase was observed after reaction, suggesting a phase transformation under reaction conditions.^10–13^

Another area of controversy in the Ni–Ga system concerns the presence and role of gallium oxide (GaO_*x*_) species under CO_2_ hydrogenation conditions, as GaO_*x*_ has been reported to coexist with Ni–Ga alloys under reaction conditions.^10,11,13–20^ While some studies have suggested that amorphous GaO_*x*_ on the surface of metallic nanoparticles enhances methanol synthesis by promoting CO_2_ activation,^14,21^ other studies have shown that, if an air-exposed δ-Ni_5_Ga_3_ catalyst is not activated at a sufficiently high temperature in H_2_ prior to CO_2_ hydrogenation, the high amount of GaO_*x*_ in the catalyst reduces its methanol activity.^14^ In general, there is a considerable lack of mechanistic understanding of the role of GaO_*x*_ in Ni–Ga alloy catalysts for methanol formation.

Thus, although Ni–Ga-based catalysts have demonstrated a promising performance for the selective hydrogenation of CO_2_ to methanol, there is uncertainty concerning (i) the catalytically most active and selective structural motif, (ii) the phase stability under reaction conditions and (iii) the role of GaO_*x*_ species on methanol formation rate and selectivity. To address these knowledge gaps, we prepared a series of silica-supported Ni–Ga-based catalysts, viz. δ-Ni_5_Ga_3_/SiO_2_, α’-Ni_3_Ga/SiO_2_, and α-Ni_9_Ga/SiO_2_ and characterized their geometric and electronic structures during H_2_ activation and CO_2_ hydrogenation conditions using operando XRD, differential pair distribution function analysis (d-PDF), and XAS. These studies confirmed the phase purity and stability of the SiO_2_-supported δ-Ni_5_Ga_3_, α’-Ni_3_Ga, and α-Ni_9_Ga under CO_2_ hydrogenation conditions. Additionally, all catalysts were found to contain GaO_*x*_ species. With regard to their methanol formation activity, α’-Ni_3_Ga/SiO_2_ and δ-Ni_5_Ga_3_/SiO_2_ showed comparable methanol formation rates, with α’-Ni_3_Ga/SiO_2_ exceeding the methanol selectivity of δ-Ni_5_Ga_3_/SiO_2_, while α-Ni_9_Ga/SiO_2_ and α-Ni/SiO_2_ yielded considerably lower methanol formation rates and comparatively high methane formation rates. DFT calculations allowed us to rationalize our observations, indicating that alloying Ni with Ga limits CO* dissociation that in turn reduces methane formation. In addition, the presence of GaO_*x*_ on α’-Ni_3_Ga decreases the stability of CO* compared to the bare alloy surface, hence favoring the hydrogenation of CO_2_ to formate (a key methanol formation intermediate) rather than CO_2_ dissociation.

## Experimental Section

### Catalyst Synthesis

The catalysts were synthesized via a hydrothermal deposition-precipitation approach adapted from Bian et al.^22^ whereby the nominal metal loading of Ni+Ga on SiO_2_ was fixed to 5 wt %. For a yield of ca. 1 g of catalyst precursor, appropriate amounts (see Table S1) of the metal nitrate precursors [Ni­(NO_3_)_2_·6H_2_O and Ga­(NO_3_)_3_·xH_2_O with x = 5.22 determined by thermogravimetric analysis, both Acros Organics] and 2 g of urea (Thermo Scientific Chemicals) were weighed into a 50 mL beaker. Next, 10–15 mL of deionized water were added to the beaker and stirred until the metal precursors and urea were completely dissolved. Subsequently, 2 g of Ludox TM-50 colloidal SiO_2_ (Sigma-Aldrich, 50 wt % suspension in H_2_O) was added dropwise to the solution while stirring at 300 rpm. Once all of the colloidal SiO_2_ was added, the solution was filled into a 30 mL glass inset of a 50 mL PTFE lined autoclave (Parr Instrument Company). The glass inset was filled up with deionized water until ca. 3/4 full, and the mixture was stirred for another 1–2 min. The glass inset was securely placed inside the autoclave, which was tightly sealed and placed in an oven that was held at 100 °C for 24 h. The resulting precipitate was collected by centrifugation for 5 min at 4000 rpm. The precipitate was washed three times by mixing with deionized H_2_O, centrifugation, and decanting of the supernatant. The precipitate was dried in an oven held at 100 °C for 12 h and ground to a fine powder using a pestle and a mortar. To obtain the activated catalysts, the catalyst precursors were placed into a quartz tube reactor and treated in 10% H_2_ in N_2_ (50 mL/min) at 700 °C (10 °C/min) at atmospheric pressure for 4 h. The elemental composition of the activated catalysts was determined via inductively coupled plasma optical emission spectroscopy (ICP-OES) and is reported in Table S2. The activated catalysts are denoted by specifying the nanocrystalline phase in the catalyst, as detected by X-ray total scattering experiments viz. α-Ni/SiO_2_, α-Ni_9_Ga/SiO_2_, α’-Ni_3_Ga/SiO_2_, and δ-Ni_5_Ga_3_/SiO_2_. An additional GaO_*x*_^/^SiO_2_ material was prepared as a reference for X-ray absorption spectroscopy, following the same procedure as for Ni_*x*_Ga_*y*_/SiO_2_ (omitting the Ni precursor) and treated under H_2_ at 700 °C.

### Characterization

#### Electron Microscopy

High-angle annular dark-field scanning transmission electron microscopy (HAADF-STEM) images were recorded on a FEI Talos F200X operated at 200 keV (Figures S2–S4) and a JEOL JEM-ARM300F GRAND ARM operated at 300 kV, both equipped with EDX detectors. To this end, the powdered samples were mixed with a Lacey-C 400 mesh Cu grid under ambient air conditions. The size of the nanoparticles was determined using the software ImageJ (version 1.54b).^23^ The obtained particle size distribution was fitted by a log-normal distribution.

#### X-ray Total Scattering and PDF Analysis

X-ray total scattering experiments were conducted at ID15A of the European Synchrotron Radiation Facility. Data of α-Ni_9_Ga/SiO_2_, α’-Ni_3_Ga/SiO_2_, and δ-Ni_5_Ga_3_/SiO_2_ were collected continuously at an incident X-ray energy of 68.5 keV (0.181 Å, square beam size of 100× 100 μm^2^) up to *Q*_max_ = 26 Å^–1^ at a rate of 1 measurement/4.65 min, using a Pilatus3 CdTe 2 M area detector.^24^ Total scattering data of the pristine silica support were acquired under in situ activation and reaction conditions and used as background to calculate the d-PDF data. In addition, total scattering data of a CeO_2_ NIST reference were obtained to determine the experimental resolution parameters Q_damp_ and Q_broad_.

The in situ/operando setup at the beamline consisted of a custom-made capillary cell reactor which was connected to a manifold of mass flow controllers (Bronkhorst EL-FLOW series, max. 30 mL/min, *P*_max_ = 35 bar) and a backpressure regulator (Bronkhorst, EL-PRESS series, *P*_max_ = 35 bar). The catalyst bed consisted of ca. 2–3 mg of catalyst that was placed between two quartz wool plugs inside a quartz capillary (Hilgenberg, 1 mm OD, 0.02 mm wall thickness). The capillary was heated via a hot air blower from below. Prior to the experiments, the temperature was carefully calibrated in the range 50 – 800 °C by placing a thermocouple into a capillary filled with quartz wool. The off-gas was analyzed via a compact gas chromatograph (Global Analyzer Solutions, Compact GC^4.0^) equipped with TCD and FID detectors and a sampling rate of ca. 1 scan/7 min. The limits of detection of the GC were <10 ppm for methanol and CH_4_ and <500 ppm for CO. A typical in situ/operando experiment consisted of an activation step (heating up from room temperature to 700 °C in H_2_ at 1 bar), followed by pressurization and CO_2_ hydrogenation, as detailed in Figure S5.

To obtain the d-PDF from the total scattering patterns of Ni_*x*_Ga_*y*_/SiO_2_, the SiO_2_ background was subtracted, followed by data normalization and Fourier transform, performed using the PDFgetX3 software (v 2.2.1).^25,26^ The total scattering data were processed within the range *Q*_min_ = 1 Å^–1^ and *Q*_max_ = 23 Å^–1^ using r_poly_ = 1.1, which is approximately the r-limit of the maximum frequency in the F­(Q) correction polynomial.

Modeling of the d-PDF was performed in PDFGui (v 2.0.3),^27,28^ using fcc-Ni_9_Ga (ICSD #8688, partial occupancy of 0.9/0.1 for Ni/Ga on the Wyckoff site 4a, x = y=z = 0),^29^ α’-Ni_3_Ga (ICSD #103856),^30^ and δ-Ni_5_Ga_3_ (ICSD #103861)^31^ as the initial model structures for α -Ni_9_Ga/SiO_2_, α‘-Ni_3_Ga/SiO_2_, and δ-Ni_5_Ga_3_/SiO_2_, respectively. The d-PDFs were fitted between 1.7 – 25 Å. The fitted crystal structure parameters are reported in Tables S3–S5. The Q_damp_ and Q_broad_ parameters were set to the values obtained from fitting the CeO_2_ reference (i.e., Q_damp_= 0.0129 Å^–1^ and Q_broad_= 0.0168 Å^–1^). Modeling the d-PDF of α‘-Ni_3_Ga/SiO_2_ using a fcc-Ni_3_Ga random alloy (*Fm3̅m* space group, partial occupancy of 0.75/0.25 for Ni/Ga on the Wyckoff site 4a, x = y=z = 0) was also probed, which resulted in fitted parameters equal (within the error) to the ones obtained when using a α’-Ni_3_Ga model structure.

#### X-ray Absorption Spectroscopy

XAS experiments were conducted at BM31 of the European Synchrotron Radiation Facility using the same capillary reactor setup that was used for the X-ray total scattering experiments. Ni and Ga K-edge XAS scans of α-Ni/SiO_2_, α-Ni_9_Ga/SiO_2_, α’-Ni_3_Ga/SiO_2_, and δ-Ni_5_Ga_3_/SiO_2_ were collected consecutively (details in section 4 of the Supporting Information), covering the energy ranges specified below and using an X-ray beam with a width of 2 mm and a height of 0.5 mm. The data collected during the in situ activation step (heating up from room temperature to 700 °C in H_2_ at 1 bar) can be found in Figures S17–S19 of the Supporting Information. The XANES data obtained during the activation at the Ni and Ga K-edges indicate that both Ni and Ga were initially partially oxidized due to exposure to air. During the activation treatment, the oxidation states of both Ga and Ni species evolve, showing reduction. These results highlight the importance of in situ investigations for understanding structural performance relationships and their conclusions. A typical in situ/operando XAS experiment is shown in Figure S5. To probe the structural dynamics of α’-Ni_3_Ga/SiO_2_ under different gas atmospheres, α’-Ni_3_Ga/SiO_2_ was exposed to the following gas sequence: H_2_–CO_2_–H_2_ for, respectively, 90 min – 180 min – 90 min (20 bar, 12 mL/min, 230 °C) after having been kept under reaction conditions (20 bar CO_2_:H_2_:N_2_ = 1:3:1, 5 mL/min, 230 °C) for ca. 5 h.

The energy scale of the Ni and Ga K-edge spectra was calibrated by setting the absorption edge positions [maximum of the first derivative of μ­(E)] of the reference samples Ni-foil and Zn foil to the established values of 8333.0 and 9659.0 eV, respectively. Linear combination fittings of the normalized XANES data [μ­(E)] were performed using the Athena/Demeter v 0.9.26 software^32^ between −20 and +50 eV around the edge position (E_0_), constraining the LCF weights to values between 0 and 1 and their sum to 1. Extended X-ray absorption fine structure (EXAFS) fittings were performed using the Artemis/Demeter v 0.9.26 software.^32^ Additional information about the EXAFS fittings can be found in the Supporting Information section 4.

#### Infrared Spectroscopy (DRIFTS)

Operando DRIFTS experiments were performed using a Nicolet 6700 FT-IR equipped with a Harrick Praying Mantis DRIFTS accessory and a high-temperature reaction chamber. Data were collected from 650 cm^–1^ – 4000 cm^–1^ with a spectral resolution of 4 cm^–1^ using a mercury cadmium telluride detector cooled with liquid nitrogen. In a typical experiment, ca. 30 mg of powder were placed onto a piece of quartz wool in the sample cup of the reaction cell. Next, the catalyst was activated in situ in 15% H_2_/N_2_ (20 mL/min) at 590 °C (10 °C/min) for 1 h and subsequently cooled down to 230 °C. Next, the cell was pressurized to 20 bar in N_2_ (20 mL/min). Once the pressure had stabilized, a measurement was collected which served as background for all the subsequent measurements under reaction conditions. Next, the atmosphere was switched to CO_2_:H_2_:N_2_ = 1:3:1 (20 bar, 20 mL/min) and measurements were continuously collected every 1.3 min for ca. 2 h. The compact GC that was used for the operando XAS/PDF experiments was also used for the operando DRIFTS experiments.

#### CO_2_ Hydrogenation Tests in a Laboratory-Scale Reactor

For the catalytic tests, 100 mg of the as-prepared powder was transferred into a Hastelloy C276 reactor tube (internal diameter 9.1 mm). The powder was placed, between two pieces of quartz wool (Acros Organics, 9–30 μm) onto a frit that was located in the center of the reactor tube (Hastelloy C276, pore size 2 μm). The reactor was mounted into a Microactivity-Efficient flow reactor system (PID Eng & Tech). The CO_2_ hydrogenation tests were performed as follows: First, the catalyst was heated to 700 °C (10 °C/min) in H_2_ at 1 bar (50 mL/min) and held at 700 °C for 1 h. This step is referred to as in situ activation. Subsequently, the catalyst was cooled down to the reaction temperature of 230 °C (in H_2_ at 1 bar, 50 mL/min), followed by a switch of the gas flow to 80 mL/min N_2_ and increasing the pressure to 25 bar. After stabilization of the pressure and temperature, the gas feed was switched to 25 bar of CO_2_:N_2_:H_2_ = 1:1:3 (100 mL/min, corresponding to a GHSV of 60 L gcat^–1^ h^–1^) and the off-gas was continuously analyzed by a gas chromatograph (PerkinElmer Clarus 580 equipped with FID and TCD detectors, 1 injection/30 min). The equations used to calculate the catalytic performance parameters (product formation rates, product selectivities, CO_2_ conversion) can be found in the Supporting Information section 5. To determine the apparent activation energies for methanol, CO, and methane formation, an additional series of catalytic tests was conducted using 50 mg of catalyst with a total gas flow rate of 50 mL min^–1^, corresponding to a GHSV of 60 L gcat^–1^ h^–1^. The catalysts were activated following the same in situ protocol described above. Reactions were carried out at 25 bar using a CO_2_:H_2_:N_2_ feed ratio of 1:3:1, while systematically varying the reaction temperature across 160, 190, 210, 230, and 260 °C.

#### Density Functional Theory Calculations

The Ni–Ga systems were modeled as periodic slabs. We selected both flat and stepped surfaces of the α-Ni, α’-Ni_3_Ga, and δ-Ni_5_Ga_3_ crystal structures (Table S11). Specifically, we considered the flat α-Ni(111), α’-Ni_3_Ga­(111), and δ-Ni_5_Ga_3_(221) surfaces, as well as the stepped α-Ni(211), α’-Ni_3_Ga­(211), and δ-Ni_5_Ga_3_(211) surfaces. For α’-Ni_3_Ga­(211) and δ-Ni_5_Ga_3_(211), two stepped terminations were evaluated: one with a higher fraction of Ni and a second one with a higher fraction of Ga. We determined the binding energies of key intermediate species in CO_2_ hydrogenation, viz. C*, O*, CO*, HCOO*, CH_3_O*, and CO_2_*. Assessing these binding energies offers insight into which reaction pathway - reverse water–gas shift, methanation, or methanol synthesis - is favored on the various Ni–Ga surfaces. Two additional models were constructed to assess the role of the GaO_*x*_/Ni–Ga interface on the binding energy of the selected intermediates. These models consisted of a Ga_2_O­(OH)_2_ cluster positioned on either a α’-Ni_3_Ga (111) or a α’-Ni_3_Ga­(211) surface since α’-Ni_3_Ga­(211)_Ni‑step_ is the most stable stepped surface among the α’-Ni_3_Ga systems (see Table S12). The two OH groups in Ga_2_O­(OH)_2_ were included to stabilize the GaO_*x*_ cluster. We obtained the binding energy of the selected intermediates referenced against a common reference, i.e, the sum of the electronic energies of the reactants *C* *O*_2_(*g*) + 3*H*_2_(*g*). All elementary reactions considered are described in section 7 of the Supporting Information. For the GaO_*x*_/α’-Ni_3_Ga system, we calculated the adsorption energies of key intermediates interacting either simultaneously with the GaO_*x*_ cluster and the α’-Ni_3_Ga surface, or exclusively with one of the two Ga centers of the Ga_2_O­(OH)_2_ cluster. Additionally, two adsorption modes for O* were considered for the interface system: O* interacting simultaneously with the Ga_2_O­(OH)_2_ cluster and the Ni–Ga surface, referred to as O*_inter_, and O* interacting only with the Ni–Ga surface, i.e., not interacting with the GaO_*x*_ cluster, referred to as O*_Ni–Ga_. Periodic DFT calculations were carried out as implemented in the Vienna Ab initio Simulation Package (VASP).^33–35^ The effect of the core electrons on the valence density was described using the projector augmented wave method.^36,37^ The simulations were performed using the BEEF-vdW^38^ exchange-correlation functional without spin-polarization. A plane wave basis set with a kinetic energy cutoff of 500 eV was selected to expand the valence electron density. Structure relaxation was performed until the forces acting on each atom were smaller than 0.01 eV/Å. A vacuum layer of 15 Å was chosen to prevent perpendicular interactions between periodic images. Further information such as calculated lattice parameters, supercell size, and *k*-mesh grid, are provided in Tables S10 and S11 of the Supporting Information.

We employed the climbing-image nudge elastic band (CI-NEB) method to locate the saddle point on the potential energy surface; i.e. transition-state between the initial and final configuration.^39^ These calculations were performed using 8 intermediate images. Once the image with the highest energy was identified, the dimer method was employed to refine the transition state, continuing until the rotational force converged to below 0.01 eV/Å. After evaluating the stability trends of the additional elementary steps (see section 7), we calculated the transition states leading to the formation of HCOO* and H_2_COOH* on the α’-Ni_3_Ga­(111) and α’-Ni_3_Ga­(211)_Ni‑step_ surfaces, as they are considered to be most representative of the α’-Ni_3_Ga system. These transition states were selected because they correspond to the states with the highest energy barriers in the formate-based methanol synthesis pathway as reported by Studt et al.^9^ The barrier energies are summarized in Table S16.

## Results and Discussion

### Structure of the Activated Catalysts

The following series of catalysts, i.e. α-Ni/SiO_2_, α-Ni_9_Ga/SiO_2_, α’-Ni_3_Ga/SiO_2,_ and δ-Ni_5_Ga_3_/SiO_2_ was synthesized via a hydrothermal deposition-precipitation approach followed by a reductive treatment at 700 °C in 10% H_2_/N_2_ for 4 h to activate the catalysts (see ICP composition in Table S2 and TEM images in Figures S2–S4).^22^ HAADF-STEM images of the materials after activation (and after their exposure to ambient air) visualized the formation of nanoparticles with an average diameter of 4.2 ± 0.7 nm (α-Ni/SiO_2_), 4.9 ± 1.3 nm (α-Ni_9_Ga/SiO_2_), 7.7 ± 2.2 nm (α’-Ni_3_Ga/SiO_2_), and 6.9 ± 0.2 nm (δ-Ni_5_Ga_3_/SiO_2_) (Figure S3, Table S2). Energy-dispersive X-ray (EDX) spectroscopy maps of the activated catalysts indicated that both Ni and Ga were present in the nanoparticles (Figure S4). In the following, we applied in situ X-ray total scattering experiments and d-PDF analysis to probe the atomic-scale structure of the nanoparticles obtained after their activation (treated at 700 °C with 10 °C/min in 1 bar H_2_). The contribution of the SiO_2_ support was subtracted from the total scattering signal.^40^ The SiO_2_-subtracted X-ray scattering patterns of α-Ni_9_Ga/SiO_2_, α’-Ni_3_Ga/SiO_2_, and δ-Ni_5_Ga_3_/SiO_2_ are shown in Figure 1. The Bragg reflections observed in α-Ni_9_Ga/SiO_2_ (Figure 1A) and α’-Ni_3_Ga/SiO_2_ (Figure 1C) correspond to, respectively, a fcc alloy α-Ni­(Ga) and an intermetallic α’-Ni_3_Ga structure. The unit cells representing the α-Ni­(Ga) and α’-Ni_3_Ga structures are given as insets in Figures 1A and 1C, respectively. Although the structures of α-Ni­(Ga) and α’-Ni_3_Ga are similar, their diffraction patterns exhibit subtle yet distinct differences. The key distinguishing feature in the diffraction patterns is the presence of superstructure reflections i.e. (110), (210) and (211) reflections in α’-Ni_3_Ga which are forbidden in a α-Ni­(Ga) random alloy.^41^ The intensity of these superstructure reflections in α’-Ni_3_Ga is relatively weak, ca. 100 times less intense than the most prominent α’-Ni_3_Ga­(111) reflection^42^ and only observable due to the high signal-to-noise ratio provided by the beamline. In this context, it is important to emphasize that in previous works, the identification of the α’-Ni_3_Ga phase largely relied on a match between the most prominent peak positions in the experimental XRD data and an α’-Ni_3_Ga reference, yet such peaks are shared between a fcc α-Ni­(Ga) alloy and α’-Ni_3_Ga and can hence not serve as a distinguishing feature between these two phases. The identification of supercell reflections, i.e. the only distinguishing feature of the α’-Ni_3_Ga phase, was not reported in these works.^9–13^ Figure 1E shows the diffraction pattern of δ-Ni_5_Ga_3_/SiO_2_ that is very different from the pattern observed for α-Ni­(Ga) or α’-Ni_3_Ga and which matches very well the ICSD #103861 δ-Ni_5_Ga_3_ reference, thus confirming the successful synthesis of SiO_2_ supported nanocrystalline δ-Ni_5_Ga_3_ particles. Next, we performed d-PDF fittings to quantitatively describe the phases formed. Fitting of the d-PDF of α-Ni_9_Ga/SiO_2_ (Figure 1B) and α’-Ni_3_Ga/SiO_2_ (Figure 1D) with the α-Ni_9_Ga and α’-Ni_3_Ga model structures showed excellent agreement between the model crystal structures and the data measured (weighted agreement factors, R_w_ = 0.0729 and 0.0809, respectively). The fitted parameters are reported in Tables S3–S5. The cubic lattice parameter of α’-Ni_3_Ga/SiO_2_ [3.6006(9) Å] was larger than that of α-Ni_9_Ga/SiO_2_ [3.5445(8) Å], in line with the presence of a more Ni-rich alloy in α-Ni_9_Ga/SiO_2_ and with previous literature.^43,44^ The fitted orthorhombic lattice parameters of δ-Ni_5_Ga_3_ in δ-Ni_5_Ga_3_/SiO_2_ were a = 7.42(2) Å, b = 6.80(1) Å, and c = 3.768(7) Å, which are in close agreement with previously reported values for this phase.^45^ Notably, the coherent diameters of the nanoparticles, as extracted from the fittings of the d-PDF data, were very similar between the three materials and ranged between 6.2 and 6.5 nm. These results are comparable to particle sizes obtained by TEM measurements for δ-Ni_5_Ga_3_/SiO_2_ (6.9 ± 2.2 nm) and α’-Ni_3_Ga/SiO_2_ (7.7 ± 2.2 nm). However, TEM indicated a slightly smaller diameter for α-Ni_9_Ga/SiO_2_ (4.9 ± 1.3 nm). It is important to note that the PDF analysis yields an average coherence length of the nanoparticles representative of the entire sample volume illuminated by the X-ray beam, whereas the TEM-based particle size determination is derived from a significantly smaller sampled volume and is based on measurements of only ∼ 200 individual particles in the present study.

**Figure 1:**
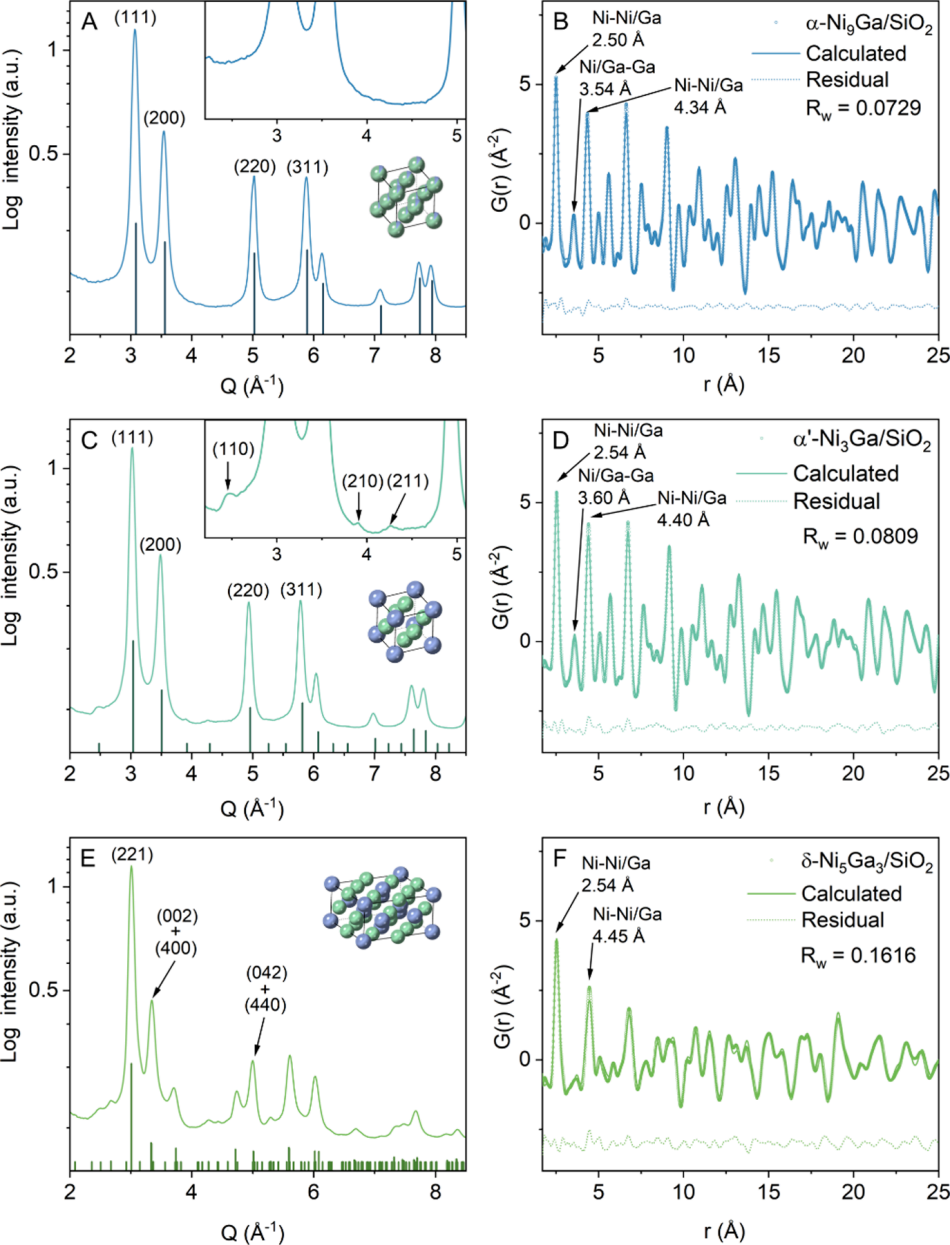
SiO_2_-subtracted total scattering and corresponding d-PDF data of (A, B) α-Ni_9_Ga/SiO_2_, (C, D) α’-Ni_3_Ga/SiO_2_, and (E, F) δ-Ni_5_Ga_3_/SiO_2_. The data was collected at 230 °C in 1 bar H_2_, following the in situ activation of the catalysts (700 °C, H_2_). The X-ray scattering patterns are plotted together with simulated patterns for α-Ni_9_Ga (ICSD #8688), α’-Ni_3_Ga (ICSD #103856), and δ-Ni_5_Ga_3_ (ICSD #103861). The d-PDF were fitted to the α-Ni_9_Ga, α’-Ni_3_Ga, and δ-Ni_5_Ga_3_ model structures. The weighted R factor (R_w_) is an indicator of the agreement of the fitting with the experimental data. The inset in (C) shows some of the supercell reflections due to the α’-Ni_3_Ga phase in α’-Ni_3_Ga/SiO_2_.

To obtain further details of the electronic and local geometric structure of the activated catalysts in situ XAS at the Ni and Ga K-edges was performed during activation (Figure 2). The maximum of the first derivative of the Ni K-edge XANES data of α-Ni/SiO_2_, α-Ni_9_Ga/SiO_2_, α’-Ni_3_Ga/SiO_2_, and δ-Ni_5_Ga_3_/SiO_2_ was at ∼ 8333 eV (*a* in Figure 2A, Figure S13). This value agrees well with the absorption edge of metallic Ni,^32^ indicating that Ni was in its metallic state in all of the activated catalysts. In α-Ni/SiO_2_ and α-Ni_9_Ga/SiO_2_ the XANES features *c, d* and *e* at 8350, 8355, and 8359 eV, respectively, closely resemble those observed in the Ni foil reference (Figure 2A),^46^ while the XANES features of α’-Ni_3_Ga/SiO_2_, and δ-Ni_5_Ga_3_/SiO_2_ are markedly different from those of the Ni-foil and α-Ni/SiO_2_. Specifically, feature *b* in these catalysts is shifted to lower energies (Figure 2A, inset), indicative of an electron transfer from Ga to Ni, i.e. presence of Ni^δ‑^ (and Ga^δ+^) sites.^47,48^ This charge transfer is in line with our Bader charge analysis presented in section 7 of the Supporting Information, which revealed an increase in the transferred charge |δ| with increasing Ga content in the alloy (Table S14). Moreover, in α’-Ni_3_Ga/SiO_2_ and δ-Ni_5_Ga_3_/SiO_2_, instead of features *c* and *e*, a feature *d* appears at 8355 eV, which is linked to the formation of intermetallic structures in these materials.^49^ Turning to the Ga speciation in the activated catalysts, the Ga K-edge XANES data of α-Ni_9_Ga/SiO_2_, α’-Ni_3_Ga/SiO_2_, and δ-Ni_5_Ga_3_/SiO_2_ were compared with the following references: bulk α’-Ni_3_Ga,^21^ GaO_*x*_/SiO_2_, and bulk β-Ga_2_O_3_ (Figure 2B). The Ga K-edge absorption edge positions (*f* in Figure 2B) and the XANES feature *g* of α-Ni_9_Ga/SiO_2_, α’-Ni_3_Ga/SiO_2_, and δ-Ni_5_Ga_3_/SiO_2_ were close to that of the bulk α’-Ni_3_Ga reference. However, we observed a slight shift of feature *f* to higher energies with respect to α’-Ni_3_Ga for all catalysts, whereby the magnitude of the shift, ΔE, increases with increasing Ga content of the material (at μ_norm_ = 0.5, ΔE = 0.1, 0.4, and 0.8 eV for α-Ni_9_Ga/SiO_2_, α’-Ni_3_Ga/SiO_2_, and δ-Ni_5_Ga_3_/SiO_2_, respectively). Furthermore, the white line intensity, *g*, increased with the same trend with respect to the bulk α’-Ni_3_Ga reference, i.e. δ-Ni_5_Ga_3_/SiO_2_ > α’-Ni_3_Ga/SiO_2_ > α-Ni_9_Ga/SiO_2_. These observations indicate that in α-Ni_9_Ga/SiO_2_, α’-Ni_3_Ga/SiO_2_, and δ-Ni_5_Ga_3_/SiO_2_, Ga existed in both a metallic and oxidic state after activation, with the fraction of oxidic Ga species increasing with the overall Ga content in the material. Quantifying the fraction of oxidic and alloyed gallium (Ga) presents challenges due to the lack of representative references for different alloys and oxidic Ga species. Nonetheless, to perform a comparative analysis of the Ga oxidic species in these materials, we used linear combination fitting (LCF) analysis of the Ga K-edge XANES using GaO_*x*_/SiO_2_ and bulk α’-Ni_3_Ga as references as they yielded the best agreement between the experimental data and the fit when compared to other combinations of references, including Ga-foil, β-Ga_2_O_3_. The GaO_*x*_/SiO_2_ reference was synthesized using the same procedure as the Ni–Ga/SiO_2_ catalysts and treated under H_2_ at 700 °C and contained noncrystalline gallium species dispersed on SiO_2_, which includes mostly Ga^3+^ and possibly Ga^+^/Ga^3+^ hydride species.^21,50^ It is important to note that α’-Ni_3_Ga was the only available reference for a Ni–Ga alloy, and while we did not aim at precisely resolving the type of alloy using XANES (XRD was the most suitable technique for resolving the type of alloy), LCF allowed us to compare the fractions of alloyed Ga and GaO_*x*_ in the different catalysts. LCF analysis estimated that α-Ni_9_Ga/SiO_2_, α’-Ni_3_Ga/SiO_2_, and δ-Ni_5_Ga_3_/SiO_2_ contain, respectively, 20%, 34%, and 58 mol % GaO_*x*_ with respect to the total Ga content, while the remainder is Ga (Ga^δ+^) in a Ni–Ga alloy (see also Figure S11). However, due to the absence of suitable alloy references, there is some uncertainty in the determined GaO_*x*_ contents, which is likely overestimated.

**Figure 2:**
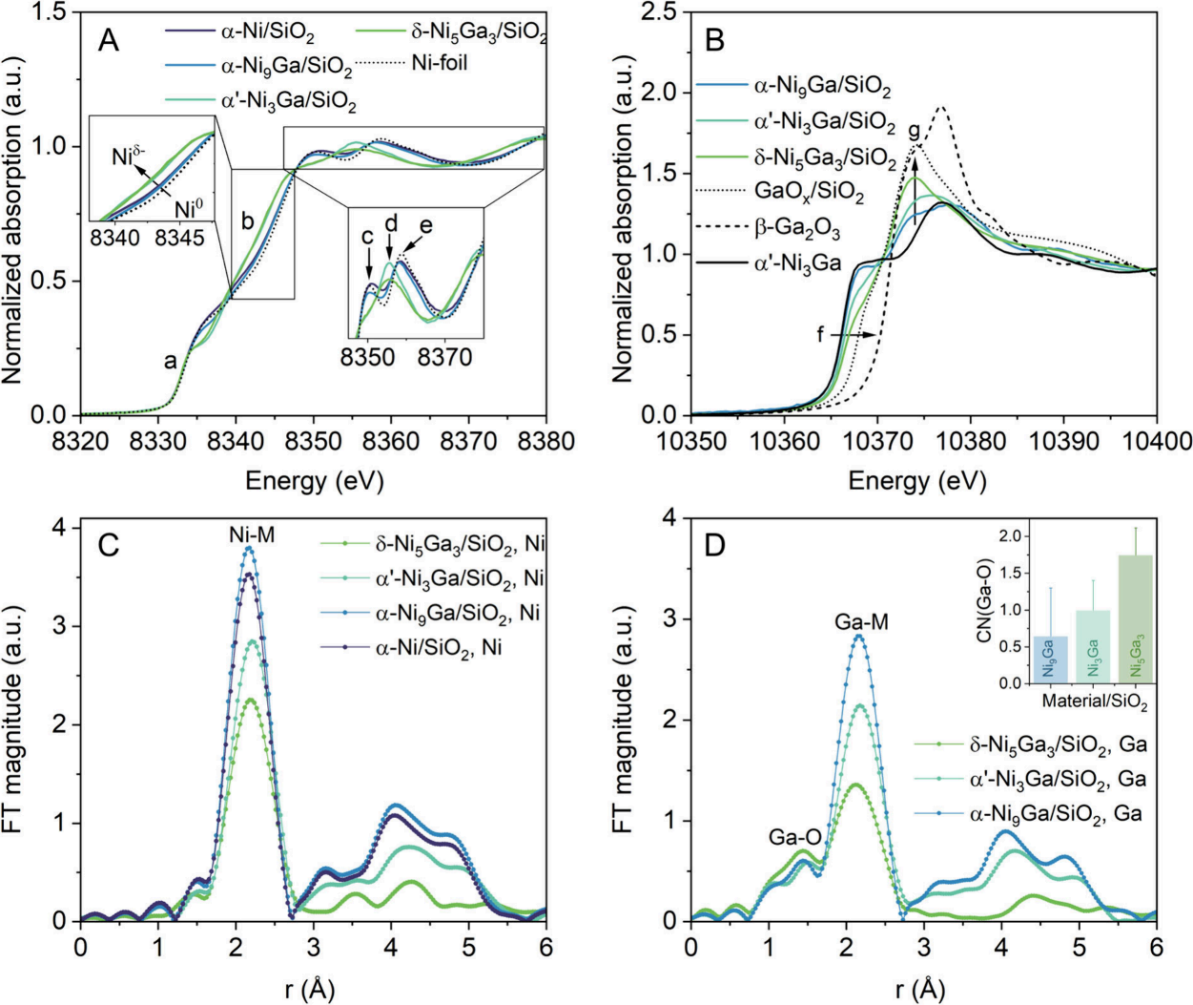
XAS data of the activated (700 °C, H_2_) catalysts (data collected after activation and cooling down to 50 °C in 1 bar H_2_). (A) Ni K-edge XANES of the catalysts studied and Ni-foil reference. The left inset shows a shift of the absorption edge position for α’-Ni_3_Ga/SiO_2_ and δ-Ni_5_Ga_3_/SiO_2_ compared to α-Ni_9_Ga/SiO_2_, α-Ni/SiO_2_, and the Ni-foil. The right inset highlights the white line features of the three catalysts explored, where *c* and *e* denote features associated with α-Ni_9_Ga and α-Ni structures while feature *d* is linked to α’-Ni_3_Ga and δ-Ni_5_Ga_3_ structures. (B) Ga K-edge XANES of the catalysts studied and the GaO_*x*_/SiO_2_, β-Ga_2_O_3_, and α’-Ni_3_Ga references. The arrows highlight the shift of the absorption edge position *f* toward higher energies and the increase in the white line intensity *g* with increasing Ga content in the catalysts. The inset shows the relative GaO_*x*_ content (GaO_*x*_/Ga_tot_ * 100%) in the catalysts as determined by LCF of the XANES data. (C) Ni and (D) Ga K-edge EXAFS data [magnitude of the FTs of the k^2^ weighted EXAFS­(k) vs the interatomic distance r not corrected for the phase-shift] with labeled Ga–O and Ni/Ga-M (M= Ni, Ga) shells. The inset in Figure D shows the fitted Ga–O coordination numbers.

The local structure of Ni and Ga in the activated catalysts was examined using EXAFS analysis. The Ni K-edge EXAFS oscillations and their Fourier transforms (FTs) are presented in Figures 2C and S20, respectively. The FTs show Ni-M (M = Ni, Ga) coordination spheres in the range 1.5–2.7 Å (not corrected for the phase-shift); no Ni–O sphere was observed. The Ga K-edge EXAFS showed a Ga–O coordination sphere at ca. 1.5 Å, and Ga-M spheres in the range 1.6–2.7 Å in line with the presence of gallium species in both an oxidic form and alloyed with nickel, as indicated by XANES analysis. The EXAFS data of the different catalysts were modeled considering one average Ni-M, one average Ga-M (M = Ni/Ga) and one Ga–O sphere. The fitting values obtained are summarized in Table S7 and confirmed the formation of Ni–Ga alloys in α-Ni_9_Ga/SiO_2_, α’-Ni_3_Ga/SiO_2_, and δ-Ni_5_Ga_3_/SiO_2_ as well as the presence of residual GaO_*x*_ phases in these catalysts. The fitted CN­(Ga–O) increased in the order α-Ni_9_Ga/SiO_2_ < α’-Ni_3_Ga/SiO_2_ < δ-Ni_5_Ga_3_/SiO_2_ (inset in Figure 2D), in agreement with the trend of an increasing quantity of GaO_*x*_ species with increasing Ga content as determined by XANES LCF analysis. Notably, no Ga–O–Ga scattering path associated with the crystalline Ga_2_O_3_ phase(s) was clearly detected, indicating that the GaO_*x*_ phase lacks long-range coherence and is likely dispersed on the SiO_2_ support. However, the possibility of some Ga–O-M linkages (e.g., due to GaO_*x*_ clusters) cannot be entirely ruled out, as they may fall below the detection limit.

In summary, PDF and XAS analyses confirmed that the activated catalysts contained the targeted nanocrystalline Ni–Ga alloy phases, viz. δ-Ni_5_Ga_3_ in δ-Ni_5_Ga_3_/SiO_2_, α’-Ni_3_Ga in α’-Ni_3_Ga/SiO_2_, and α-Ni_9_Ga in α-Ni_9_Ga/SiO_2_. In all catalysts, Ni was fully incorporated into the alloy structures. The electronic structure of Ni in α’-Ni_3_Ga/SiO_2_, and δ-Ni_5_Ga_3_/SiO_2_ is different from that of a Ni metal or a Ni-rich alloy, owing to an electronic interaction between Ni and Ga in the intermetallic α’-Ni_3_Ga and δ-Ni_5_Ga_3_ structures (i.e., Ni^δ‑^ sites). Ga was predominantly present in the Ni–Ga alloy but also existed as a GaO_*x*_ phase. Although XAS and d-PDF are not inherently surface-sensitive techniques, they have provided relevant insights into the local structure and oxidation states of the catalysts studied. We encourage future efforts to develop and apply in situ or operando X-ray photoelectron spectroscopy (XPS) methodologies on model systems to assess surface-bulk composition differences and their impact on catalytic performance.

### CO_2_ Hydrogenation Performance

The CO_2_ hydrogenation performance (product formation rates, selectivities, and CO_2_ conversion) of all catalysts was assessed in a laboratory fixed-bed reactor. Prior to the CO_2_ hydrogenation experiments, the catalysts were activated in a H_2_ flow at 1 bar at 700 °C for 1 h, followed by cooling to 230 °C in 10% H_2_/N_2_. Catalytic tests were performed at 25 bar using a gas flow with a composition CO_2_:N_2_:H_2_ = 1:1:3 (GHSV = 60 L^–1^ gcat^–1^). Additional experiments at a reaction pressure of 1 bar were performed to compare the catalysts tested here with previous works.^9^ The obtained product formation rates, selectivities (methanol: S_MeOH_, CO: S_CO_, and CH_4_: S_CH4_), and CO_2_ conversion are reported in Table S8 of the Supporting Information. Given that Ga is typically considered to be a promoter,^15,16^ the product formation rates were normalized by the Ni loading. Table S8, however, also reports product formation rates normalized by the total metal (Ni+Ga) content and catalyst mass. In the following, our discussion focuses primarily on the product formation and CO_2_ conversion rates normalized by the Ni content (Figure 3A), yet very similar trends were observed when normalizing by Ni+Ga (Figure S26). Also, since the trends in methanol formation rate and selectivity are independent of the reaction pressures tested (Figure S24), our discussion will focus on the results obtained at 25 bar. As expected, the CO_2_ conversion rates, methanol selectivity, and formation rates are higher at 25 bar compared to 1 bar (Table S8).

**Figure 3:**
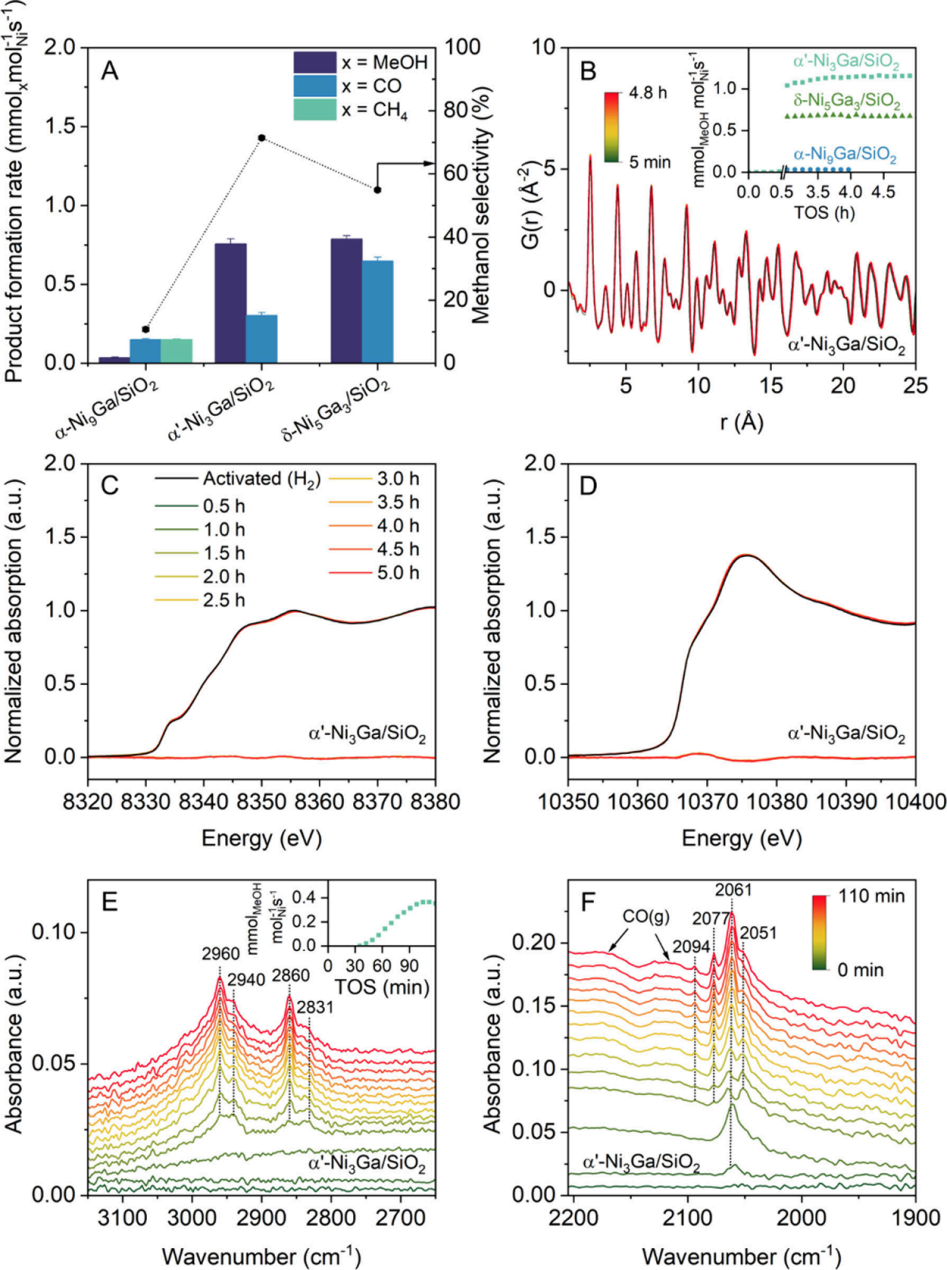
(A) Ni-normalized product formation rates and methanol selectivities (all averaged over 6 GC points/3h) of the different catalysts tested. Conditions: 25 bar, CO_2_:H_2_:N_2_ = 1:3:1 at 230 °C (GHSV = 60 L h^–1^ gcat^–1^). (B) d-PDF of α’-Ni_3_Ga/SiO_2_ over 4.8 h TOS collected at 20 bar CO_2_:H_2_:N_2_ = 1:3:1 at 230 °C in a capillary reactor. The inset shows the methanol formation rates of δ-Ni_5_Ga_3_/SiO_2_, α’-Ni_3_Ga/SiO_2_, and α-Ni_9_Ga/SiO_2_ measured during total scattering/PDF experiments. (C) Ni K-edge XAS of α’-Ni_3_Ga/SiO_2_ collected under the same conditions as in (B), including the difference curves with respect to the initial data set. (D) Ga K-edge XAS collected under the same conditions as in (B–C). (E) Operando DRIFT spectra collected on α’-Ni_3_Ga/SiO_2_ at 230 °C after switching from 20 bar N_2_ (TOS = 0 min) to 20 bar CO_2_:H_2_:N_2_ = 1:3:1. Spectral region in which bands due to C–H stretching vibrations are observed. (F) Spectral region in which band due to C–O stretching vibrations are observed.

In addition, kinetic analyses were performed using the α-Ni_9_Ga/SiO_2_, α’-Ni_3_Ga/SiO_2_, and δ-Ni_5_Ga_3_/SiO_2_ catalysts to gain insight into the reaction mechanisms underlying the formation of methanol, CO, and methane. The corresponding results are presented in Figure S27. Apparent activation energies (E_a_) for methanol formation and the RWGS reaction were determined from catalytic tests conducted at varying temperatures. The E_a_ values for methanol formation over α’-Ni_3_Ga/SiO_2_ and δ-Ni_5_Ga_3_/SiO_2_ were similar—49.1 and 48.4 kJ/mol, respectively—suggesting a shared reaction pathway. In contrast, methanol formation rates over α-Ni_9_Ga/SiO_2_ were too low to determine a reliable E_a_. For CO formation, the E_a_ values were comparable across all three catalysts: 68.1, 67.7, and 69.0 kJ/mol for α-Ni_9_Ga/SiO_2_, α’-Ni_3_Ga/SiO_2_, and δ-Ni_5_Ga_3_/SiO_2_, respectively. These values are significantly higher than those for methanol formation, which supports the high methanol selectivities observed for α’-Ni_3_Ga/SiO_2_, and δ-Ni_5_Ga_3_/SiO_2_ (Figure 3).

At 25 bar, α’-Ni_3_Ga/SiO_2_ reached a methanol formation rate of 0.76 mmol_MeOH_ mol_Ni_^–1^ s^–1^ and a methanol selectivity of 71% at 0.4% CO_2_ conversion (X_CO2_), while δ-Ni_5_Ga_3_/SiO_2_ yielded a similar methanol formation rate of 0.79 mmol_MeOH_ mol_Ni_^–1^ s^–1^, yet at a lower methanol selectivity of 55% at a similar X_CO2_ of 0.4%. In contrast, α-Ni_9_Ga/SiO_2_ produced only small amounts of methanol (ca. 0.03 mmol mmol_MeOH_ mol_Ni_^–1^ s^–1^), forming methane via the Sabatier reaction. The methane selectivity of α-Ni_9_Ga/SiO_2_ was 44% at a X_CO2_ of 0.2% which was considerably lower than that of α-Ni/SiO_2_ (96% at a X_CO2_ of 1.1%), in line with previous studies reporting a high activity and selectivity of Ni in the Sabatier reaction.^21^ The high methanol selectivity (at 200–250 °C) of δ-Ni_5_Ga_3_/SiO_2_ as observed here, is consistent with previous works.^9,10,14,51,52^ However, our results differ from previous literature in that the α’-Ni_3_Ga phase exhibited not only a comparable methanol rate but also a significantly higher methanol selectivity than δ-Ni_5_Ga_3_/SiO_2_, while previous studies have reported a low methanol productivity and high methane formation for the α’-Ni_3_Ga phase.^9–11^ The discrepancy between the methanol productivity and selectivity observed here and the previously reported results may stem from differences in the compositional homogeneity of the nanoparticles, as well as a potential misidentification of the structure of the catalyst. In view of these discrepancies, a clear understanding of the structural differences between α’-Ni_3_Ga and Ni-rich alloy nanoparticles is critical to derive structure-performance relationships in the Ni–Ga system. Our synchrotron-based XRD data showed the presence of the supercell reflections of the α’-Ni_3_Ga phase, providing unequivocal evidence for the presence of this phase in the activated catalysts. However, it is critical to verify that the structures identified in the activated catalysts do not change under CO_2_ hydrogenation conditions. Hence, in the following, we will investigate the structural stability of the catalysts under reaction conditions, in particular focusing on α’-Ni_3_Ga/SiO_2_ as the most active and controversial catalyst.

### Catalysts’ Structure under CO_2_ Hydrogenation Conditions

To evaluate whether the structure of the Ni–Ga/SiO_2_ catalysts remained stable under CO_2_ hydrogenation conditions with respect to the activated state, operando XAS and XRD/d-PDF studies were performed. In these experiments, the in situ activated catalysts (Figures 1 and 2) were pressurized to 20 bar in N_2_ at 230 °C and subsequently exposed to the reaction mixture (CO_2_:H_2_:N_2_ = 1:3:1) at 20 bar, with either XRD/d-PDF data or XAS data at the Ni and Ga K-edges being collected in separate experiments. The online gas analyses for each catalyst during operando d-PDF are shown in Figure 3B. When comparing the methanol formation rates of the different catalysts in the operando XAS and XRD/d-PDF experiments we observed the same trend as in the laboratory fixed bed reactors, i.e. the methanol formation rates decreased in the following order: α’-Ni_3_Ga/SiO_2_ < δ-Ni_5_Ga_3_/SiO_2_ < α-Ni_9_Ga/SiO_2_.

The d-PDF and XAS (Ga and Ni K-edges) data acquired over 5 h of time on stream (TOS) for the most active catalyst, viz. α’-Ni_3_Ga/SiO_2_, are plotted in Figure 3B-D. The corresponding plots for δ-Ni_5_Ga_3_/SiO_2_ and α-Ni_9_Ga/SiO_2_ are presented in Figures S8 and S14 of the Supporting Information. The operando d-PDF data did not reveal any changes in the phase of the nanoparticles in any of the catalysts tested. A detailed analysis of the unit cell parameters of the catalyst α’-Ni_3_Ga/SiO_2_ showed a small increase in the unit cell volume by ca. 0.3% within the first 1 h of TOS. One possible explanation for this observation is the thermal expansion of the nanoparticles due to the exothermic nature of the methanol formation reaction. However, assessing the precise nature of this expansion remains challenging. No discernible changes were observed in the less active catalysts, i.e. δ-Ni_5_Ga_3_/SiO_2_ or α-Ni_9_Ga/SiO_2_ (Figure S10 and Table S6). Importantly, the Ga K-edge and Ni K-edge XANES exhibited negligible changes (i.e., within the statistical variations of the measurements) under reaction conditions, suggesting that the oxidation state and local coordination of Ni and Ga remain unaltered with respect to their state after activation (Figure S14 and S15). Thus, operando PDF and XAS confirmed that the catalyst with the highest methanol productivity and selectivity (i.e., α’-Ni_3_Ga/SiO_2_) contains under CO_2_ hydrogenation conditions indeed an α’-Ni_3_Ga phase in addition to GaO_*x*_ species.

### Probing Possible Redox Dynamics under CO_2_ Hydrogenation Conditions

As in related catalyst formulations (i.e., Pd–Ga, Cu–Ga)^53^ a high methanol formation activity has been attributed to favorable redox dynamics under CO_2_ hydrogenation conditions, we probed whether exposing the catalyst to alternating H_2_ or CO_2_ atmospheres can alter the oxidation states of Ga and Ni.^54–56^ Specifically, we performed Ni and Ga K-edges XAS experiments in which the gas atmospheres were switched between H_2_ (20 bar) and CO_2_ (20 bar), after the catalysts had been exposed for 5 h to CO_2_ hydrogenation conditions (20 bar, 230 °C; CO_2_:H_2_:N_2_ = 1:3:1). The most active catalyst in this study, α’-Ni_3_Ga/SiO_2_, was selected for this experiment. We observed that when switching from a CO_2_ hydrogenation atmosphere to H_2_, there were no changes in the Ni or Ga K-edge XANES data. However, when switching from H_2_ to CO_2_, there was a very small increase in the white line intensity of the Ga K-edge XANES (feature *g* in Figure 2B), while no visible changes were detected in the Ni K-edge spectra. Tracking the intensity of feature *g* during the transient experiment revealed, however, that these changes were close to statistical noise (see Supporting Information section 4, Figure S16). Therefore, our findings suggest a high structural and chemical stability of the catalyst even under oxidizing conditions, with only very small changes in the Ga K-edge XANES signature that are likely due to interactions between surface Ga and CO_2_; yet these changes are too small to be considered significant. We note that such subtle redox responses might be enhanced using techniques like modulation excitation spectroscopy or under different reaction conditions (e.g., CO_2_ pressure or temperature), as well as with different Ni:Ga ratios in the alloy. While the observed redox shifts are minor, they suggest that surface Ga species may undergo reversible interaction with CO_2_. Overall, α’-Ni_3_Ga remains structurally robust, with only limited Ga redox dynamics occurring likely at surface sites.

To further evaluate the long-term performance of α’-Ni_3_Ga/SiO_2_, we conducted a catalytic test over an extended period of 9 h on stream. While the catalysts demonstrated structural stability and catalytic stability during operando XAS and d-PDF experiments, a small but gradual decline in activity was observed in the more extended fixed-bed test. Specifically, methanol productivity declined by 22% over 9 h of TOS, while methanol selectivity remained stable at 68% (Figure S28). The difference in testing conditions may partially explain this discrepancy in catalyst stability in the different setups. The operando synchrotron experiments were conducted over a shorter duration and at a lower gas hourly space velocity (GHSV, 10 L g cat^–1^ h^–1^) compared to the fixed-bed reactor (60 L g cat^–1^ h^–1^). Notably, there is a slower gas exchange in the capillary reactor (see Figure S6). To investigate the reversibility of the observed deactivation, we subjected the catalyst to a reactivation treatment in H_2_ treatment at 700 °C. This treatment restored 95% of the initial activity, indicating that the deactivation is not due to irreversible sintering, but is more likely caused by reversible processes such as surface oxidation.

### Surface Adsorbates on α’-Ni_3_Ga/SiO_2_ under CO_2_ Hydrogenation Conditions

To investigate key intermediates adsorbed on the catalyst surface under CO_2_ hydrogenation conditions, the most active catalyst for methanol formation identified in this work, i.e. α’-Ni_3_Ga/SiO_2_, was interrogated by operando DRIFTS (230 °C, 20 bar, CO_2_:H_2_:N_2_ = 1:3:1). The gas composition at the outlet of the DRIFTS cell was determined by GC (inset in Figure 3E), confirming methanol production during the experiment. For reference, operando DRIFTS data were also collected on α-Ni/SiO_2_. The assignment of the observed IR bands is summarized in Table S9.

Under steady-state CO_2_ hydrogenation conditions, we observed methoxy (CH_3_O*) and CO* species on α’-Ni_3_Ga/SiO_2_ (Figure 3E and F).^18^ In the ν­(C–O) region (1900–2200 cm^–1^) of the IR spectra, the band at 2061 cm^–1^ was assigned to CO* that is adsorbed linearly on Ni, [CO*­(Ni)]. This band was red-shifted compared to the CO*­(Ni) band observed in α-Ni/SiO_2_ (2067 cm^–1^) (Figure S30). This shift is in line with the presence of Ni^δ−^ sites in α’-Ni_3_Ga/SiO_2_ which enhance electron back-donation into the antibonding π orbitals of CO. This observation confirms that the (surface) electronic structure of Ni is altered upon the formation of the intermetallic α’-Ni_3_Ga phase, in agreement with d-PDF and XANES analyses discussed earlier. Additionally, the band located at 2051 cm^–1^ was assigned to nickel carbonyl species (tetracarbonyl or subcarbonyls), Ni­(CO)_n_ (n = 1–4), whereas the additional bands observed in the region 2077 −2200 cm^–1^ are due to the presence of gaseous CO and CO_2_. Turning to the evolution of the bands in the ν­(C–H) region (2700–3100 cm^–1^) during the operando DRIFTS experiments (Figure 3E). The bands at 2860 and 2960 cm^–1^ are assigned to the ν­(C–H) vibration of methoxy adsorbed onto SiO_2_, viz. CH_3_O*­(SiO_2_), based on previous literature data^3^ and a reference experiment in which methanol was adsorbed onto SiO_2_ (see section 6 in the Supporting Information and Figure S29). Weaker bands at 2831 and 2940 cm^–1^ are likely due to methoxy adsorbed on the α’-Ni_3_Ga nanoparticles or bidentate formate species.^3,56^ To distinguish between these two possible species, their respective ν­(C–O) vibrations could provide more insight. However, due to the low infrared throughput below 2000 cm^–1^, the signal-to-noise ratio in this region was poor, hindering the detection of the characteristic ν­(C–O) vibration of formate.^3^ Previous studies have reported a weak band at 2898 cm^–1^ on Ni–Ga/SiO_2_ catalysts under CO_2_ hydrogenation conditions, which was attributed to bidentate formate adsorbed on GaO_*x*_. It is possible that in the present operando study, the detection of such band may have been hindered by the overlap with the CH_3_O*­(SiO_2_) bands.

Thus, our DRIFTS experiments identified the formation of methoxy and CO* intermediates under CO_2_ hydrogenation conditions and the presence of a Ni–Ga alloy in α’-Ni_3_Ga/SiO_2_ in which Ga donates electronic charge to Ni (Ni^δ−^ species), as evidenced by the energy shift of the band associated with linearly adsorbed CO* on Ni (when compared to the corresponding band for α-Ni/SiO_2_). This electronic interaction between Ni and Ga (Ni^δ−^Ga^δ+^) agrees with XANES analysis (Figure 2A) and with the theoretical Bader charge analysis (Table S14 and accompanying discussion). Yet, to obtain a detailed understanding of the prevailing methanol formation pathways and the role of the different surface alloys and the GaO_*x*_ species in methanol formation DFT calculations were performed. However, we note that complementary insight could also be obtained from future studies focused on kinetic analysis using kinetic isotope effects (KIEs).^57^ Such approaches may enhance the experimental observation of molecular mechanisms under reaction conditions and further clarify the origin of the observed catalytic behavior.

### Density Functional Theory Calculations: Role of Ni–Ga Surface Geometry and Ni–Ga/GaO*_x_* Interfaces

DFT calculations were performed to provide insight into the role of the specific Ni–Ga alloys and GaO_*x*_ on the formation of methanol and undesirable side products (such as methane) under CO_2_ hydrogenation conditions. Specifically, we modeled slabs of α-Ni, α’-Ni_3_Ga, and δ-Ni_5_Ga_3_, whereby both flat [α-Ni(111), α’-Ni_3_Ga­(111), and δ-Ni_5_Ga_3_(221)] as well as stepped [α-Ni(211), α’-Ni_3_Ga­(211), and δ-Ni_5_Ga_3_(211)] surfaces were considered (further details are available in the Supporting Information section 7). It is important to discuss both surface types, as a mixture of both is present in the experimental Ni–Ga nanoparticle systems.^58^ Our surface energy calculations showed that flat surfaces are generally more thermodynamically stable whereas stepped surfaces are less stable, but more reactive (Table S12).

### CO Formation

First, we analyzed the energy profiles for a key side reaction, i.e. the reverse water-gas shift reaction (RWGS) for the different surface models considered here (Figure S31). Mechanistically, the RWGS is initiated by cleavage of the C–O bond of CO_2_ to form CO* and O*, followed by the formation of H_2_O from O* through its hydrogenation, and desorption of CO* into the gas phase. On flat Ni and flat Ni–Ga alloyed surfaces, C–O bond cleavage was found to be exoenergetic (*ΔE*_*r*_ < 0), with reaction energies (*E*_*r*_) for CO* and O* formation of −1.24 eV for α-Ni(111), −0.94 eV for α’-Ni_3_Ga­(111), and −0.74 eV for δ-Ni_5_Ga_3_(221), as shown in Figures 4A and S31. Interestingly, when the presence of GaO_*x*_ was considered, i.e. GaO_*x*_/α’-Ni_3_Ga, C–O bond cleavage became endoenergetic on both flat and stepped α’-Ni_3_Ga surfaces. H_2_O formation is energetically favorable (exoenergetic) on α’-Ni_3_Ga­(111) and δ-Ni_5_Ga_3_(221) and slightly endoenergetic (*ΔE*_*r*_ > 0), on α-Ni(111). As water forms, O* is consumed, leaving CO* adsorbed on the surface. Adsorbed CO* is notably most stable on Ni-rich surfaces, such as α’-Ni_3_Ga­(111) and α-Ni(111). CO desorption was endoenergetic for all surfaces, with α’-Ni_3_Ga­(111) requiring the highest energy difference, *ΔE*_*r*_. An important observation was that for the GaO_*x*_/α’-Ni_3_Ga­(111) system, *ΔE*_*r*_ for CO desorption decreased significantly compared to α’-Ni_3_Ga­(111).

**Figure 4:**
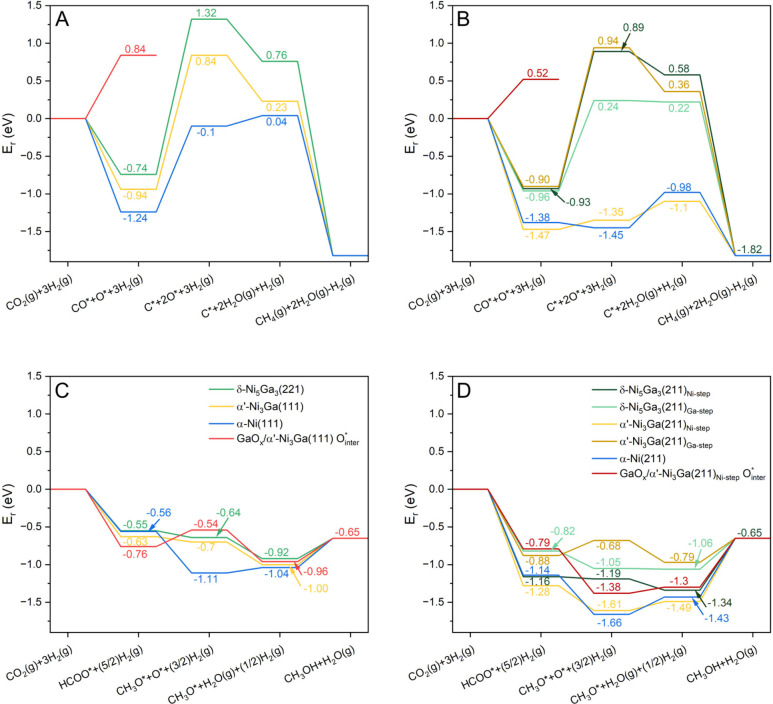
(A, B) Reaction energies (*E*_*r*_) for the reaction of CO_2_ and 3H_2_ to form CH_4_ on a series of flat (A) and stepped (B) catalyst surfaces. The E_r_ for the GaO_*x*_/α’-Ni_3_Ga system beyond the CO_2_ dissociation step is not shown because C* readily desorbs into the gas phase rather than adsorbing on the GaO_*x*_ cluster. This highlights the role of GaO_*x*_ in limiting methane formation. (C, D) *E*_*r*_ for the reaction of CO_2_ and 3H_2_ to form CH_3_OH from HCOO* on a series of flat (C) and stepped (D) catalyst surfaces.

Turning to stepped surfaces (Figure S31), their reactivity for C–O bond cleavage was generally higher than that for the corresponding flat surfaces, with the key intermediates CO* and O* displaying much stronger adsorption energies. Similar to flat surfaces, CO* adsorbs most stably on Ni-rich surfaces such as α’-Ni_3_Ga­(211)_Ni‑step_ and α-Ni(211). Further, CO desorption on stepped surfaces was more endoenergetic than on flat surfaces, with the highest *ΔE*_*r*_ observed for α’-Ni_3_Ga­(211)_Ni‑step_. Similar to the flat surfaces, the GaO_*x*_/α’-Ni_3_Ga­(211)_Ni‑step_ system exhibited the lowest *ΔE*_*r*_ *,* indicating a reduced binding energy for CO when GaO_*x*_ is present. Overall, CO_2_ dissociation into CO* and O* was energetically favorable on all surfaces, with stepped surfaces being more favorable than flat ones, except for the GaO_*x*_/α’-Ni_3_Ga system. Moreover, CO* desorption was unfavorable across all surfaces, with CO*being particularly well-stabilized on Ni-rich surfaces (α-Ni and α’-Ni_3_Ga).

### Methane Formation

Turning to the second side reaction under CO_2_ hydrogenation conditions (observed in particular over in Ni-rich catalysts), i.e. methane formation, a pathway that start similar to the RWGS pathway was considered, i.e., C–O bond cleavage of CO_2_ yielding CO* and O*; CO* is cleaved further yielding C* and O* while H_2_O forms via the hydrogenation of O*; the hydrogenation of C* yields CH_4_ which is ultimately desorbed into the gas phase. The corresponding energy profiles for the Sabatier reaction on flat and stepped surfaces are shown in Figures 4A and 4B, respectively.

On all flat surfaces, CO* cleavage is highly endoenergetic, with α-Ni(111) being the most favorable surface (*ΔE*_*r*_ = 1.14 eV), followed by α’-Ni_3_Ga­(111) (*ΔE*_*r*_ = 1.78 eV) and δ-Ni_5_Ga_3_(221) (*ΔE*_*r*_ = 2.06 eV) (Figure 4A). These results indicate that alloying Ni with Ga renders CO* dissociation energetically less favorable which translates into less methane formation. On stepped surfaces, CO* cleavage is more favorable than on flat surfaces but remains endoenergetic for Ga-rich surfaces. Furthermore, CO* cleavage is exoenergetic on α-Ni(211) and slightly endoenergetic on α’-Ni_3_Ga­(211)_Ni‑step_. The hydrogenation of C* and CH_4_ desorption is highly exoenergetic across all surfaces, indicating that CO* cleavage is the critical factor that determines the selectivity to methane versus CO in CO_2_ hydrogenation. C* adsorption on a GaO_*x*_ cluster was found to be highly unfavorable, leading to rapid desorption into the gas phase. To summarize, the critical step for methane formation is the dissociation of CO* into C*, which was most endoenergetic over Ga-rich surfaces. This indicates that alloyed Ga plays a crucial role in hindering methanation in Ni–Ga catalysts. Conversely, on Ni-rich catalysts such as α-Ni(211) and α’-Ni_3_Ga­(211)_Ni‑step_, C–O bond cleavage of CO* is either exoenergetic or slightly endoenergetic. In these systems, the critical step for methane formation is the hydrogenation of O* to H_2_O. Methane desorption is highly exoenergetic across all surfaces.

### Methanol Formation

It has been proposed that under CO_2_ hydrogenation conditions methanol can form via two pathways: the formate (HCOO*) or the RWGS (CO*) pathway (Figures 4 and S32).^17,18,59^ The formate pathway proceeds via the formation of HCOO* through the hydrogenation of CO_2_, which undergoes C–O bond cleavage and is subsequently hydrogenated to CH_3_O*. H_2_O is formed through hydrogenation of O* while CH_3_O* undergoes further hydrogenation to form CH_3_OH. Finally, CH_3_OH is desorbed into the gas phase. In the alternative RWGS pathway, CH_3_O* is formed directly from CO* through hydrogenation.

When comparing these two potential methanol formation pathways on Ni–Ga alloy surfaces, HCOO* formation was exoenergetic on both flat and stepped surfaces, making the formate route energetically favorable. Among the flat surfaces, α’-Ni_3_Ga­(111) showed the lowest energy for HCOO* formation (Δ*E*_*r*_ = −0.63 eV, Figure 4C), while stepped surfaces (Figure 4D) showed an even lower Δ*E*_*r*_ for HCOO* formation (Figure S32), in particular the Ni-rich terminations α’-Ni_3_Ga­(211)_Ni‑step_, δ-Ni_5_Ga_3_(211)_Ni‑step_, and α-Ni(211) with *E*_*r*_ values of −1.28, −1.16, and −1.14 eV, respectively.

Methoxy formation from HCOO* was also exoenergetic on flat surfaces and on most stepped surfaces, except for α’-Ni_3_Ga­(211)_Ga‑step_. In contrast, methoxy formation from CO* was endoenergetic on all flat Ni–Ga surfaces. However, on stepped surfaces methoxy formation from CO* became exoenergetic, except for α’-Ni_3_Ga­(211)_Ga‑step_. When comparing the *ΔE*_*r*_ for the two pathways on the stepped surfaces (Figure S32), it is clear that the HCOO* pathway was significantly more favorable, with |*ΔE*_*r*_ < 0| for CH_3_O* formation being nearly twice as high as the values for the CO* pathway on α’-Ni_3_Ga­(211)_Ni‑step_ and eight times as high on δ-Ni_5_Ga_3_(211)_Ni‑step_. These findings indicate that on both flat and stepped surfaces of Ni–Ga alloys the formate pathway is thermodynamically more favorable for methanol formation compared to the CO pathway. This is consistent with experimental studies on both unsupported and CeO_2_/ZrO_2_-supported Ni–Ga catalysts, where formate species were detected under CO_2_ hydrogenation conditions and identified as key intermediates in the formation of methanol.^18–20^ The final hydrogenation step

$$CH_{3}O^{*}+\;H_{2}O(g)+\frac{1}{2}H_{2}\to CH_{3}OH(g)+H_{2}O(g)$$

to CH_3_OH was endoenergetic across all systems. This step required less energy on Ga-rich surfaces, with the lowest *ΔE*_*r*_ of 0.31 eV being observed on the flat δ-Ni_5_Ga_3_(221) surface. On stepped surfaces, α’-Ni_3_Ga­(211)_Ga‑step_ showed the lowest *ΔE*_*r*_ of 0.32 eV for this step.

The presence of GaO_*x*_ influenced the formation energy of formate. On the flat α’-Ni_3_Ga­(111) surface, GaO_*x*_ rendered HCOO* formation more favorable, lowering Δ*E*_*r*_ from −0.63 eV on α’-Ni_3_Ga­(111) to −0.76 eV on GaO_*x*_/α’-Ni_3_Ga­(111). In contrast, on the highly reactive α’-Ni_3_Ga­(211)_Ni‑step_ surface, the stabilization of formate became less favorable with the addition of a GaO_*x*_ cluster [*E*_*r*_ of −0.79 eV for GaO_*x*_/α’-Ni_3_Ga­(211) vs −1.28 eV for α’-Ni_3_Ga­(211)_Ni‑step_]. The hydrogenation of HCOO* to CH_3_O* was exoenergetic on flat Ni and Ni–Ga alloy surfaces but became endoenergetic in the presence of GaO_*x*_. On stepped surfaces, GaO_*x*_ had the opposite effect, making CH_3_O* formation slightly more exoenergetic, with a *ΔE*_*r*_ of −0.59 eV for GaO_*x*_/α’-Ni_3_Ga­(211)_Ni‑step_ compared to −0.33 eV for α’-Ni_3_Ga­(211)_Ni‑step_. The hydrogenation of CH_3_O* to methanol and its subsequent desorption were rendered slightly more favorable by the presence of GaO_*x*_, with *ΔE*_*r*_ decreasing from 0.35 to 0.31 eV for α’-Ni_3_Ga­(111) and GaO_*x*_/α’-Ni_3_Ga­(111) and from 0.84 to 0.65 eV for α’-Ni_3_Ga­(211)_Ni‑step_ and GaO_*x*_/α’-Ni_3_Ga­(211)_Ni‑step_.

To verify further the results of the calculated formation pathways, the energy barriers for the two key transition states for methanol formation via the formate pathway^9^ were calculated on the α’-Ni_3_Ga­(111) and α’-Ni_3_Ga­(211)_Ni‑step_ models. These transition states are H–COO^⧧^ (leading to HCOO*) and H–HCOOH^⧧^ (leading to H_2_COOH*), and the corresponding calculated energy values are reported in Tables S15 and S16. The energy barriers for both transition states are lower on the stepped α’-Ni_3_Ga­(211)_Ni‑step_ surface, i.e., 0.49 eV for H–COO^⧧^ and 0.87 eV for H-HCOOH^⧧^ compared to 0.72 eV for H–COO^⧧^ and 1.05 eV for H-HCOOH^⧧^ for the flat α’-Ni_3_Ga­(111) surface. This observation aligns with the overall energetic profile for Ni_3_Ga­(211)_Ni‑step_, supporting further the conclusion that this surface is the most reactive for methanol formation. Although the energy barriers for the two key transition states are higher on α’-Ni_3_Ga­(111) than on α’-Ni_3_Ga­(211)_Ni‑step_, the results suggest that methanol formation is feasible on both surfaces. On the GaO_*x*_/alloy interface, we observed that H* tends to migrate away from CO_2_* and HCOO*, suggesting that on GaO_*x*_ these two transition states are associated with higher energy barriers. This indicates that the reaction steps proceeding through the two key transition states are more likely to proceed on the alloy rather than on the oxide cluster.

To summarize, DFT showed that methoxy, and thus methanol, formation is energetically more favored to occur via the formate pathway than the CO pathway on Ni–Ga alloy catalysts, independent of the Ni:Ga ratio and surface termination. The stabilization of HCOO* was highest for α’-Ni_3_Ga­(111) among the flat surfaces and on α’-Ni_3_Ga­(211)_Ni‑step_ for the stepped surfaces. Importantly, on both flat and stepped α’-Ni_3_Ga surfaces the presence of GaO_*x*_ destabilized CO* and C*, offering an explanation for the experimentally observed lower selectivities to CO and methane.

### CO_2_ Adsorption

Additionally, we assessed the energetics of CO_2_ adsorption which initiates the CO_2_ hydrogenation pathways. We further assessed whether GaO_*x*_ may facilitate CO_2_ activation and thereby promote methanol formation.^14,21^ Here, we compared the α’-Ni_3_Ga­(111) and α’-Ni_3_Ga­(211)_Ni‑step_ surfaces, both with and without the presence of GaO_*x*_ clusters. On the flat α’-Ni_3_Ga­(111) surface, the presence of GaO_*x*_ favored CO_2_ adsorption, with an *ΔE*_*r*_ of −0.3 eV compared to 0.11 eV without GaO_*x*_ (Figure S33). In contrast, on the stepped α’-Ni_3_Ga­(211)_Ni‑step_ surface, GaO_*x*_ had the opposite effect, rendering CO_2_ adsorption endoenergetic (*ΔE*_*r*_ = 0.13 eV), whereas it was exoenergetic in the absence of GaO_*x*_ (*ΔE*_*r*_ = −0.43 eV). These results demonstrate that GaO_*x*_ can significantly modify the energetics of CO_2_ adsorption, with its effect depending on the specific Ni–Ga alloy surface it interacts with. GaO_*x*_ can either enhance or reduce CO_2_ adsorption, which contrasts with previous studies suggesting a consistently more favorable CO_2_ adsorption in the presence of GaO_*x*_.

To summarize, DFT rationalizes the high activity and selectivity of α’-Ni_3_Ga by showing that the formation of key formate and methoxy intermediates is energetically more favorable over this phase compared to δ-Ni_5_Ga_3_, both on flat and stepped surfaces. Further, DFT highlights the key role of alloyed Ga species and the GaO_*x*_/Ni–Ga alloy interface in modulating the activity and selectivity of Ni–Ga-based catalysts for methanol synthesis.

## Conclusions

The work reported here aimed to identify the catalytically most active structure of Ni–Ga-based catalysts and its performance under CO_2_ hydrogenation conditions. The key insights from experimental and theoretical investigations are summarized in Figure 5. To this end, a series of silica-supported Ni–Ga catalysts was synthesized via hydrothermal deposition-precipitation and activated at 700 °C in H_2_, yielding phase-pure α-Ni_9_Ga, α’-Ni_3_Ga, or δ-Ni_5_Ga_3_ supported on SiO_2_. Operando PDF analysis confirmed the structural stability of the different phases under reaction conditions. Ni K-edge XAS revealed a charge transfer between Ga and Ni leading to Ni^δ−^(Ga^δ+^) sites (also confirmed by operando DRIFTS) in α’-Ni_3_Ga/SiO_2_ and δ-Ni_5_Ga_3_/SiO_2_; this charge transfer was absent in α-Ni_9_Ga/SiO_2_ (Ni^0^ states), in line with Bader charge analysis. Additional operando Ga K-edge XAS confirmed the presence of GaO_*x*_ species in all Ni–Ga catalysts. Our catalytic tests demonstrated high methanol formation rates and selectivities in α’-Ni_3_Ga/SiO_2_, hence challenging the previous view that α’-Ni_3_Ga is a poor methanol catalyst. Specifically, α’-Ni_3_Ga/SiO_2_ and δ-Ni_5_Ga_3_/SiO_2_ showed significantly higher methanol formation rates (0.76 and 0.79 mmol_MeOH_ mol_Ni_^–1^ s^–1^ respectively) than α-Ni_9_Ga/SiO_2_ and α-Ni/SiO_2_ (both 0.03 mmol_MeOH_ mol_Ni_^–1^ s^–1^). Furthermore, α’-Ni_3_Ga/SiO_2_ achieved the highest methanol selectivity (S_MeOH_ = 71%) outperforming δ-Ni_5_Ga_3_/SiO_2_ (S_MeOH_ = 55%), α-Ni_9_Ga/SiO_2_ (S_MeOH_ = 10%) and α-Ni/SiO_2_ and (S_MeOH_ = 2%). Notably, α’-Ni_3_Ga/SiO_2_ and δ-Ni_5_Ga_3_/SiO_2_ did not form methane.

**Figure 5:**
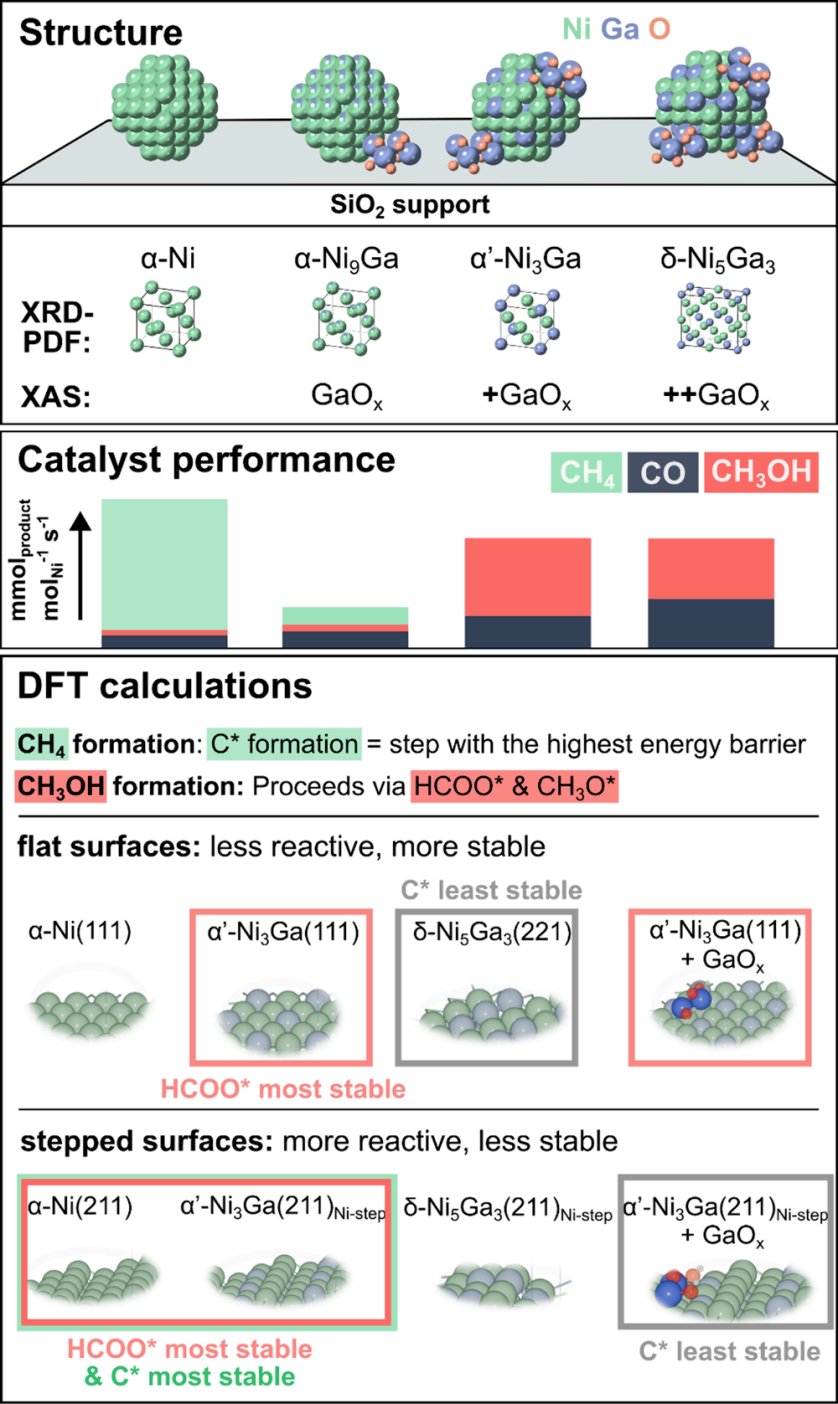
A graphical summary of this study’s main conclusions and the methodologies employed to reach them. Structural analysis using XRD-PDF revealed the nanocrystalline alloy structure, while Ga K-edge XAS identified the formation of GaO_*x*_ species. These species were correlated with variations in methanol selectivity and productivity, with the highest methanol productivity and selectivity observed for α’-Ni_3_Ga nanoparticles supported on SiO_2_ that also exhibited GaO_*x*_. DFT calculations provided insights into the stabilization trends of key reaction intermediates on Ni–Ga alloy surfaces compared to pure Ni, helping to elucidate structure–activity relationships.

These experimental findings were rationalized by DFT calculations, which showed that α’-Ni_3_Ga surfaces are more effective in stabilizing formate and methoxy intermediates, that are critical intermediates in the methanol synthesis pathway when compared to δ-Ni_5_Ga_3_. Further, the presence of GaO_*x*_ together with alloyed Ga in α’-Ni_3_Ga was shown to disfavor CO* and C* adsorption, thereby reducing methane formation and further contributing to this phase’s catalytic performance. These DFT results provide a theoretical framework explaining why α’-Ni_3_Ga is a more selective methanol synthesis catalyst than δ-Ni_5_Ga_3_.

## Supplementary Material



## Notes

Additional experimental details and XAS, X-ray total scattering/PDF, and DRIFTS data (PDF)

## Abbreviations

d-PDF: differential (or difference) pair distribution function analysis
DRIFTS: diffuse reflectance infrared Fourier transform spectroscopy
EXAFS: extended X-ray absorption fine structure
STEM-EDX: scanning transmission electron microscopy–element-dispersive X-ray spectroscopy
TOS: time on stream
XANES: X-ray absorption near-edge structure
XAS: X-ray absorption spectroscopy
