# Supplementary material for: How Does the Ni–Ga Alloy Structure Tune Methanol Productivity and Selectivity?
Source: ACS Catal. 2025 Aug 1;15(16):14252–66. doi: 10.1021/acscatal.5c02008 (PMC12362429; doi:10.1021/acscatal.5c02008)
Supplement: Supplementary file 1 [file cs5c02008_si_001.pdf]

Supporting Information

How does the Ni-Ga Alloy Structure Tune Methanol Productivity and Selectivity?

Nora K. Zimmerli[a]‡, Andrés F. Usuga[b]‡, Stefano Checchia[d], Aleix Comas-Vives[b,c*],
Christoph R. Müller[a*], and Paula M. Abdala[a*]

a ETH Zürich, Department of Mechanical and Process Engineering, Leonhardstrasse 21, 8092 Zurich, Switzerland
b Universitat Autònoma de Barcelona, Departament de Química, 08193 Cerdanyola del Vallès, Catalonia, Spain
c TU Wien, Institute of Materials Chemistry, Getreidemarkt 9/165, 1060 Vienna, Austria
d ESRF – The European Synchrotron, 71 Avenue des Martyrs, 38000 Grenoble, France
‡ These authors contributed equally to this work.

Contents

1. Catalyst synthesis 3
2. Electron microscopy 3
3. Operando X-ray total scattering experiments 4
4. Operando XAS experiments 9
5. CO_2 hydrogenation tests 20
6. DRIFTS 25
7. Density functional theory calculations 26
8. References 34

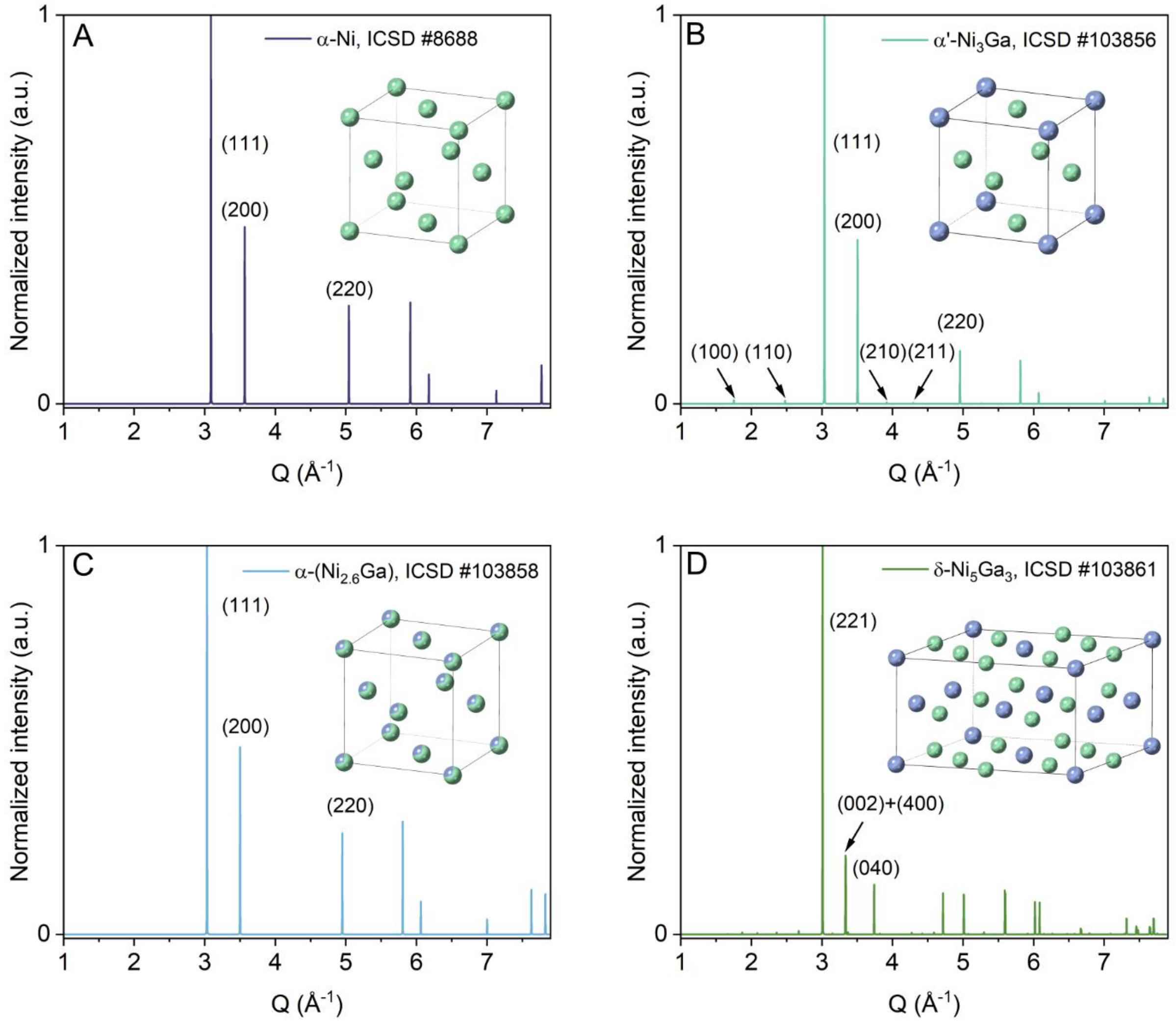

Figure S 1. Unit cells and diffraction patterns of α-Ni (A), α'-Ni_3Ga (B), α-($Ni_{2.6}Ga$) (C), and δ-Ni_5Ga_3 (D). Nickel atoms are shown in green, and gallium atoms are shown in blue.

Structure of α-(Ni,Ga) random alloy and α'-Ni_3Ga

In an α-(Ni,Ga) random alloy, the unit cell is face- centered cubic (fcc) with Ni and Ga occupying the same crystallographic site randomly. The crystal structure of α'-Ni_3Ga is described by the space group *P*m-3m (primitive cubic cell) with two distinct crystallographic sites whereby Ni occupies exclusively the face center sites and Ga the corner sites of the unit cell, corresponding to an ordered alloy or intermetallic. Both unit cells are illustrated in Figure S1.

1. Catalyst synthesis

Table S 1. Amounts of chemicals used for the catalyst synthesis.

Material	$Ni(NO_3)_2$ *$6H_2O$(g)	$Ga(NO_3)_3$ *$5.22H_2O$(g)	Urea (g)	Colloidal SiO_2, 50 wt% in H_2O (g)
α-Ni/SiO_2	0.261	0	2	2
α-Ni_9Ga/SiO_2	0.230	0.031		
α'-Ni_3Ga/SiO_2	0.151	0.103		
δ-Ni_5Ga_3/SiO_2	0.113	0.143		

2. Electron microscopy

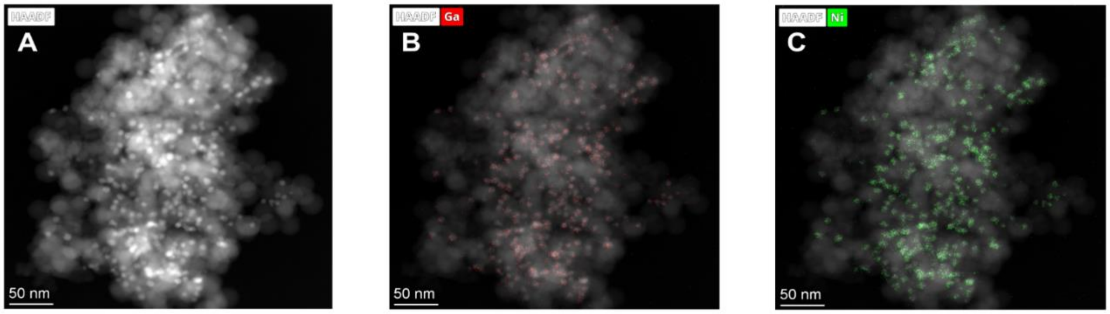

Figure S 2. Electron microscopy images of activated α'-Ni_3Ga/SiO_2: (A) HAADF-STEM image. (B) Ga EDX map. (C) Ni EDX map.

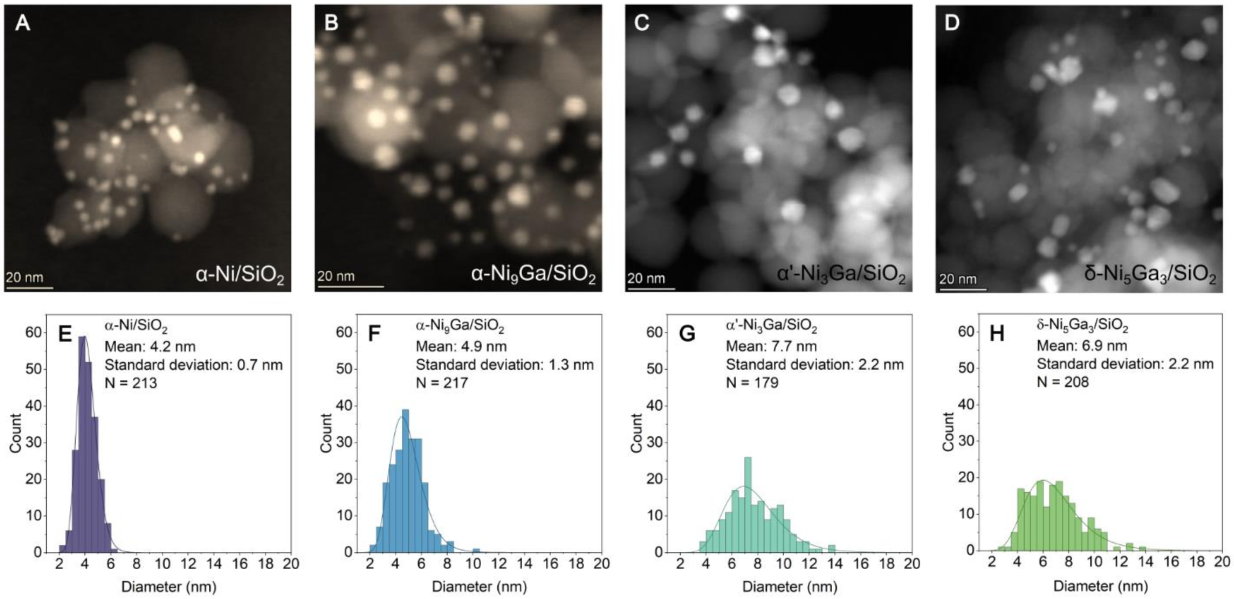

Figure S 3. Representative HAADF-STEM images and corresponding particle size distributions of the activated catalysts: (A, E) α-Ni/SiO_2, (B, F) α-Ni_9Ga/SiO_2, (C, G) α'-Ni_3Ga/SiO_2, and (D, H) δ-Ni_5Ga_3/SiO_2.

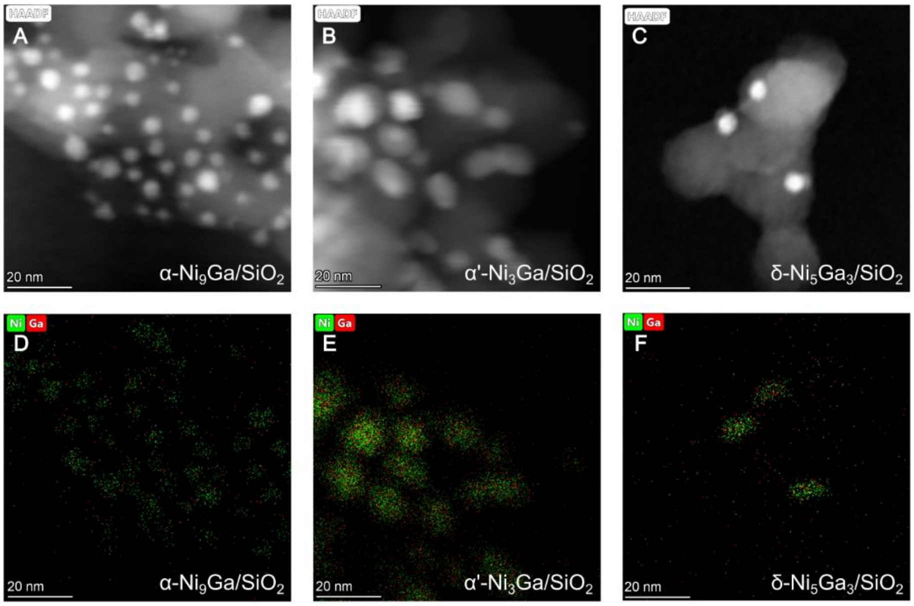

Figure S 4. HAADF-STEM images and corresponding EDX maps of the activated catalysts: (A, D) α-Ni_9Ga/SiO_2, (B, E) α'-Ni_3Ga/SiO_2, and (C, F) δ-Ni_5Ga_3/SiO_2.

Table S 2. Particle diameters (TEM), coherent size (PDF), and metal contents of the activated catalysts.[(a)] "±x" denotes the standard deviation from the mean extracted from the TEM particle size distribution and the fitting error of the PDF analysis, respectively.

**Material**	**Mean particle diameter[(a)] (nm)**		**Elemental analysis results (ICP-OES)**		
	**TEM**	**PDF**	**Ni (wt%)**	**Ga (wt%)**	**Ni:Ga molar ratio (-)**
α-Ni/SiO_2	4.2 ± 0.7	-	5.06 ± 0.03	-	∞
α-Ni_9Ga/SiO_2	4.9 ± 1.3	6.2 ± 0.1	4.16 ± 0.02	0.46 ± 0.01	10.70
α'-Ni_3Ga/SiO_2	7.7 ± 2.2	6.5 ± 0.1	2.74 ± 0.08	1.26 ± 0.05	2.58
δ-Ni_5Ga_3/SiO_2	6.9 ± 2.2	6.4 ± 0.2	2.52 ± 0.03	1.73 ± 0.05	1.73
GaO_x/SiO_2	-	-	-	0.49 ± 0.02	0

3. Operando X-ray total scattering experiments

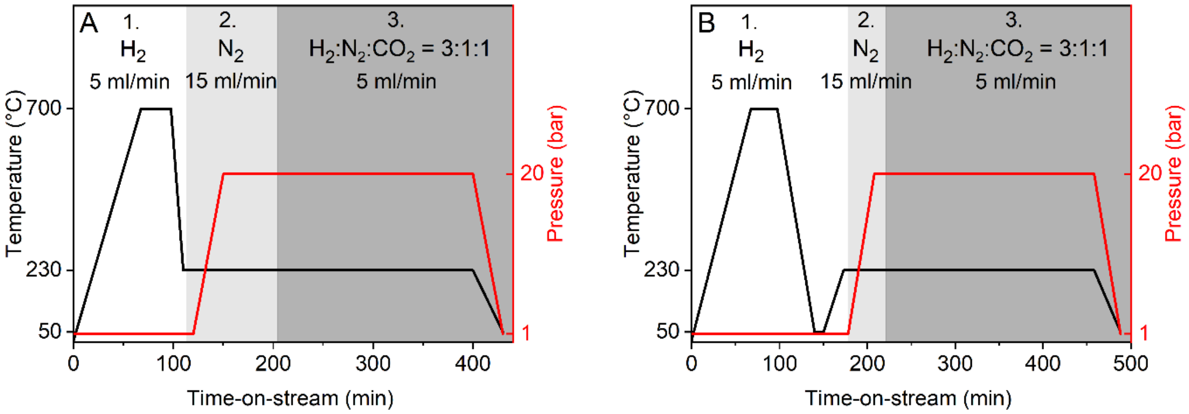

Figure S 5. Experiment conditions: (A) Operando X-ray total scattering. (B) XAS.

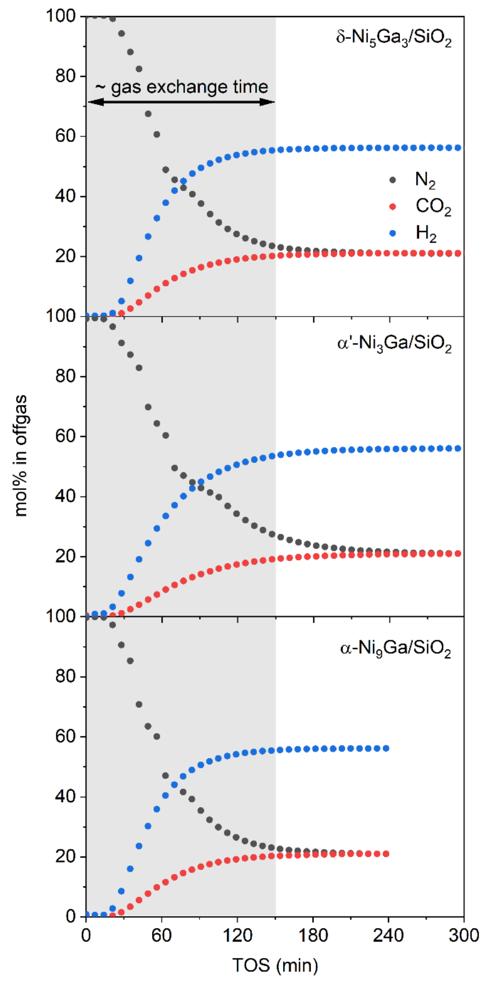

Figure S 6. GC data collected during the operando X-ray total scattering experiments. The gas exchange time was estimated to be approximately 2-2.5 hours after switching from 20 bar N_2 to a CO_2:H_2:N_2 mixture (1:3:1). The flow rate was 5 ml/min.

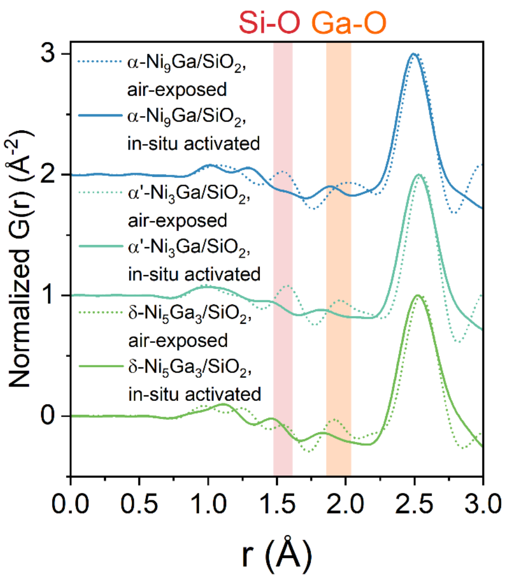

Figure S 7. Zoom in to the low-r region of the d-PDF of air-exposed (dotted lines) and in situ activated (solid lines) catalysts up to r = 3 Å. Red and orange shaded areas indicate where Si-O and Ga-O distances are expected.

Figure S7: Additional discussion

The presence of Ga-O pairs from GaO_x species is masked by the noise termination ripples and the relatively lower scattering contribution when compared to metal-metal pairs.

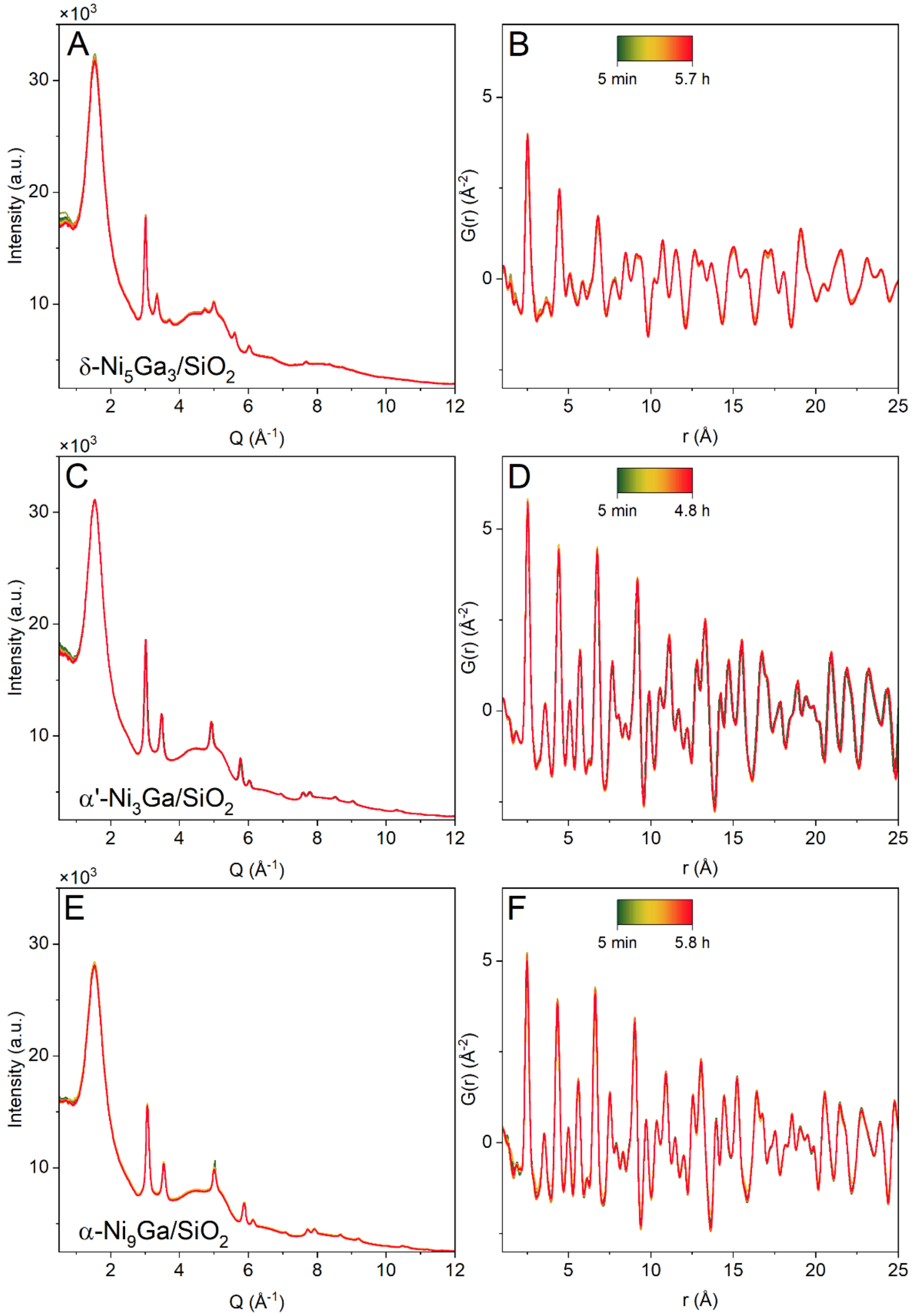

Figure S 8. Total scattering patterns and respective d-PDF. (A, B) δ-Ni_5Ga_3/SiO_2. (C, D) α'-Ni_3Ga/SiO_2. (E, F) α-Ni_9Ga/SiO_2. Data collected at 230 °C in 20 bar CO_2:H_2:N_2 = 1:3:1.

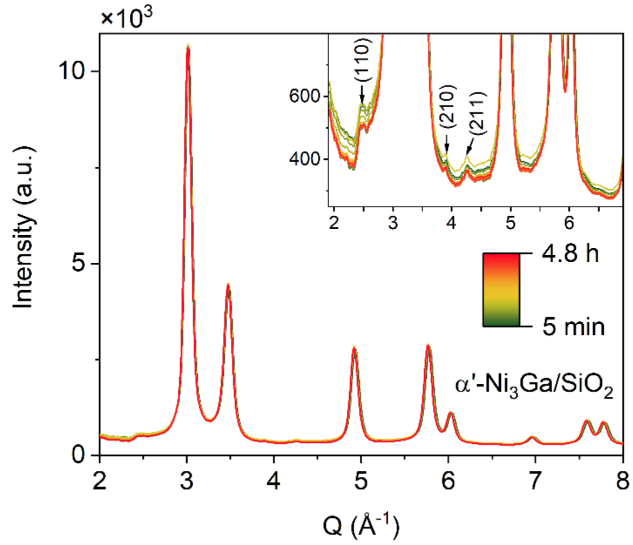

Figure S 9. SiO_2-subtracted total scattering pattern of α'-Ni_3Ga/SiO_2 under CO_2 hydrogenation conditions. The inset shows the position of some supercell reflections of α'-Ni_3Ga/SiO_2. Data collected at 230 °C in 20 bar CO_2:H_2:N_2 = 1:3:1.

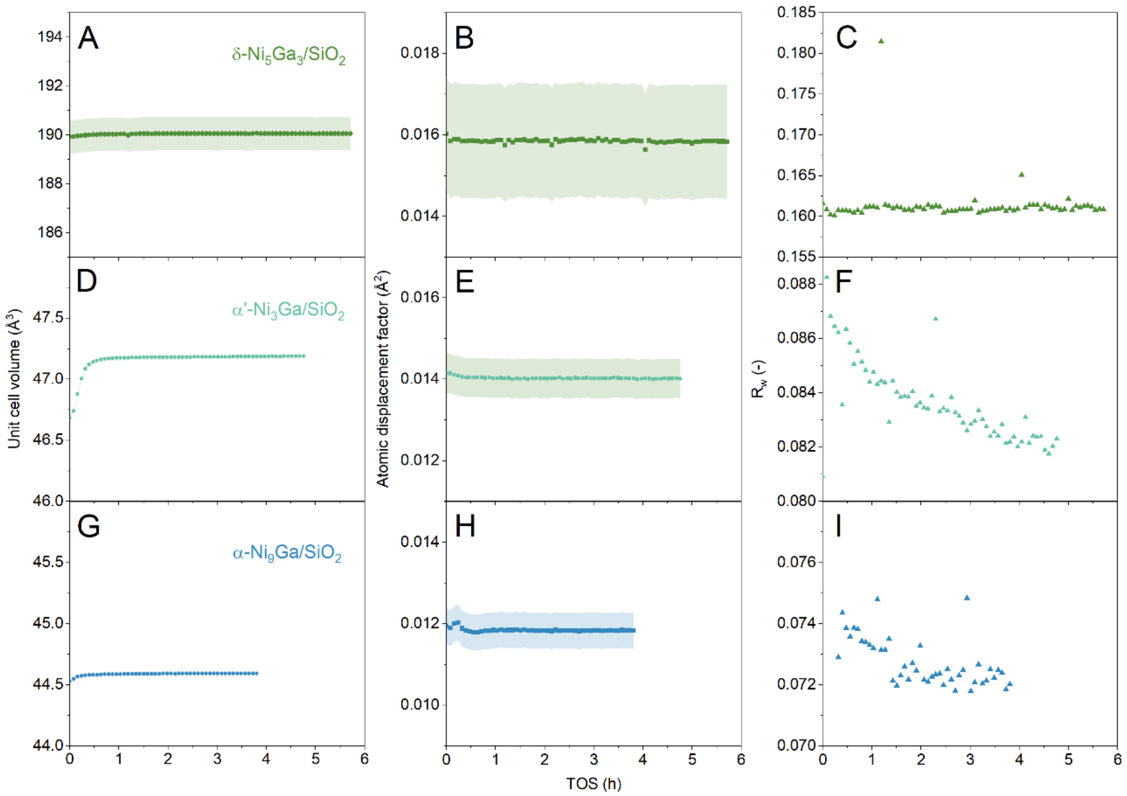

Figure S 10. Fitted lattice parameters, atomic displacement factors, and R_w extracted from the d-PDF of (A-C) δ-Ni_5Ga_3/SiO_2, (D-F) α'-Ni_3Ga/SiO_2 and (G-I) α-N_9Ga/SiO_2. The corresponding d-PDF data is shown in Figure S8.

Table S 3. Crystal structure refinement of δ-Ni_5Ga_3 obtained via fitting of the d-PDF of in-situ activated δ-Ni_5Ga_3/SiO_2. Data collected at 230 °C in 20 bar N_2.

Atom	Site	δ-Ni_5Ga_3			
		Site occ.	X	Y	Z
Ni(1)	*4j*	1.0	0	0.223(4)	0.5
Ni(2)	*4e*	1.0	0.25	0.25	0
Ni(3)	*2b*	1.0	0.5	0	0
Ga(1)	*2a*	1.0	0	0	0
Ga(2)	*4h*	1.0	0.278(4)	0	0.5
Lattice parameters, a, b, c, Å		7.42(2), 6.79(1), 3.7(4)			
Isotropic U factor, Å^2		0.0160(2)			
Atomic motion correlation factor, Å^2		3.8(4)			
Coherent particle size, Å		64(2)			
R_w, -		0.1616			

Table S 4. Crystal structure refinement of α'-Ni_3Ga obtained via fitting of the d-PDF of in-situ activated α'-Ni_3Ga/SiO_2. Data collected at 230 °C in 20 bar N_2.

Atom	Site	α'-Ni_3Ga			
		Site occ.	X	Y	Z
Ga	*1a*	1.0	0	0	0
Ni	*3c*	1.0	0	0.5	0.5
Lattice parameters, a, b, c, Å		3.6006(9), 3.6006(9), 3.6006(9)			
Isotropic U factor, Å^2		0.0142(5)			
Atomic motion correlation factor, Å^2		3.0(3)			
Coherent particle size, Å		65(1)			
R_w, -		0.0809			

Table S 5. Crystal structure refinement of α-Ni_9Ga obtained via fitting of the d-PDF of in-situ activated α-Ni_9Ga/SiO_2. Data collected at 230 °C in 20 bar N_2.

Atom	Site	α-Ni_9Ga			
		Site occ.	X	Y	Z
Ga	*4a*	0.1	0	0	0
Ni	*4a*	0.9	0	0	0
Lattice parameters, a, b, c, Å		3.5445(8), 3.5445(8), 3.5445(8)			
Isotropic U factor, Å^2		0.0119(4)			
Atomic motion correlation factor, Å^2		2.8(3)			
Coherent particle size, Å		62(1)			
R_w, -		0.0729			

Table S 6. Fitted lattice parameters extracted from the d-PDF of α'-Ni_3Ga/SiO_2, δ-Ni_5Ga_3/SiO_2, and α-Ni_9Ga/SiO_2 at different stages during the operando experiment.

	In situ activated (230 °C, 1 bar H_2)	In situ activated (230 °C, 20 bar N_2)	After 5 min TOS (230 °C, 20 bar CO_2:H_2:N_2 = 1:3:1)	After 1 h TOS (230 °C, 20 bar CO_2:H_2:N_2 = 1:3:1)	After 3.5 h TOS (230 °C, 20 bar CO_2:H_2:N_2 = 1:3:1)
**α-Ni_3Ga/SiO_2**					
Lattice parameter a=b=c, Å	3.6071(8)	3.6006(9)	3.6022(8)	3.6133(8)	3.61363(8)
Unit cell volume, Å^3	46.93(2)	46.68(2)	46.74(2)	47.17(2)	47.19(2)
**δ-Ni_5Ga_3/SiO_2**					
Lattice parameter a/b/c, Å	7.44(2)/6.771(9) /3.774(7)	7.42(2)/6.80(1) /3.768(7)	7.44(2)/6.78(1) /3.77(1)	7.44(2)/6.79(1) /3.768(9)	7.44(2)/6.79(1) /3.77(1)
Unit cell volume, Å^3	190.2(6)	189.9(6)	190.2(8)	190.3(7)	190.3(8)
**α-Ni_9Ga/SiO_2**					
Lattice parameter a=b=c, Å	3.4355(8)	3.5445(8)	3.5449(7))	3.5460(8)	3.5461(9)
Unit cell volume, Å^3	44.57(2)	44.53(2)	44.55(2)	44.59(2)	44.59(2)

4. Operando XAS experiments

Two macros were used for collecting XAS data. The data collected during the in situ activation step (heating up from room temperature to 700°C in 1 bar H_2, see Figure S5) were collected using the XANES macro, whereas the EXAFS macro was used for isothermal measurements.

XANES macro:

1. Ni XANES, 8250-8550 eV, ΔE = 0.3 eV, 50 seconds/spectrum
2. Ga XANES, 10300-10600 eV, ΔE = 0.3 eV, 50 seconds/spectrum

EXAFS macro:

1. Ni EXAFS, 8200-8970 eV, ΔE = 0.5 eV, 3 minutes/spectrum
2. Ga EXAFS, 10200-11100 eV, ΔE = 0.5 eV, 3 minutes/spectrum

XAS data analysis

EXAFS fittings of the k^2 weighed EXAFS were performed using the Artemis/Demeter v 0.9.26 software.[1] The amplitude reduction factors (S_0^2) were determined from reference materials (Ni K-edge: 0.82, determined from Ni-foil, Ga-edge: 0.92, determined from β-Ga_2O_3 and α'-Ni_3Ga), as reported elsewhere.[2] The fitted k values were in the range 3 - 11 $Å^{-1}$ (dk = 1 $Å^{-1}$), whereas the fitted r-range extended from 1-3 Å (dr = 0.5 Å) for both the Ni and Ga K-edge EXAFS. In the EXAFS fittings, a Ga-O sphere, as well as the first metal-metal sphere (Ni-M and Ga-M, with M = Ni/Ga) was included. Due to Ni and Ga having similar atomic numbers, we could not distinguish between Ni and Ga backscattering from EXAFS.

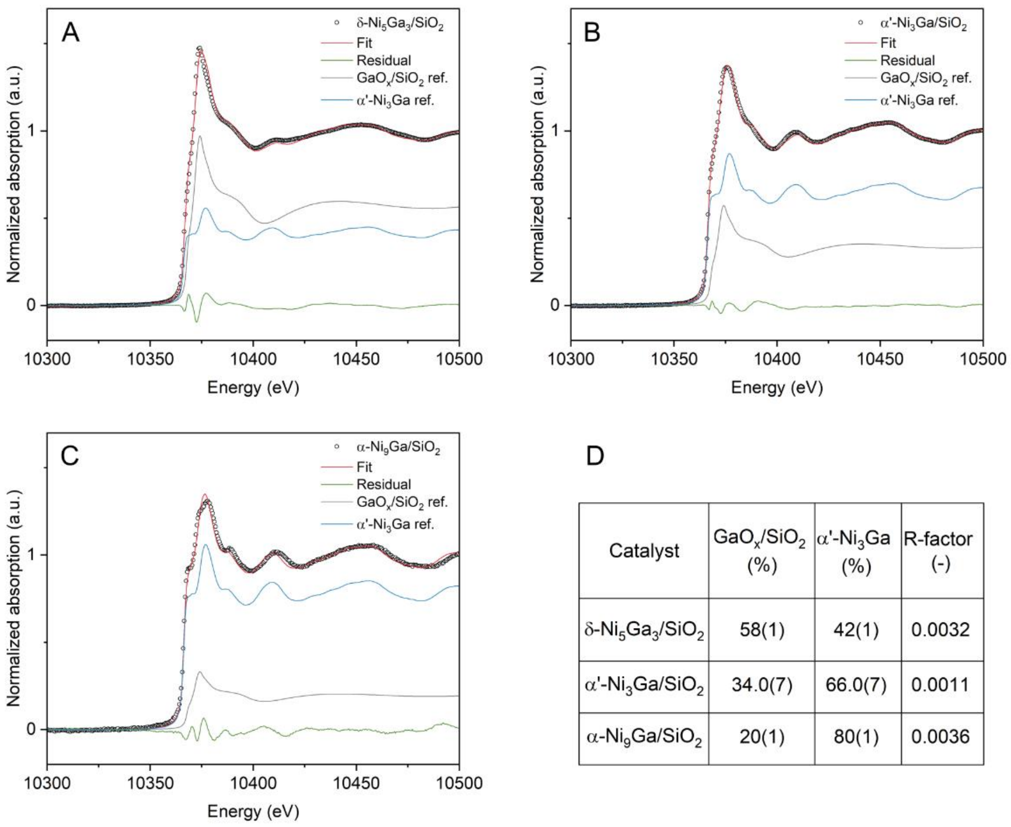

Catalyst	GaO_x/SiO_2 (%)	α'-Ni_3Ga (%)	R-factor (-)
δ-Ni_5Ga_3/SiO_2	58(1)	42(1)	0.0032
α'-Ni_3Ga/SiO_2	34.0(7)	66.0(7)	0.0011
α-Ni_9Ga/SiO_2	20(1)	80(1)	0.0036

Figure S 11. (A-C) Linear combination fittings of the Ga K-edge XANES using GaO_x/SiO_2 and α'-Ni_3Ga as references for GaO_x and alloyed Ga, respectively. (D) Obtained LCF fractions.

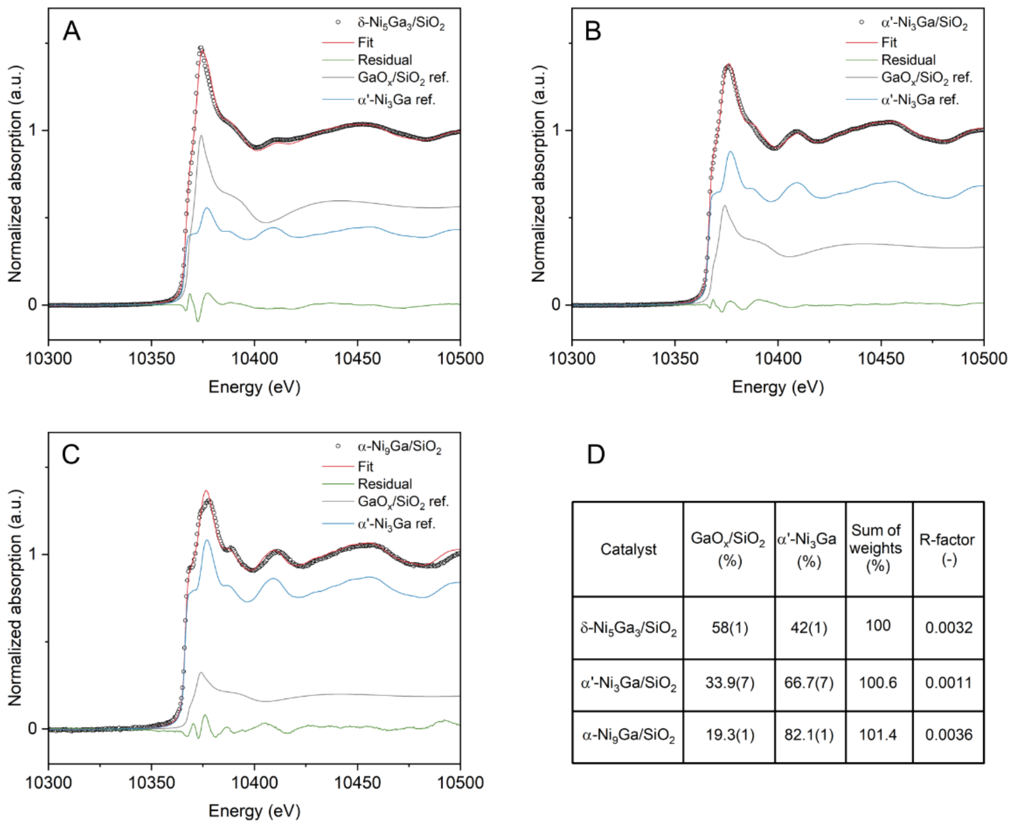

Catalyst	GaO_x/SiO_2 (%)	α'-Ni_3Ga (%)	Sum of weights (%)	R-factor (-)
δ-Ni_5Ga_3/SiO_2	58(1)	42(1)	100	0.0032
α'-Ni_3Ga/SiO_2	33.9(7)	66.7(7)	100.6	0.0011
α-Ni_9Ga/SiO_2	19.3(1)	82.1(1)	101.4	0.0036

Figure S 12. LCF fitting results without restraining the sum of the components to 100%. These results show that the component sum in each case is close to 100%, and the fitted LCF weights closely match those obtained under the explicit constraint of a fixed 100% sum (Figure S11). This finding supports that the two components chosen sufficiently account for the underlying spectral features.

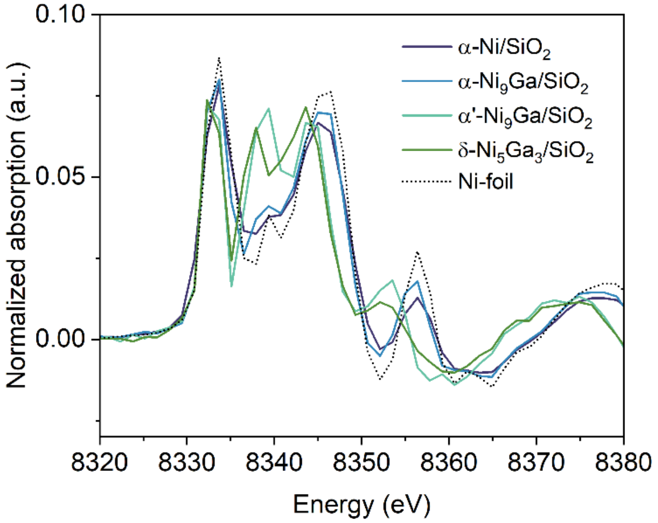

Figure S 13. First derivative of the Ni K-edge XANES shown in Figure 2A of the main text.

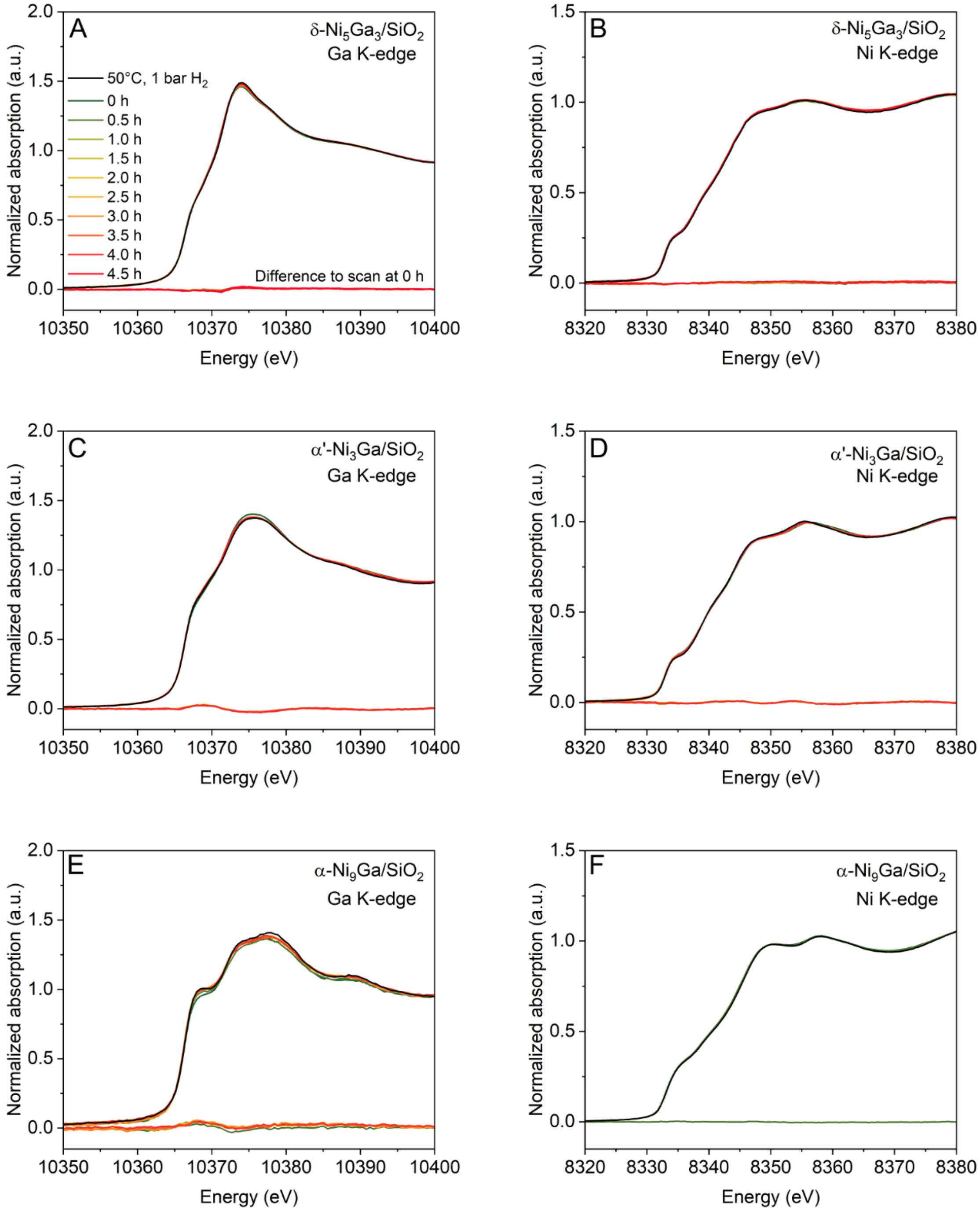

Figure S 14. Ga and Ni K-edges XANES under CO_2 hydrogenation conditions. (A-B) δ-Ni_5Ga_3/SiO_2. (C-D) α'-Ni_3Ga/SiO_2. (E- F) α-Ni_9Ga/SiO_2. Data collected at 230 °C in 20 bar $CO_2:H_2:N_2$ = 1:3:1.

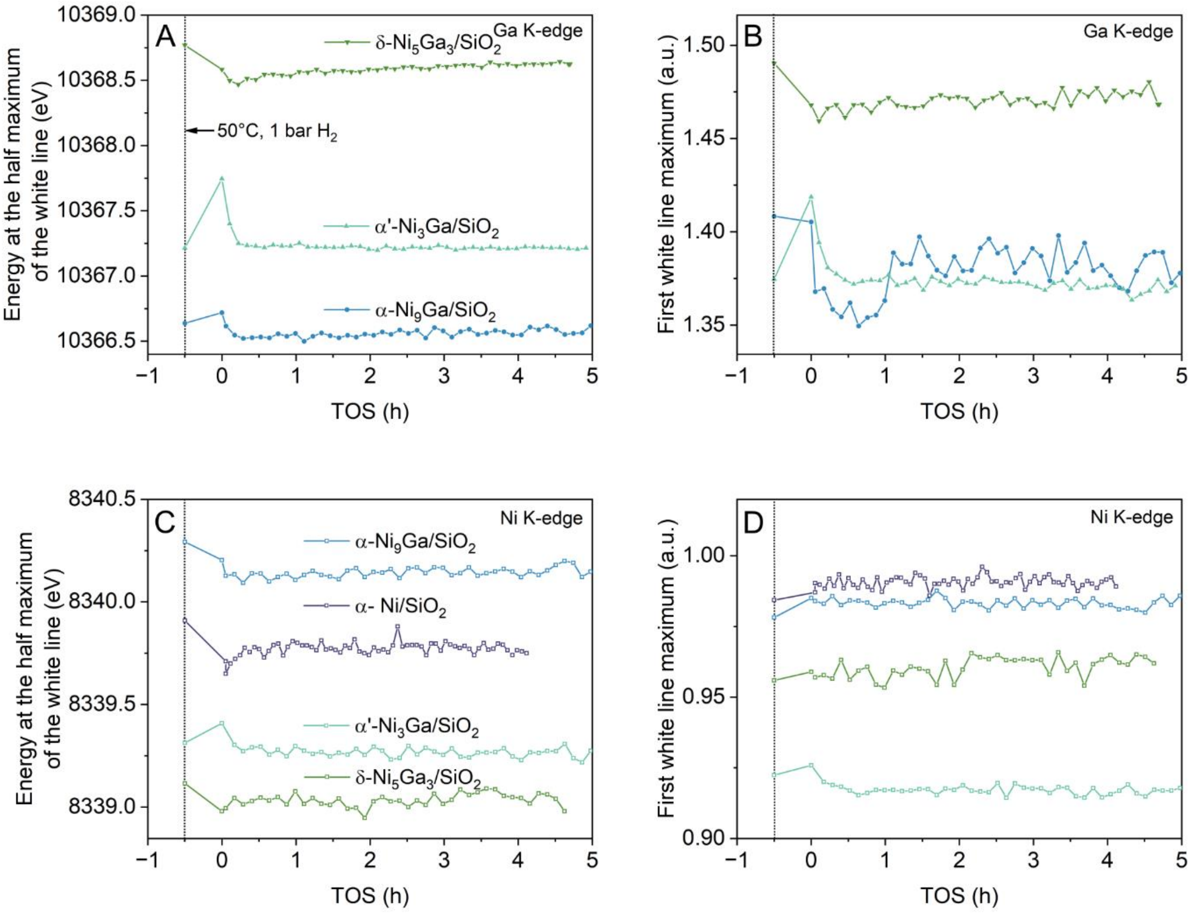

Figure S 15. Energy at the half maximum of the white line and absorption at the first white line maximum under CO_2 hydrogenation conditions. The points at TOS = 0 correspond to the state of the catalyst at 230 °C in 20 bar N_2, just before switching to the reaction atmosphere of CO_2:H_2:N_2=1:3:1. (A, B) Ga K-edge XANES. (C, D) Ni K-edge XANES.

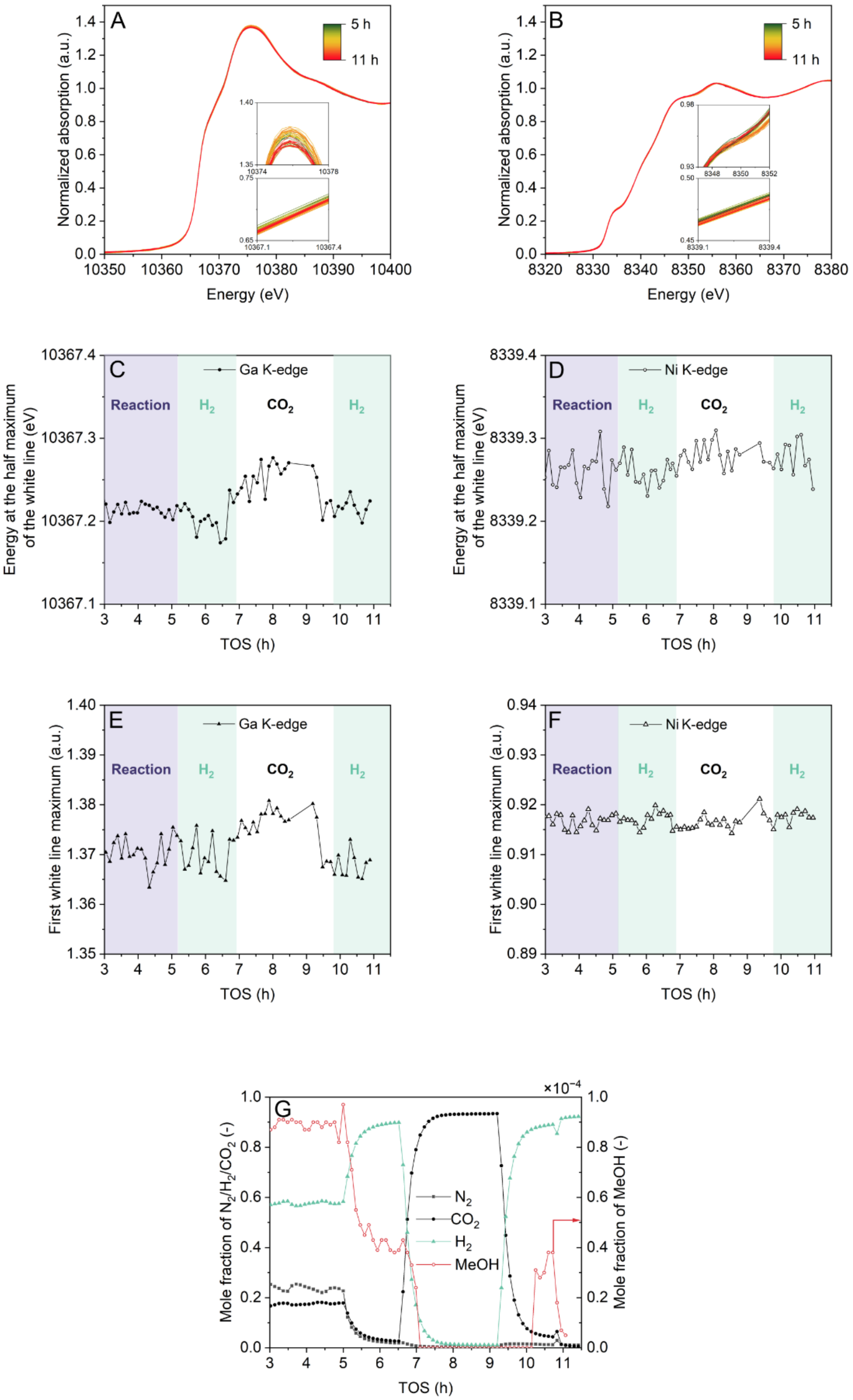

Figure S 16. XANES of α'-Ni_3Ga/SiO_2 collected during a H_2-CO_2-H_2 gas switching sequence (5 – 11 h TOS), following its exposure to CO_2 hydrogenation conditions (up to 5 h TOS): (A) Ga XANES (B) Ni XANES. Corresponding energy at the half maximum of the white line (C, D) and absorption at the first white line maximum (E, F). (G) Mole fractions of CO_2, H_2, and MeOH during the gas switching experiment.

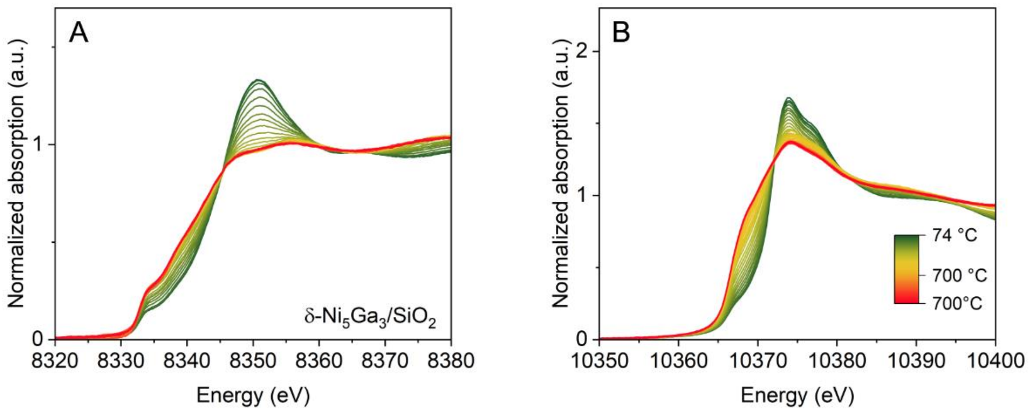

Figure S 17. (A) Ni and (B) Ga XANES of δ-Ni_5Ga_3/SiO_2 collected during the in situ activation treatment in H_2. It is important to note that these catalysts were pre- activated ex situ (700 °C, H_2, in a laboratory scale reactor) and subsequently exposed to air prior to the in situ XAS experiment.

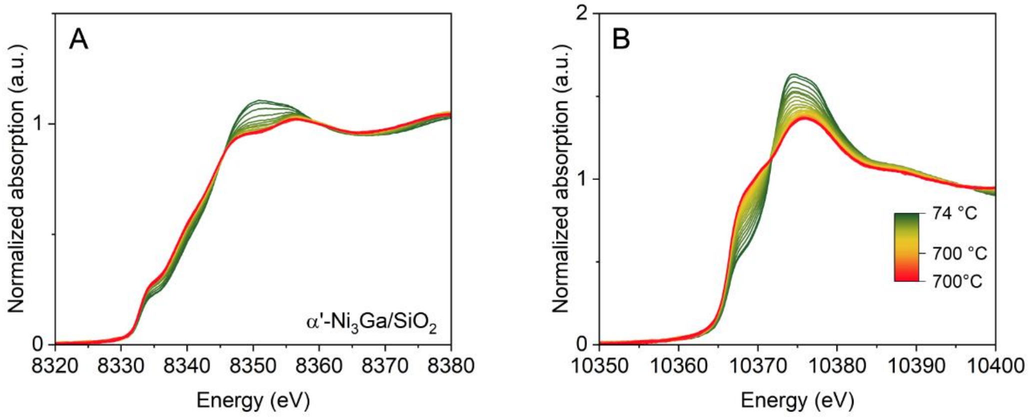

Figure S 18. (A) Ni and (B) Ga XANES of α'-Ni_3Ga/SiO_2 collected during the in-situ activation treatment in H_2. It is important to note that these catalysts were pre- activated ex situ (700 °C, H_2, in a laboratory scale reactor) and subsequently exposed to air prior to the in situ XAS experiment.

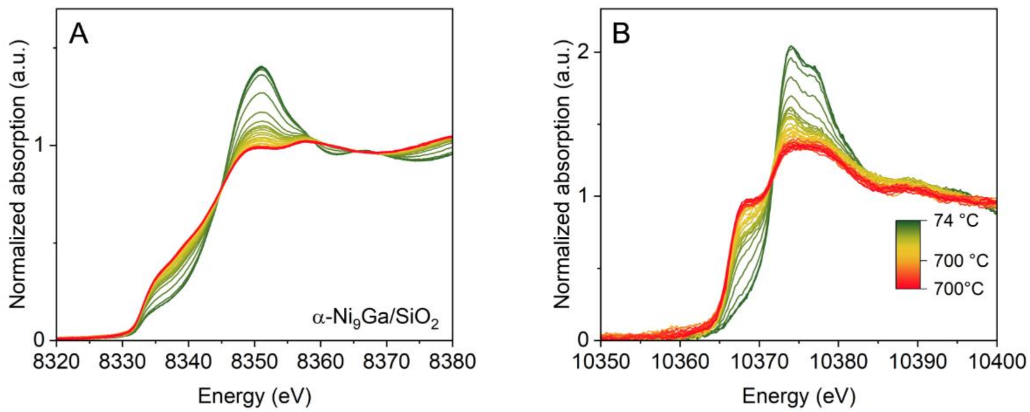

Figure S 19. (A) Ni and (B) Ga XANES of α-Ni_9Ga/SiO_2 collected during the in-situ activation treatment in H_2. It is important to note that these catalysts were pre- activated ex situ (700 °C, H_2, in a laboratory scale reactor) and subsequently exposed to air prior to the in situ XAS experiment.

Table S 7. Fitted EXAFS parameters. The errors on the reported values were estimated by the software Artemis are given in compact (crystallographic) notation, i.e., 2.52(1) corresponds to 2.52 ± 0.01. Parameters without errors were fixed. Abbreviations: Rw: weighted R factor, ΔE: the edge energy shift, CN: coordination number, σ: Debye- Waller factor, r: interatomic distance.

Material	Model phase	CN(Ga-O) (-)	σ(Ga-O) (Å²)	r(Ga-O) (Å)	CN(Ni-M) (-)	σ(Ni-M) (Å²)	r(Ni-M) (Å)	CN(Ga-M)	σ(Ga-M)	r(Ga-M)	ΔE_{Ni} (eV)	ΔE_{Ga} (eV)	Rw_{Ni} (-)	Rw_{Ga} (-)
δ-Ni_5Ga_3/SiO_2, activated	δ	1.7(3)	0.010(3)	1.84(2)	9(1)	0.009(1)	2.54(2)	5.3(5)	0.0099(9)	2.50(2)	4(1)	6(1)	0.0160	0.0043
δ-Ni_5Ga_3/SiO_2, reacted	δ	1.7(3)	0.008(3)	1.84(1)	8(1)	0.008(1)	2.54(2)	5.1(5)	0.0097(9)	2.50(3)	5(1)	6(1)	0.0118	0.0051
α'-Ni_3Ga/SiO_2, activated	α'	1.0(4)	0.007(7)	1.85(2)	9.8(7)	0.0079(7)	2.54(1)	8.8(9)	0.011(1)	2.54(1)	5.6(7)	7(1)	0.00458	0.0047
α'-Ni_3Ga/SiO_2, reacted	α'	1.0(3)	0.006(5)	1.84(2)	9.8(7)	0.0080(6)	2.54(1)	8.3(8)	0.0102(9)	2.544(9)	5.6(7)	7(1)	0.00341	0.0052
α-Ni_9Ga/SiO_2, activated	α	0.6(7)	0.00(1)	1.80(2)	10.6(5)	0.0067(4)	2.49(5)	8(2)	0.008(2)	2.51(3)	5.9(4)	6(2)	0.00234	0.0041
α-Ni_9Ga/SiO_2, reacted	α	2(2)	0.01(2)	1.85(3)	10.0(6)	0.0068(5)	2.48(5)	7(2)	0.008(3)	2.51(2)	6.0(6)	7(4)	0.0046	0.0076
α-Ni/SiO_2-XAS, activated	α	-	-	-	9.6(4)	0.0065(4)	2.48(5)	-	-	-	6.0(5)	.	0.0020	-

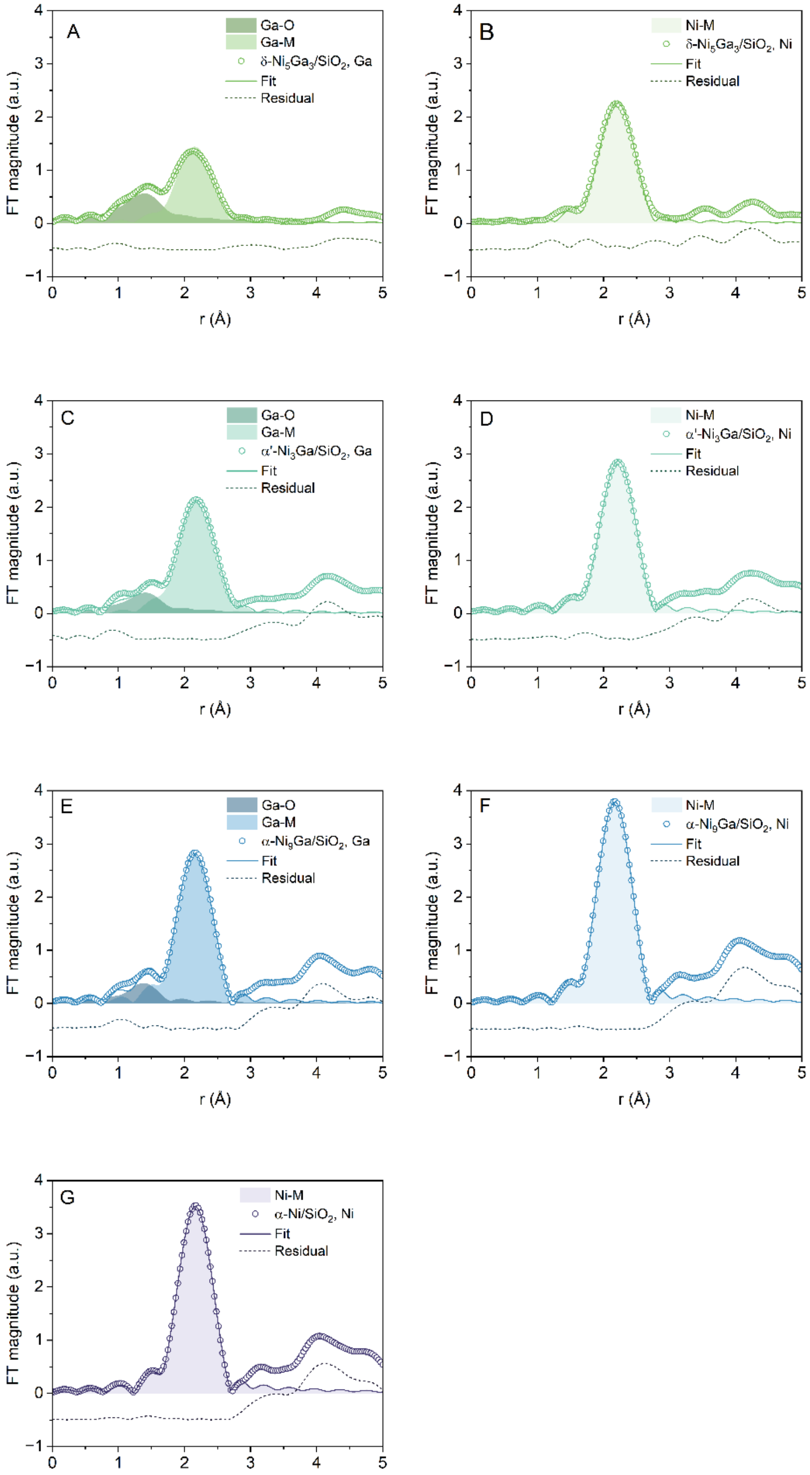

Figure S 20. Fitted k[2] weighed Ni and Ga K edge EXAFS of the in situ activated catalysts. (A, B) δ-Ni_5Ga_3/SiO_2. (C, D) α'-Ni_3Ga/SiO_2. (E, F) α-Ni_9Ga/SiO_2. (G) Ni/SiO_2. M = Ni/Ga in Ni-M and Ga-M. Data collected at 50 °C in 1 bar H_2.

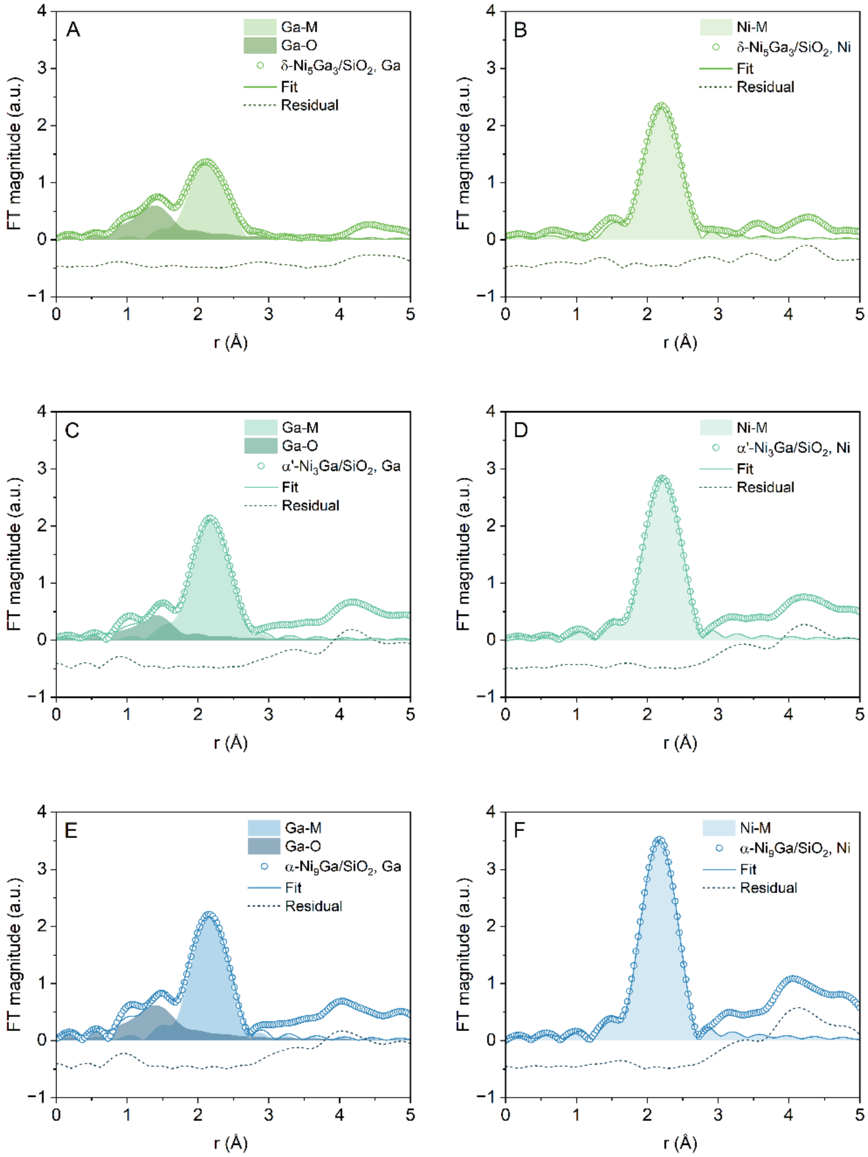

Figure S 21. Fitted k^2 weighed Ni and Ga K edge EXAFS of the reacted catalysts. (A, B) δ-Ni_5Ga_3/SiO_2. (C, D) α'-Ni_3Ga/SiO_2. (E, F) α-Ni_9Ga/SiO_2. M = Ni/Ga in Ni-M and Ga-M. Data collected at 50 °C in 1 bar H_2.

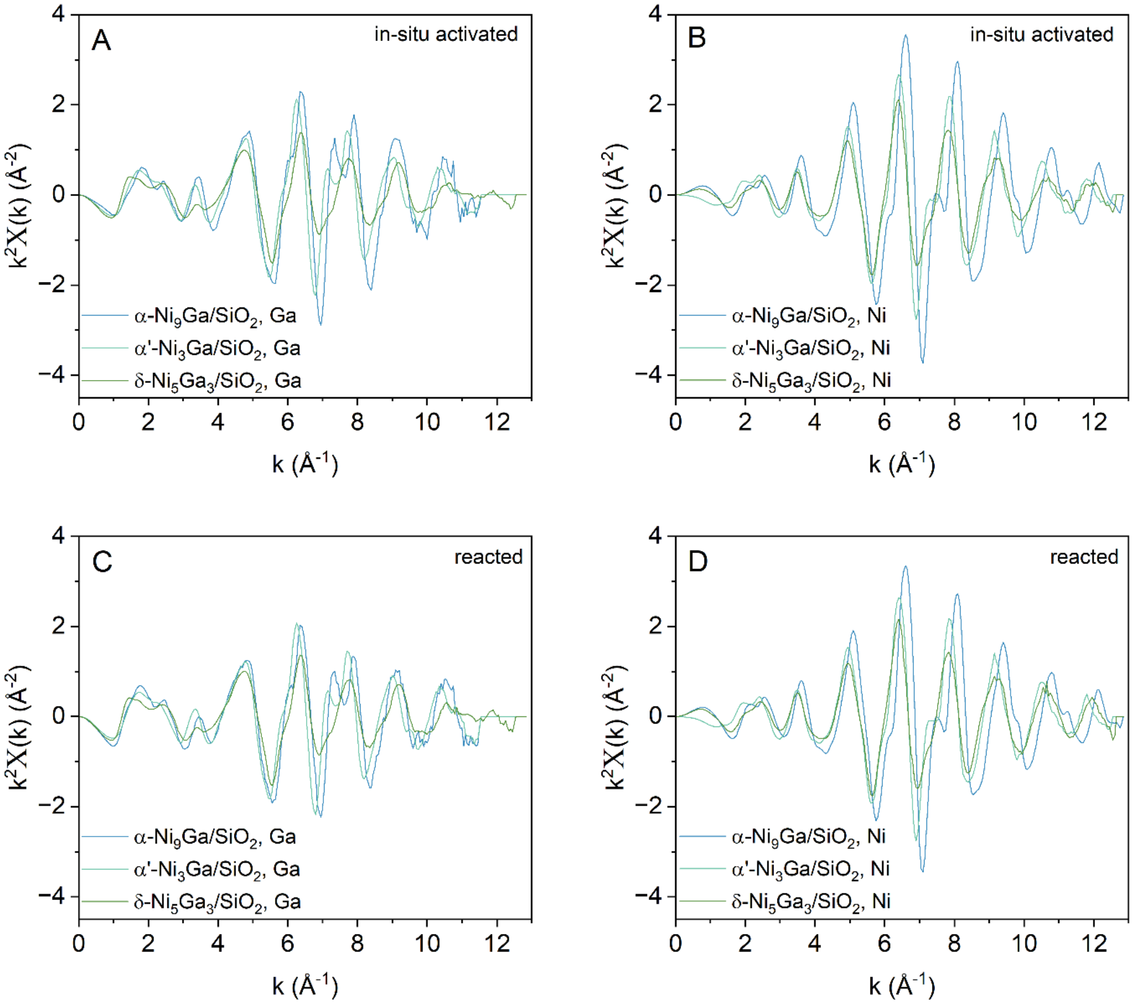

Figure S 22. k^2 weighed EXAFS data. (A, B) In situ activated catalysts. (C, D) Reacted catalysts.

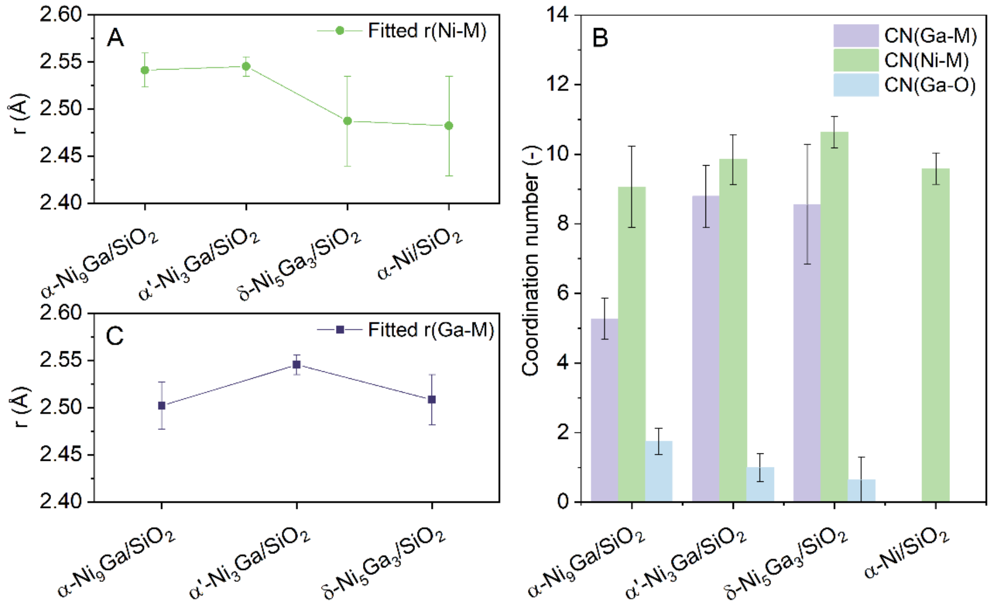

Figure S 23. (A, C) Fitted Ni/Ga-M distances obtained from EXAFS modeling of the XAS data collected on the in situ activated catalysts. (B) Fitted coordination numbers for Ga-M, Ni-M, and Ga-O. This data was collected at 50 °C in 1 bar H_2.

5. CO_2 hydrogenation tests

The calculation of the catalytic performance parameters was done as follows:

Total outgas flow rate (mol min^{-1}):

$$F_{tot,out} = \frac{F_{tot,in} \times C_{N2,in}}{C_{N2,out}}$$

Mass formation rate of product i per total catalyst mass ($g_i\ g_{cat}^{-1}\ h^{-1}$):

$$r_{i,gcat} = \frac{C_i \times F_{tot,out} \times MW_i \times 60\ (\frac{min}{h})}{m_{cat}}$$

Molar formation rate of product i per mol of Ni in the catalyst ($mmol_i\ mol_{Ni}^{-1}\ s^{-1}$):

$$r_{i,Ni} = \frac{C_i \times F_{tot,out} \times 10^3 \left(\frac{mmol}{mol}\right) \times MW_{Ni}}{m_{cat} \times w_{Ni} \times 60\ (\frac{s}{min})}$$

Molar formation rate of product i per mol of (Ni+Ga) in the catalyst ($mmol_i\ mol_{Ni+Ga}^{-1}\ s^{-1}$):

$$r_{i,Ni} = \frac{C_i \times F_{tot,out} \times 10^3 \left(\frac{mmol}{mol}\right)}{m_{cat} \times \left(\frac{w_{Ni}}{MW_{Ni}} + \frac{w_{Ga}}{MW_{Ga}}\right) \times 60\ (\frac{s}{min})}$$

Selectivity of product i (%): $S_i = \frac{C_i}{C_{CH3OH} + C_{CO} + C_{CH4}} \times 100\ \%$

CO_2 conversion (%): $X_{CO2} = \frac{F_{tot,out} \times (C_{CH3OH} + C_{CO} + C_{CH4})}{F_{tot,in} \times C_{CO2,in}} \times 100\ \%$

where $\boldsymbol{F_{tot,in}}$ **(mol min^{-1})** is the total gas flow rate at the inlet of the reactor, $\boldsymbol{C_{N2,in}}$ **(-)** is the concentration of N_2 in the inlet gas, $\boldsymbol{C_{N2,out}}$ **(-)** is the concentration of N_2 in the off-gas, $\boldsymbol{C_i}$ **(-)** is the concentration of product i ∈{CH_3OH, CO, CH_4} in the off-gas, **MW_i (g mol^{-1})** is the molecular weight of species i, m_{cat} (g) is the catalyst mass, $\boldsymbol{w_{Ni}}$ (-) and $\boldsymbol{w_{Ga}}$ (-) are the weight fractions of Ni and Ga in the catalyst as measured by ICP-OES, and $\boldsymbol{C_{CO2,in}}$ **(-)** is the concentration of CO_2 in the inlet gas.

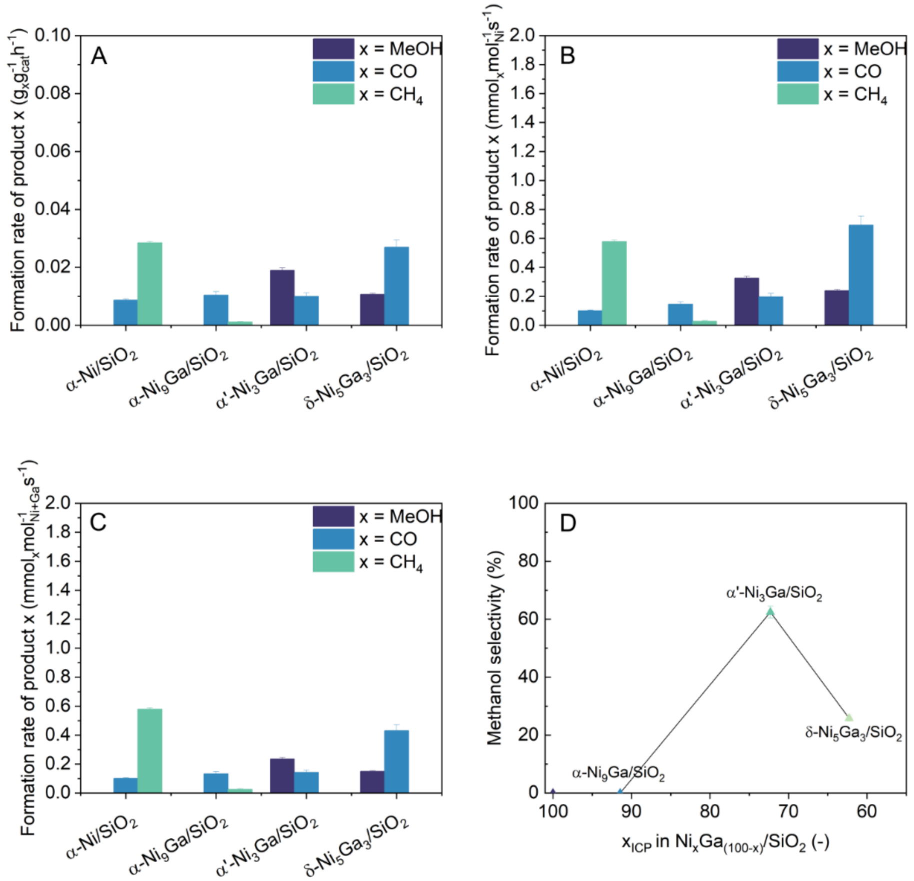

Figure S 24. Summary of catalytic data at 1 bar, average of 6 GC points. (A) Product formation rates normalized by gram of catalyst. (B) Product formation rates normalized by the Ni content of the catalysts. (C) Product formation rates normalized by the total metal content of the catalysts. (D) Methanol selectivity as a function of the Ni content of the catalysts.

Table S 8. Product formation rates, selectivities and CO_2 conversions measured at 1 and 25 bar reaction pressure.

**Catalyst**	**MeOH formation rate ($mmol_{MeOH}\ mol_{Ni+Ga}^{-1}\ s^{-1}$/ $mmol_{MeOH}\ mol_{Ni}^{-1}\ s^{-1}$/ $10^{-2}*g_{MeOH}\ g_{cat}^{-1}\ h^{-1}$)**		**CO formation rate ($mmol_{CO}\ mol_{Ni+Ga}^{-1}\ s^{-1}$/ $mmol_{CO}\ mol_{Ni}^{-1}\ s^{-1}$/ $10^{-2}*g_{CO}\ g_{cat}^{-1}\ h^{-1}$)**		**CH_4 formation rate ($mmol_{CH4}\ mol_{Ni+Ga}^{-1}\ s^{-1}$/ $mmol_{CH4}\ mol_{Ni}^{-1}\ s^{-1}$/ $10^{-2}*g_{CH4}\ g_{cat}^{-1}\ h^{-1}$)**		**MeOH selectivity (%)**		**CO selectivity (%)**		**CH_4 selectivity (%)**		**CO_2 conversion (%)**	
Reaction pressure (bar)	1	25	1	25	1	25	1	25	1	25	1	25	1	25
α-Ni/SiO_2	0/0/0	0.034(1)/ 0.034(1)/ 0.33(1)	0.101(5)/ 0.101(5)/ 0.87(4)	0.042(2)/ 0.042(2)/ 0.36(2)	0.58(1)/ 0.58(1)/ 2.84(5)	1.66(3)/ 1.66(3)/ 8.2(2)	0	1.94(9)	14.8(5)	2.41(9)	85.2(5)	95.65(9)	0.431(9)	1.10(2)
α-Ni_9Ga/SiO_2	0/0/0	0.033(3)/ 0.037(3)/ 0.30(3)	0.13(2)/ 0.14(2)/ 1.0(1)	0.138(5)/ 0.151(6)/ 1.08(4)	0.025(5)/ 0.028(5)/ 0.11(2)	0.138(4)/ 0.150(5)/ 0.61(2)	0	10.7(7)	84(1)	44.8(6)	16(1)	44.5(6)	0.09(1)	0.178(7)
α'-Ni_3Ga/SiO_2	0.235(1)/ 0.33(1)/ 1.90(8)	0.55(2)/ 0.76(3)/ 4.4(2)	0.14(2)/ 0.20(2)/ 1.0(1)	0.22(2)/ 0.30(2)/ 1.54(9)	0/0/0	0/0/0	62(2)	71.4(5)	37(2)	28.6(5)	0	0	0.20(1)	0.40(2)
δ-Ni_5Ga_3/SiO_2	0.149(5)/ 0.239(9)/ 1.07(4)	0.49(1)/ 0.79(2)/ 3.51(9)	0.43(4)/ 0.69(6)/ 2.7(2)	0.40(2)/ 0.65(2)/ 2.5(1)	0/0/0	0/0/0	26(1)	54.9(5)	74(1)	45.1(5)	0	0	0.27(2)	0.41(1)

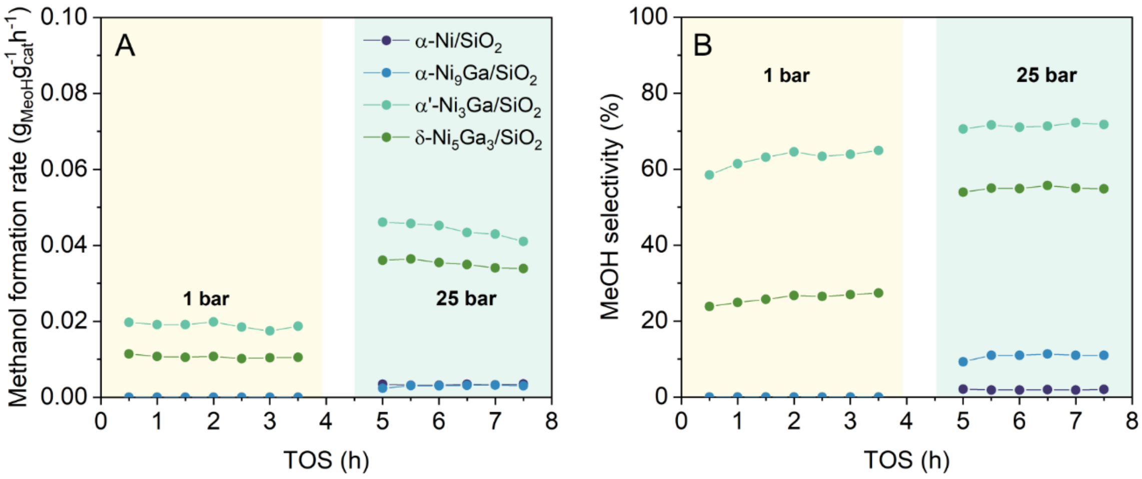

Figure S 25. (A) Methanol formation rate. (B) Methanol selectivity. Conditions: 230 °C, 1/25 bar CO_2:H_2:N_2 = 1:3:1, GHSV = 60 L gcat^{-1} h^{-1}.

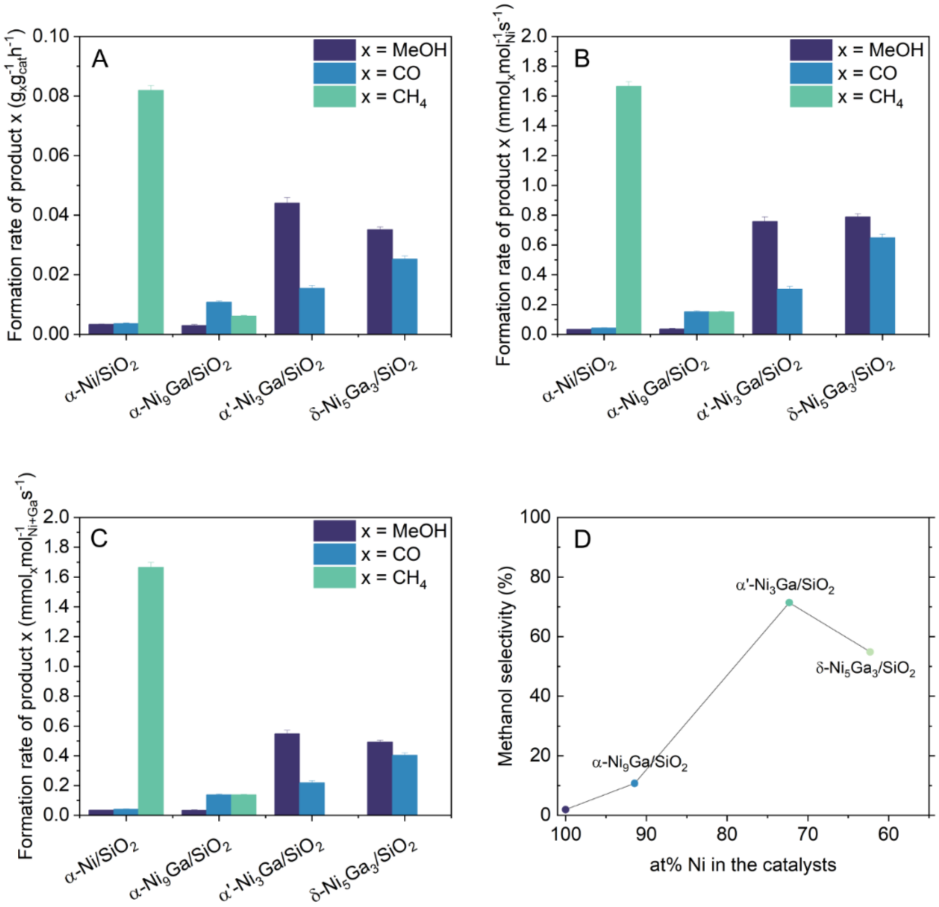

Figure S 26. Summary of catalytic data at 25 bar, average of 6 GC points. (A) Product formation rates normalized by gram of catalyst. (B) Product formation rates normalized by the Ni content of the catalysts. (C) Product formation rates normalized by the total metal content of the catalysts. (D) Methanol selectivity as a function of the Ni content of the catalysts.

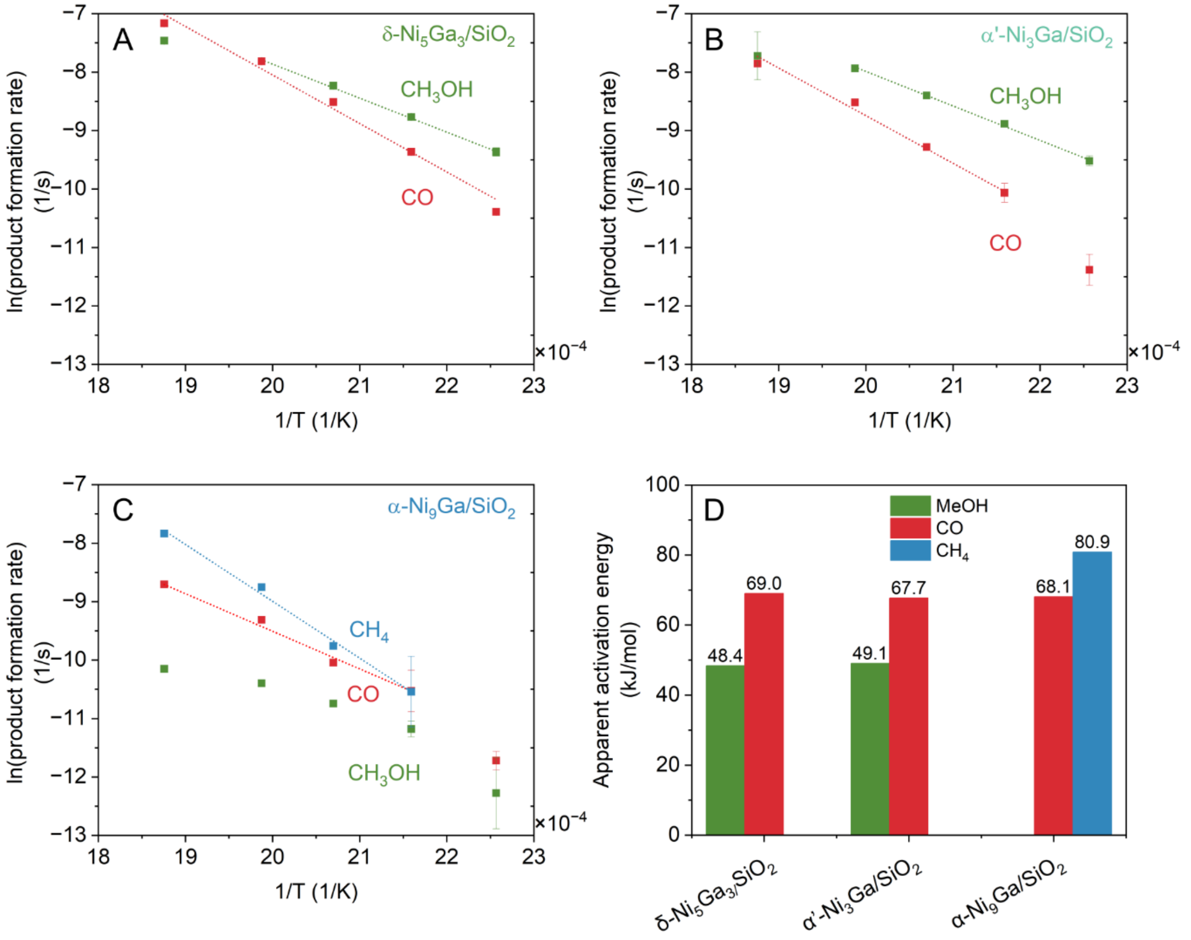

Figure S 27. (A-C) Arrhenius plots used to calculate the apparent activation energies for CO_2 hydrogenation to methanol (green), for the RWGS reaction (red), and CO_2 methanation (blue). Measured data points, as well as linear regression curves (dotted lines) are shown. (D) Apparent activation energies.

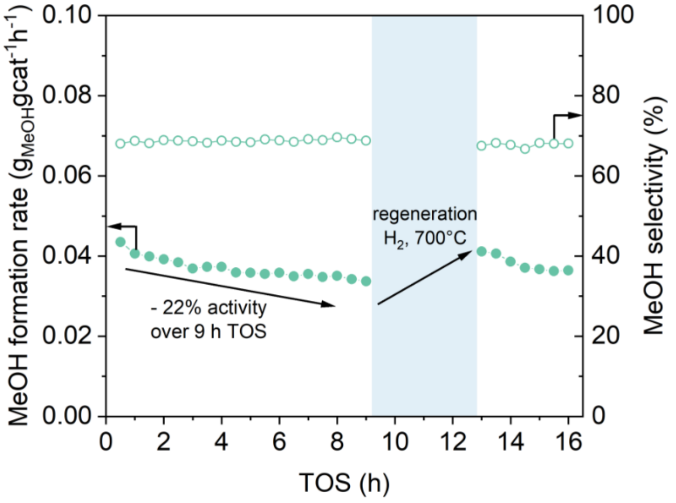

Figure S 28. Stability tests with α'-Ni_3Ga/SiO_2. Catalyst amount – 100 mg; activation treatment – 1 hour at 700 °C in H_2; reaction conditions – 25 bar with a gas mixture of CO_2:H_2:N_2 = 1:3:1 at a total flow rate of 100 mL/min (GHSV = 60 ml gcat^{-1} h^{1-}). The catalyst was held under reaction conditions for 9 hours, followed by catalyst regeneration via heating at 700 °C under 1 bar H_2 (50 ml/min). The reaction was then resumed for an additional 3 hours.

6. DRIFTS

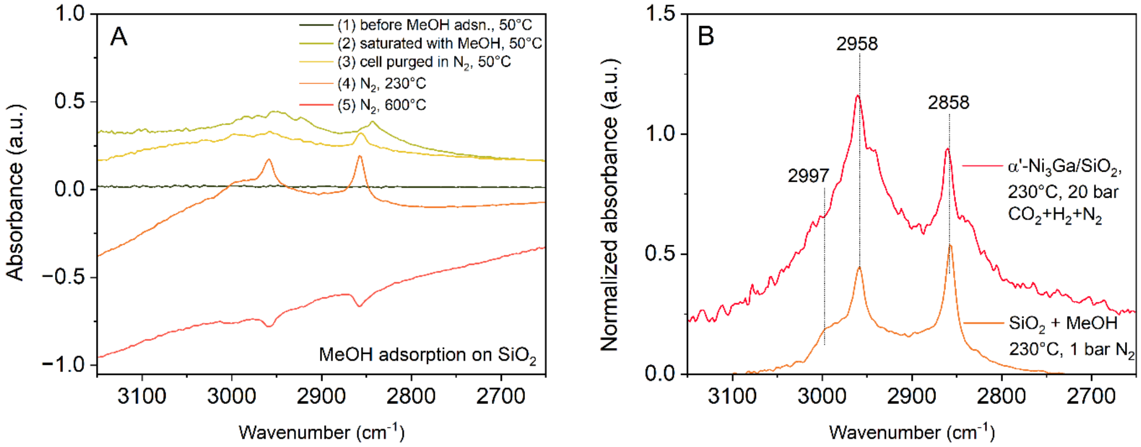

Figure S 29. MeOH adsorption experiment on pristine SiO_2 performed at 1 bar N_2 and using a bubbler to saturate the sample cell with methanol vapor. (A) DRIFT spectra obtained at different experiment stages: 1) Before MeOH adsorption at 50 °C in N_2. 2) Surface is saturated with MeOH vapor, 50 °C. 3) After purging the cell for 30 minutes in N_2 at 50°C. 4) After heating to 230°C in N_2. 5) After heating to 600 °C in N_2. (B) Comparison of the DRIFT spectra of MeOH adsorbed on SiO_2 at 230 °C and α'-Ni_3Ga/SiO_2 under reaction conditions (230 °C, 20 bar CO_2:H_2:N_2 = 1:3:1).

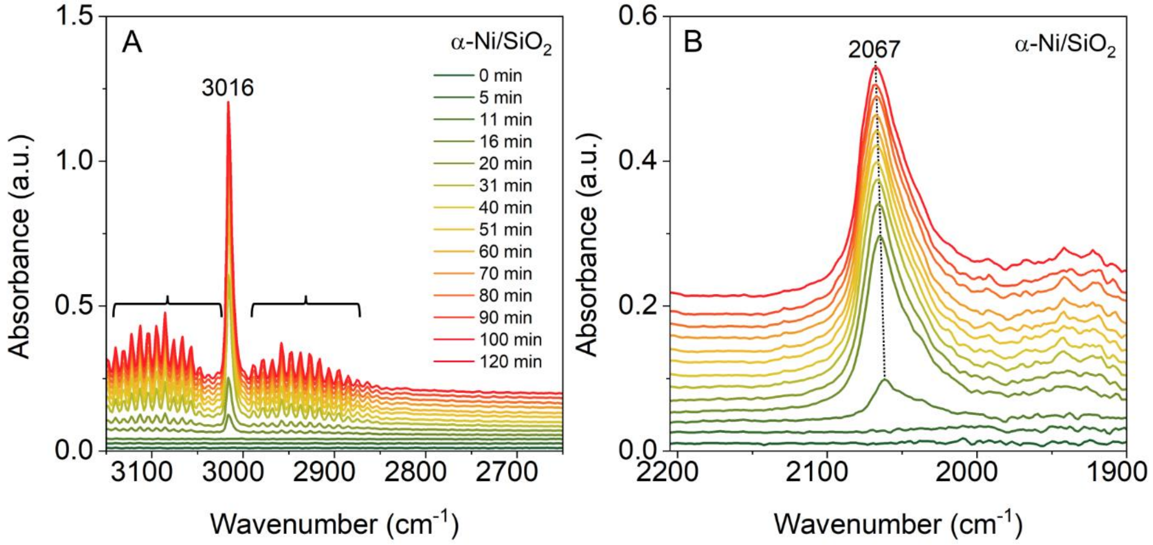

Figure S 30. DRIFTS data collected on α'-Ni_3Ga at 230 °C after switching the gases from 20 bar N_2 (= 0 min) to 20 bar CO_2:H_2:N_2 = 1:3:1. (A) The fundamental ν(C-H) of gaseous methane is observed at 3016 cm^{-1}, whereas strong bands due to rotational transitions of CH_4(g) (curly brackets) can also be observed. (B) The band observed at 2067 cm^{-1} is assigned to CO adsorbed on Ni.

Table S 9. DRIFTS band assignments.

Wavenumber (cm^{-1})	Experiment observed	Band assignment	Vibrational mode	Reference
3650 – 3760	α'-Ni_3Ga/SiO_2, α-Ni/SiO_2	CO_2 (g)	Combination, $\nu_1 + \nu_3$	[3]
3500 – 3650	α'-Ni_3Ga/SiO_2, α-Ni/SiO_2	CO_2 (g)	Combination, $2\nu_2 + \nu_3$	[3]
3016	α-Ni/SiO_2	CH_4(g)	ν_1(C-H)	[4]
2860, 2960	α'-Ni_3Ga/SiO_2	-OCH_3 on SiO_2	ν_{as}(C-H)	[5,6]
2200 – 2450	α'-Ni_3Ga/SiO_2, α-Ni/SiO_2	CO_2 (g)	Fundamental stretching, ν_3	[3]
2165, 2130, 2094, 2077	α'-Ni_3Ga/SiO_2	CO_2 (g)	Combination band	[3]
2051, 2061, 2067	α'-Ni_3Ga/SiO_2, α-Ni/SiO_2	Adsorbed CO_n on Ni (n= 1-4) and gas phase $Ni(CO)_4$	ν(C-O)	[7–10]

7. Density functional theory calculations

Reaction pathways

CO formation pathway

$$CO_{2(g)} + 3H_{2(g)} \rightarrow CO^* + O^* + 3H_{2(g)}$$
$$CO_{2(g)} + 3H_{2(g)} \rightarrow CO^* + H_2O_{(g)} + 2H_{2(g)}$$
$$CO_{2(g)} + 3H_{2(g)} \rightarrow CO_{(g)} + H_2O_{(g)} + 2H_{2(g)}$$

CH_4 formation pathway

$$CO_{2(g)} + 3H_{2(g)} \rightarrow CO^* + O^* + 3H_{2(g)}$$
$$CO_{2(g)} + 3H_{2(g)} \rightarrow C^* + 2O^* + 3H_{2(g)}$$
$$CO_{2(g)} + 3H_{2(g)} \rightarrow C^* + 2H_2O_{(g)} + H_{2(g)}$$
$$CO_{2(g)} + 3H_{2(g)} \rightarrow CH_{4(g)} + 2H_2O_{(g)} - H_{2(g)}$$

CH_3OH formation pathway from $HCOO^*$

$$CO_{2(g)} + 3H_{2(g)} \rightarrow HCOO^* + \frac{5}{2}H_{2(g)}$$
$$CO_{2(g)} + 3H_{2(g)} \rightarrow CH_3O^* + O^* + \frac{3}{2}H_{2(g)}$$
$$CO_{2(g)} + 3H_{2(g)} \rightarrow CH_3O^* + H_2O_{(g)} + \frac{1}{2}H_{2(g)}$$
$$CO_{2(g)} + 3H_{2(g)} \rightarrow CH_3OH_{(g)} + H_2O_{(g)}$$

CH_3OH formation pathway from CO^*

$$CO_{2(g)} + 3H_{2(g)} \rightarrow CO^* + O^* + 3H_{2(g)}$$
$$CO_{2(g)} + 3H_{2(g)} \rightarrow CH_3O^* + O^* + \frac{3}{2}H_{2(g)}$$

$$CO_{2(g)} + 3H_{2(g)} \rightarrow CH_3O^* + H_2O_{(g)} + \frac{1}{2}H_{2(g)}$$
$$CO_{2(g)} + 3H_{2(g)} \rightarrow CH_3OH_{(g)} + H_2O_{(g)}$$

Transition states

$$CO_2^* + H^* \rightarrow HCOO^* \; (TS1 = H - COO^‡)$$

$$HCOOH^* + H^* \rightarrow H_2COOH^* \; (TS2 = H - HCOOH^‡)$$

Models

Table S 10. Lattice parameters obtained by DFT calculations. The atomic positions for α'-Ni_3Ga, δ-Ni_5Ga_3, and α-Ni, are the same as indicated in Tables S3-S5.

System	Crystal system	Space group	a [Å]	b [Å]	c [Å]	α=β=γ
**α-Ni**	Cubic	Fm-3m	3.5180	3.5180	3.5180	90°
**α'-Ni_3Ga**	Cubic	Pm-3m	3.5870	3.5870	3.5870	90°
**δ-Ni_5Ga_3**	Orthorhombic	Cmmm	7.5407	6.7897	3.7450	90°

Table S 11. *k*-mesh grid and size of supercell employed for the modeling of each surface.

System	K-mesh grid	supercell	Side view
α-Ni(111)	8x8x1	2x2x1	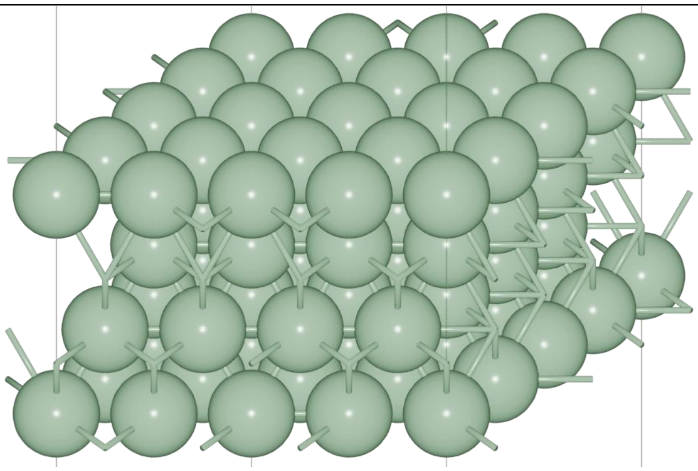

α-Ni(211)	8x4x1	2x3x1	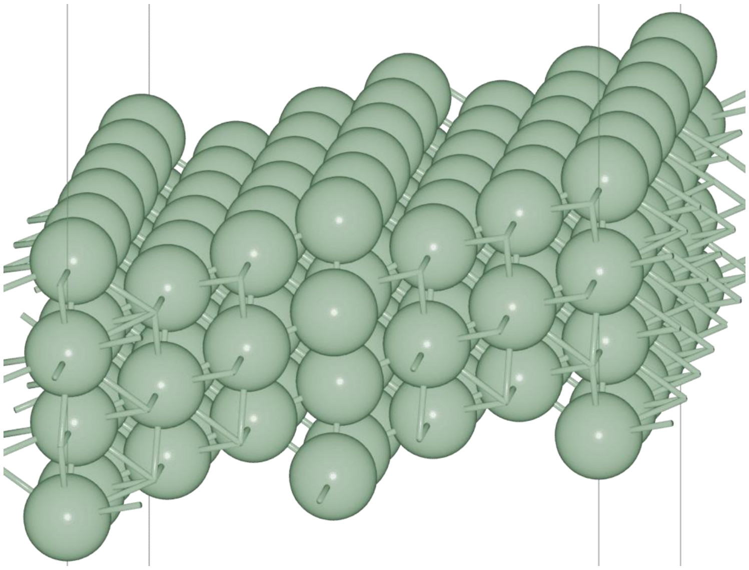
α'-Ni_3Ga(111)	8x8x1	2x2x1	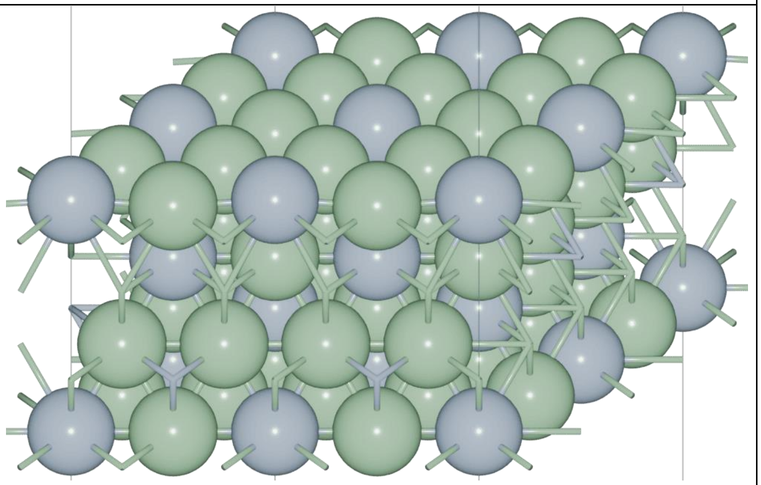
α'-$Ni_3Ga(211)_{Ni\text{-}step}$	4x4x1	2x3x1	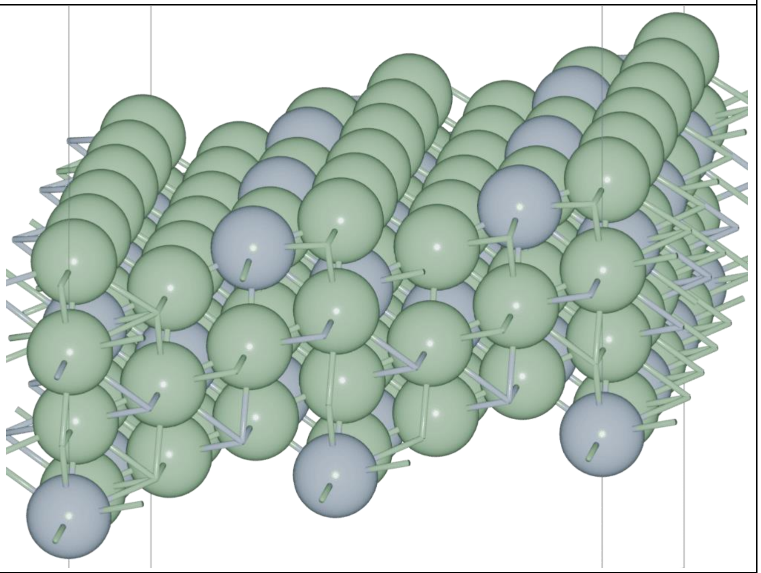

α'-$Ni_3Ga(211)_{Ga\text{-}step}$	4x4x1	2x3x1	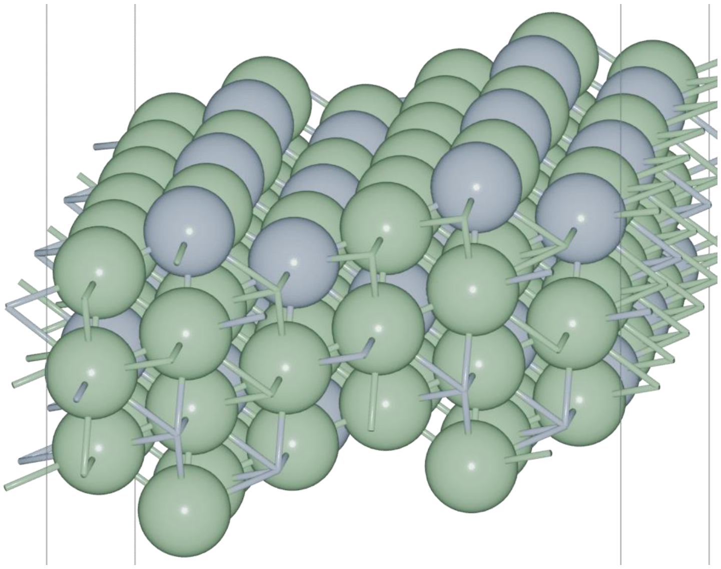
δ-$Ni_5Ga_3(221)$	8x8x1	1x1x1	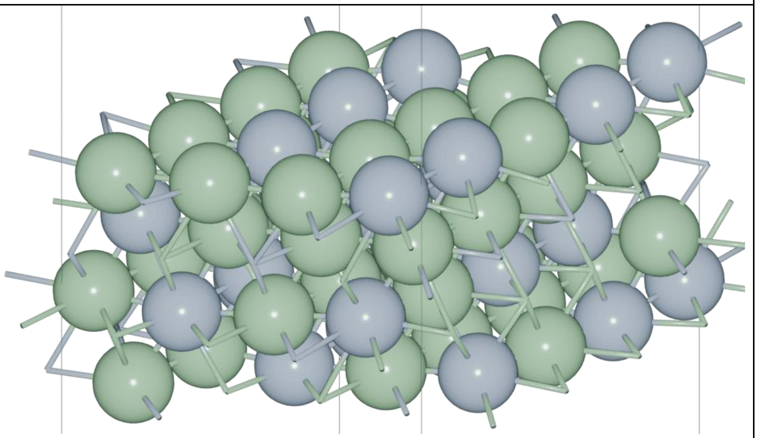
δ-$Ni_5Ga_3(211)_{Ni\text{-}step}$	8x8x1	1x1x1	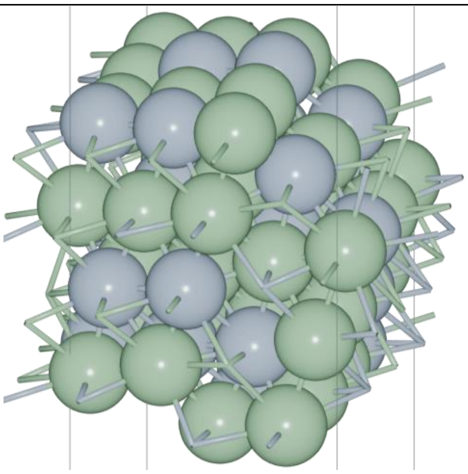

δ-$Ni_5Ga_3(211)_{Ga\text{-}step}$	8x8x1	1x1x1	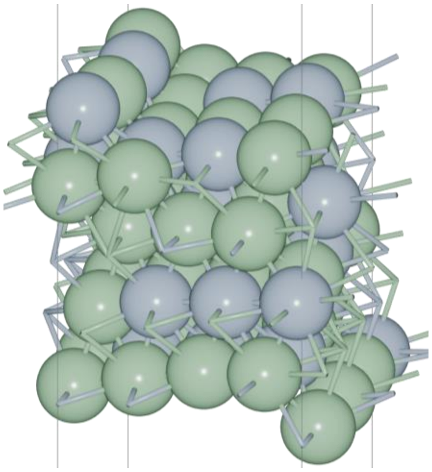
α'-$Ni_3Ga(111)$+GaO_x	4x4x1	3x3x1	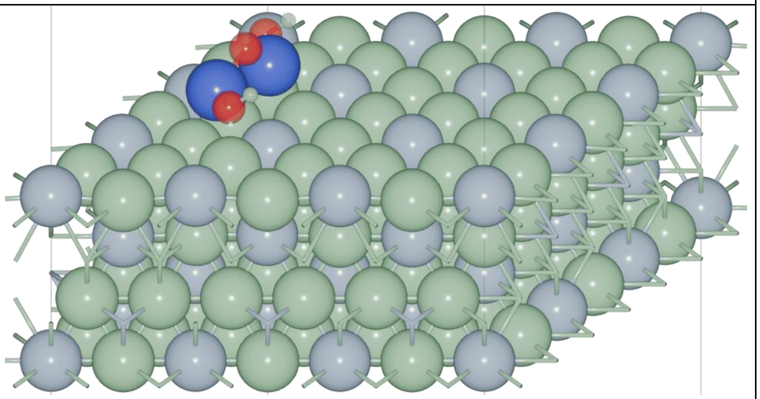
α'-$Ni_3Ga(211)_{Ni\text{-}step}$+$GaO_x$	4x4x1	2x3x1	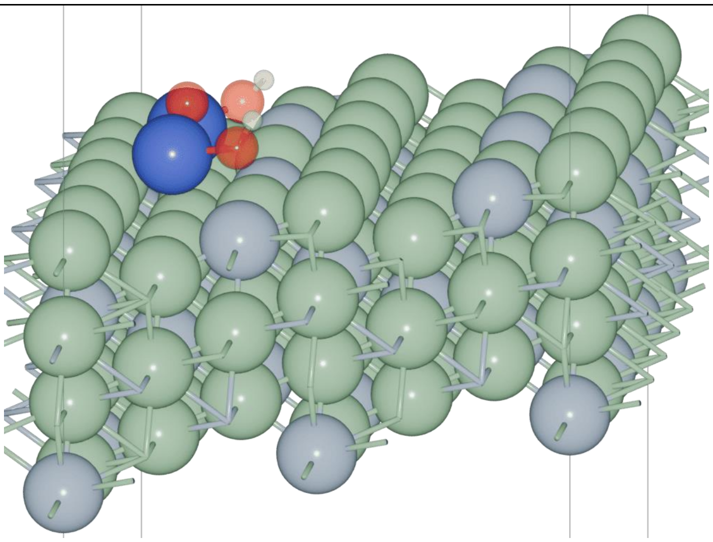

Surface energies and electronic properties

We considered surface energies to compare the stability of the generated surface terminations; here we compare the energy of the atoms present on a surface normalized by the exposed surface area to their energy in the respective bulk structure (Table S12). Two electronic properties were considered to compare the screened catalytic surfaces: the work function (φ) and the d-band center (ϵ_d).[11,12] The work function (φ) represents the minimum energy required to extract an electron from the Fermi level to the vacuum. Equation 8 defines the work function, W, where ϕ is the local electrostatic potential. ϕ is calculated as a function of the surface's perpendicular axis (z-axis). E_F is the Fermi level, E_{vac} is the vacuum energy, and -e is the charge of an electron. The d-band center, ϵ_d, is calculated from the partial density of states ($PDOS$) employing Equation 9, where $\rho(E)$ is the d-band density, and E is the d-band energy. The calculated work functions and d-band centers are reported in Table S13.

$$W = -e \cdot \phi - E_F = E_{vac} - E_F \tag{8}$$
$$\epsilon_d = \int_{-\infty}^{\infty} E \cdot \rho(E) dE \Big/ \int_{-\infty}^{\infty} \rho(E) dE \tag{9}$$

Furthermore, we calculated the Bader charge of the systems. We averaged the charge differences by atom type (i.e. Ni and Ga), using the electrons in the valence shell as a reference, to compare their electronic behavior as a function of stoichiometry, as shown in Table S14.

Table S 12. Calculated surface energies of the relaxed surfaces. [(a)] Reported values in the literature for Ni surfaces using the PBE functional.[13]

System	γ_{rel} [J m^{-2}]	γ_{rel} [J m^{-2}]
α-Ni(111)	1.992	1.946[(a)]
α-Ni(211)	2.243	2.279[(a)]
α'-Ni_3Ga(111)	1.584	-
α'-$Ni_3Ga(211)_{Ni\text{-}step}$	1.851	-
α'-$Ni_3Ga(211)_{Ga\text{-}step}$	1.869	-
δ-Ni_5Ga_3(221)	1.379	-
δ-$Ni_5Ga_3(211)_{Ni\text{-}step}$	1.575	-
δ-$Ni_5Ga_3(211)_{Ga\text{-}step}$	1.596	-

Table S 13. Calculated electronic properties. φ and ϵ_d denote the work function and the d-band center, respectively.

System	φ [eV]	ϵ_d [eV]
α-Ni(111)	5.37	-1.23
α-Ni(211)	5.08	-1.20
α'-Ni_3Ga(111)	4.99	-1.11
α'-$Ni_3Ga(211)_{Ni\text{-}step}$	4.77	-1.11
α'-$Ni_3Ga(211)_{Ga\text{-}step}$	4.77	-1.11
δ-Ni_5Ga_3(221)	5.03	-1.20
δ-$Ni_5Ga_3(211)_{Ni\text{-}step}$	4.84	-1.24
δ-$Ni_5Ga_3(211)_{Ga\text{-}step}$	4.81	-1.23
α'-Ni_3Ga(111)+GaO_x	5.06	-1.11
α'-$Ni_3Ga(211)_{Ni\text{-}step}$+$GaO_x$	4.92	-1.10

Table S 14. Average Bader charge for each type of atom, referenced against the electrons in the valence shell. [(b)]Average Bader charge for Ga atoms in the α'-Ni_3Ga surface . [(c)] Average Bader charge of the Ga atoms in the GaO_x cluster.

System	Ni [e-]	Ga [e-]
α-Ni(111)	0.000	-
α-Ni(211)	0.000	-
α'-Ni_3Ga(111)	-0.118	+0.353
α'-$Ni_3Ga(211)_{Ni\text{-}step}$	-0.114	+0.342
α'-$Ni_3Ga(211)_{Ga\text{-}step}$	-0.115	+0.344
δ-Ni_5Ga_3(221)	-0.178	+0.296
δ-$Ni_5Ga_3(211)_{Ni\text{-}step}$	-0.182	+0.303
δ-$Ni_5Ga_3(211)_{Ga\text{-}step}$	-0.182	+0.303
α'-Ni_3Ga(111) + GaO_x	-0.118	+0.356[(b)]/+1.090[(c)]
α'-$Ni_3Ga(211)_{Ni\text{-}step}$ + GaO_x	-0.113	+0.348[(b)]/+1.020[(c)]

Discussion of the calculated electronic properties

Bader charges

As expected, the Bader charge was zero e^- for pure Ni surfaces, indicating that Ni atoms share non-directional, metallic bonds. When Ni is in proximity to Ga, Ga transfers charge density to Ni, yielding a δ+ character for Ga and a δ- character for Ni, consistent with experimental Ni K edge XANES analysis. The averaged δ^+ values for Ga on α'-Ni_3Ga(111), α'-$Ni_3Ga(211)_{Ni\text{-}step}$ and α'-$Ni_3Ga(211)_{Ga\text{-}step}$ were +0.353, +0.342, and +0.344 e^-, respectively, while the δ^+ values for Ga on δ-Ni_5Ga_3(221), δ-$Ni_5Ga_3(211)_{Ni\text{-}step,}$ and δ-$Ni_5Ga_3(211)_{Ga\text{-}step}$ were slightly lower, viz. +0.296, +0.303, +0.303 e^-, respectively. These results indicate that the ratio Ni:Ga is the main factor influencing the Bader charge since no significant differences were observed between flat and stepped surfaces, including those with different step terminations. For α'-Ni_3Ga(111), α'-$Ni_3Ga(211)_{Ni\text{-}step}$ and α'-$Ni_3Ga(211)_{Ga\text{-}step}$, the δ^- values for Ni were -0.118, -0.114, and -0.115 e^-, respectively, while for δ-Ni_5Ga_3(221), δ-$Ni_5Ga_3(211)_{Ni\text{-}step,}$ and δ-$Ni_5Ga_3(211)_{Ga\text{-}step}$, the δ^- values for Ni were -0.178, -0.182, -0.182 e^-, respectively.

In addition, we performed a Bader charge analysis on the GaO_x/α'-Ni_3Ga system. Here, we first performed a When GaO_x is added to α'-Ni_3Ga there is a slight increase in the charge on the Ga atoms when compared to the pure α'-Ni_3Ga surfaces , viz. +0.356 e^- for α'-Ni_3Ga(111)+GaO_x and +0.348 e^- for α'-$Ni_3Ga(211)_{Ni\text{-}step}$+$GaO_x$, compared to +0.353 e^- and +0.342 e^- for the respective surfaces without GaO_x; the Bader charge on Ni remains nearly unchanged with the addition of GaO_x (-0.113 e^- for α'-Ni_3Ga(111)+GaO_x and -0.118 e^- for α'-$Ni_3Ga(211)_{Ni\text{-}step}$+$GaO_x$, compared to -0.114 e^- and -0.118 e^- for the surfaces without GaO_x). These results suggest that charge transfer occurs primarily from Ga atoms to the cluster, resulting in a subtle increase in the δ^+ character of the alloyed Ga atoms. Additionally, we conducted a Bader charge analysis on the GaO_x cluster (Figure S30). Each Ga center in the cluster has a charge of nearly one electron, indicating a +1 oxidation state for Ga. In comparison, each oxygen atom gains approximately one electron, suggesting Ga is partially oxidized by oxygen.

Next, further electronic properties i.e. the work function (φ) and the d-band center (ϵ_d), were compared across the proposed slab models. When comparing flat and stepped surfaces, φ was higher for flat surfaces ($\varphi_{flat} > \varphi_{stepped}$). This trend aligns with the surface energy results, as flat surfaces are thermodynamically more stable. The GaO_x/α'-Ni_3Ga interface shows an increased φ compared to the respective GaO_x-free surface. The position of the d-band center is often used as a descriptor for the catalytic activity of pure metals.[14] Thus, we calculated the ϵ_d of Ni for all of the modelled systems, as Ni is generally considered the active site in Ni-Ga based CO_2 hydrogenation catalysts.[15] We observe that α'-Ni_3Ga has the highest value at -1.11 eV and δ-Ni_5Ga_3 the lowest value at -1.24 eV, following the trend $\epsilon_{d\_Ni3Ga} > \epsilon_{d\_Ni} > \epsilon_{d\_Ni5Ga3}$. However, this descriptor does not account for variations in the local environment of Ni, making it unable to differentiate , for example, between Ni on flat or stepped terminations. Indeed, no significant differences were found when comparing ϵ_d of flat and stepped surfaces. In α'-Ni_3Ga, the presence of Ga shifts the ϵ_d of Ni closer to the Fermi level, while no such effect is observed for δ-Ni_5Ga_3. The shift of ϵ_d in α'-Ni_3Ga (compared to pure Ni) can be associated with Ga increasing the density of d-states near the Fermi level. When a GaO_x cluster is chemisorbed on the α'-Ni_3Ga(111) surface, there is a minimal effect on the ϵ_d of Ni. This is expected, as the electronic states of GaO_x are significantly lower in energy than those of Ni, resulting in a negligible effect on the surface's electronic structure.

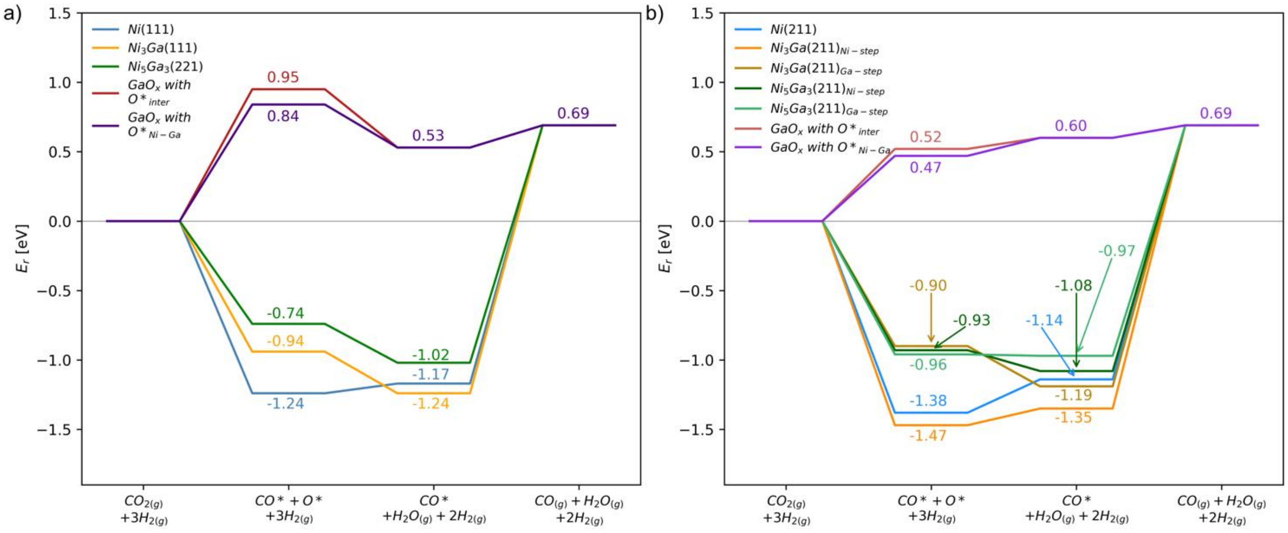

Figure S 31. Electronic reaction energies (E_r) for the reaction of CO_2 and $3H_2$ to form CO: (A) Flat surfaces and (B) stepped surfaces. GaO_x with O^*_{inter} indicates that O* interacts simultaneously with the GaO_x cluster and the α'-Ni_3Ga surface, i.e., the interface, while GaO_x with $O^*_{Ni\text{-}Ga}$ refers to a scenario where O* is bonded at the Ni-Ga surface.

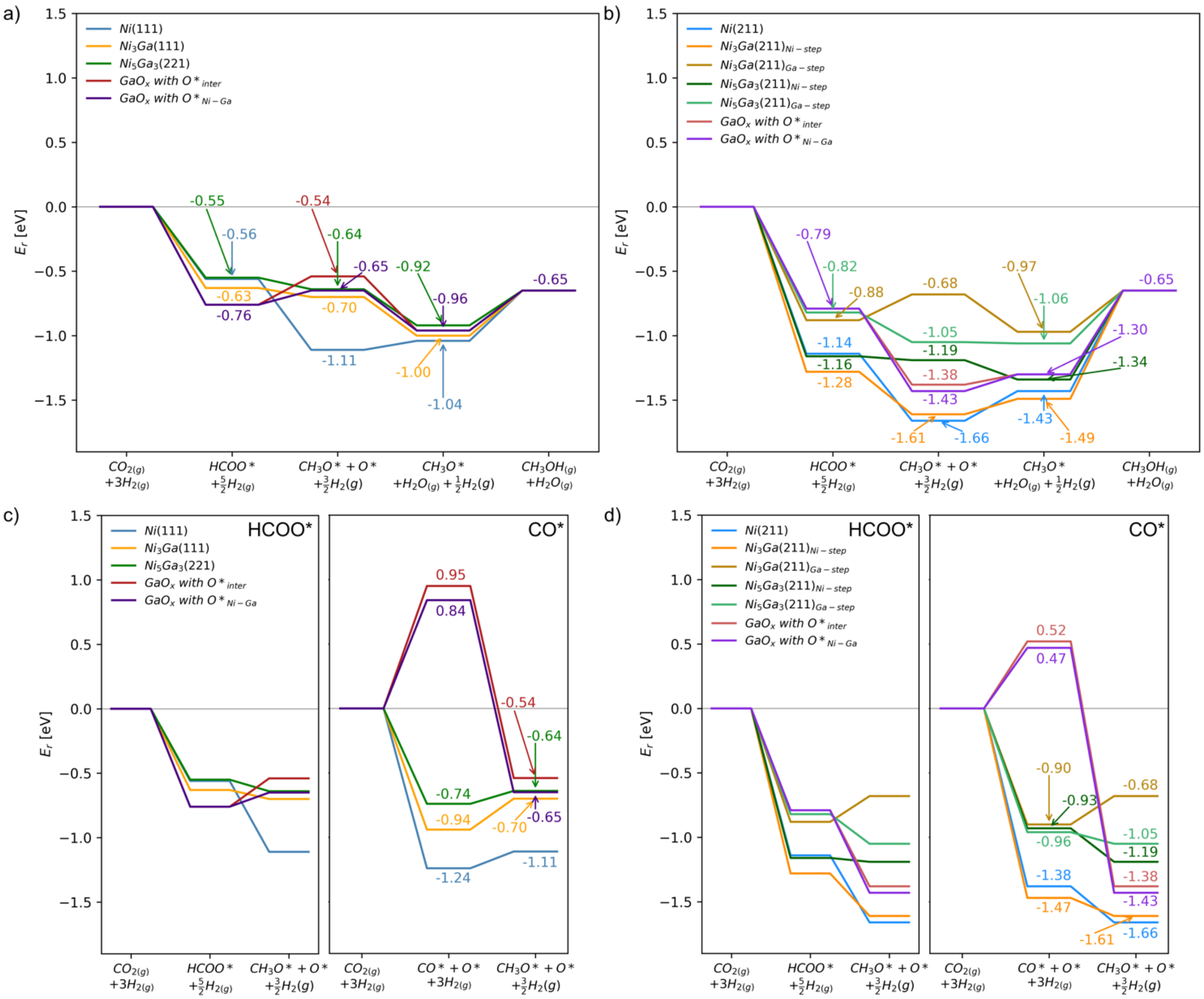

Figure S 32. Electronic reaction energies (E_r) for the reaction of CO_2 and $3H_2$ to form CH_3OH from HCOO*: (A) Flat surfaces and (B) stepped surfaces. (C, D) Comparison of CH_3O* and O* formation from HCOO* and CO*: (C) Flat surfaces and (D) stepped surfaces. GaO_x with O^*_{inter} indicates that O* interacts simultaneously with the GaO_x cluster and the α'-Ni_3Ga surface, i.e., the interface, while GaO_x with $O^*_{Ni\text{-}Ga}$ refers to a scenario where O* is bonded at the Ni-Ga surface.

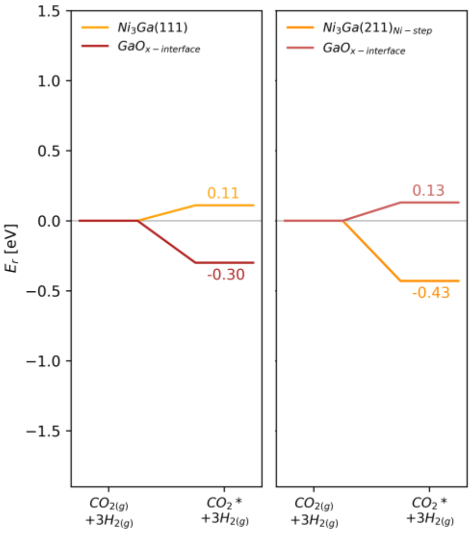

Figure S 33. Electronic adsorption energy of CO_2 on α'-$Ni_3Ga(111)$ (left) and α'-$Ni_3Ga(211)_{Ni\text{-}step}$ (right).

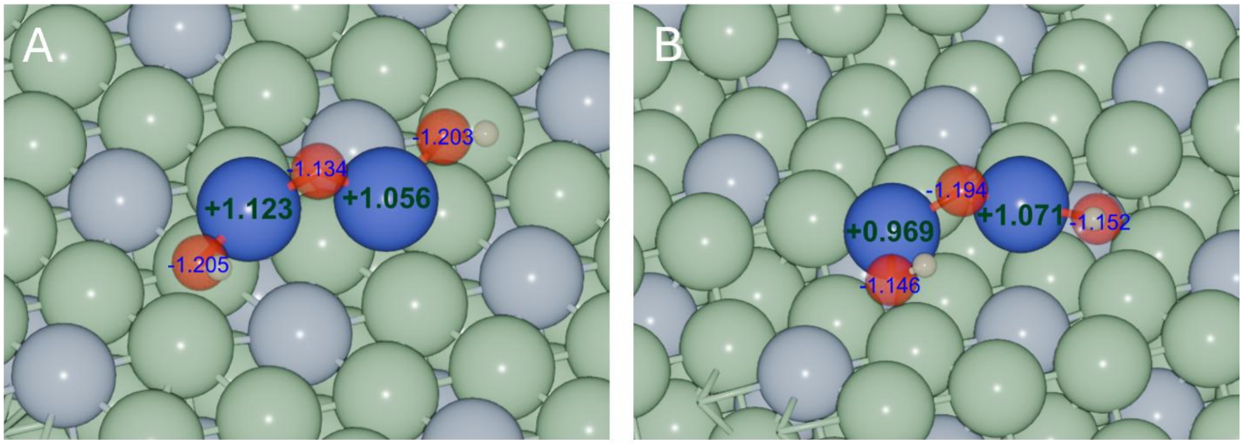

Figure S 34. Bader charge analysis: (A) α'-Ni_3Ga(111)+GaO_x and (B) α'-$Ni_3Ga(211)_{Ni\text{-}step}$+$GaO_x$. Negative values indicate a gain in charge, while positive values indicate depletion. Green atoms represent Ni and pale blue atoms Ga on the α'-Ni_3Ga surfaces while dark blue atoms represent Ga in the GaO_x cluster. Red atoms represent oxygen, and pale-yellow atoms represent hydrogen.

Table S 15. Electronic reaction energies of several intermediates using $CO_{2(g)}$+3$H_{2(g)}$ as the reference state without zero-point energy (ZPE) correction.

	E_r [eV]	E_r [eV]	E_r [eV]	$\Delta E_{r\text{-}HCOO^*}$ [eV]	E_r [eV]	E_r [eV]	$\Delta E_{r\text{-}H2COOH^*}$ [eV]
System	CO_2*	CO_2*+H*	HCOO*		HCOOH*+H*	H_2COOH*	
α'-Ni_3Ga(111)	+0.11	-0.21	-0.63	-0.42	-0.37	-0.40	-0.03
α'-$Ni_3Ga(211)_{Ni\text{-}step}$	-0.43	-0.53	-1.28	-0.75	-0.73	-0.81	-0.08
GaO_x/α'-Ni_3Ga(111)	-0.30	-0.64	-0.76	-0.12	-0.65	-0.61	+0.04
GaO_x/α'-$Ni_3Ga(211)_{Ni\text{-}step}$	+0.13	+0.28	-0.79	-1.07	-0.54	-0.83	-0.29

Table S 16. Barrier energies without Zero-point energy (ZPE) correction (in kJ/mol). The reaction pathways for the transition states H-COO‡ and H-HCOOH‡ can be found above.

	H-COO‡		H-HCOOH‡	
System	ΔE‡ [eV]	ΔE‡ [kJ/mol]	ΔE‡ [eV]	ΔE‡ [kJ/mol]
α'-Ni_3Ga(111)	0.72	69	1.05	101
α'-$Ni_3Ga(211)_{Ni\text{-}step}$	0.49	47	0.87	84

8. References

(1) Ravel, B.; Newville, M. ATHENA, ARTEMIS, HEPHAESTUS: Data Analysis for X-Ray Absorption Spectroscopy Using IFEFFIT. *J Synchrotron Rad* **2005**, *12* (4), 537–541. https://doi.org/10.1107/S0909049505012719.

(2) Zimmerli, N. K.; Rochlitz, L.; Checchia, S.; Müller, C. R.; Copéret, C.; Abdala, P. M. Structure and Role of a Ga-Promoter in Ni-Based Catalysts for the Selective Hydrogenation of CO_2 to Methanol. *JACS Au* **2024**, *4* (1), 237–252. https://doi.org/10.1021/jacsau.3c00677.

(3) Fehr, S. M.; Krossing, I. Spectroscopic Signatures of Pressurized Carbon Dioxide in Diffuse Reflectance Infrared Spectroscopy of Heterogeneous Catalysts. *ChemCatChem* **2020**, *12* (9), 2622–2629. https://doi.org/10.1002/cctc.201902038.

(4) Alrefae, M.; Es-sebbar, E.; Farooq, A. Absorption Cross-Section Measurements of Methane, Ethane, Ethylene and Methanol at High Temperatures. *Journal of Molecular Spectroscopy* **2014**, *303*, 8–14. https://doi.org/10.1016/j.jms.2014.06.007.

(5) Monti, D. M.; Cant, N. W.; Trimm, D. L.; Wainwright, M. S. Hydrogenolysis of Methyl Formate over Copper on Silica: I. Study of Surface Species by in Situ Infrared Spectroscopy. *J. Catal.* **1986**, *100* (1), 17–27. https://doi.org/10.1016/0021-9517(86)90067-9.

(6) Zhou, H.; Docherty, S. R.; Phongprueksathat, N.; Chen, Z.; Bukhtiyarov, A. V.; Prosvirin, I. P.; Safonova, O. V.; Urakawa, A.; Copéret, C.; Müller, C. R.; Fedorov, A. Combining Atomic Layer Deposition with Surface Organometallic Chemistry to Enhance Atomic-Scale Interactions and Improve the Activity and Selectivity of Cu–Zn/SiO_2 Catalysts for the Hydrogenation of CO_2 to Methanol. *JACS Au* **2023**, *3* (9), 2536–2549. https://doi.org/10.1021/jacsau.3c00319.

(7) Mihaylov, M.; Hadjiivanov, K.; Knözinger, H. Formation of $Ni(CO)_4$ during the Interaction between CO and Silica-Supported Nickel Catalyst: An FTIR Spectroscopic Study. *Catal. Lett.* **2001**, *76* (1), 59–63. https://doi.org/10.1023/A:1016786023456.

(8) Galhardo, T. S.; Braga, A. H.; Arpini, B. H.; Szanyi, J.; Gonçalves, R. V.; Zornio, B. F.; Miranda, C. R.; Rossi, L. M. Optimizing Active Sites for High CO Selectivity during CO_2 Hydrogenation over Supported Nickel Catalysts. *J. Am. Chem. Soc.* **2021**, *143* (11), 4268–4280. https://doi.org/10.1021/jacs.0c12689.

(9) Rasteiro, L. F.; Rossi, M. A. L. S.; Assaf, J. M.; Assaf, E. M. Low-Pressure Hydrogenation of CO_2 to Methanol over Ni-Ga Alloys Synthesized by a Surfactant-Assisted Co-Precipitation Method and a Proposed Mechanism by DRIFTS Analysis. *Catal. Today* **2021**, *381*, 261–271. https://doi.org/10.1016/j.cattod.2020.05.067.

(10) Agnelli, M.; Swaan, H. M.; Marquez-Alvarez, C.; Martin, G. A.; Mirodatos, C. CO Hydrogenation on a Nickel Catalyst: II. A Mechanistic Study by Transient Kinetics and Infrared Spectroscopy. *Journal of Catalysis* **1998**, *175* (1), 117–128. https://doi.org/10.1006/jcat.1998.1978.

(11) Hammer, B.; Nørskov, J. K. Electronic Factors Determining the Reactivity of Metal Surfaces. *Surface Science* **1995**, *343* (3), 211–220. https://doi.org/10.1016/0039-6028(96)80007-0.

(12) Sinfelt, J. H. Catalysis by Alloys and Bimetallic Clusters. *Acc. Chem. Res.* **1977**, *10* (1), 15–20. https://doi.org/10.1021/ar50109a003.

(13) Zhang, W.-B.; Chen, C.; Zhang, S.-Y. Equilibrium Crystal Shape of Ni from First Principles. *J. Phys. Chem. C* **2013**, *117* (41), 21274–21280. https://doi.org/10.1021/jp404569m.

(14) Vojvodic, A.; Nørskov, J. K.; Abild-Pedersen, F. Electronic Structure Effects in Transition Metal Surface Chemistry. *Top Catal* **2014**, *57* (1), 25–32. https://doi.org/10.1007/s11244-013-0159-2.

(15) Tang, Q.; Ji, W.; K. Russell, C.; Zhang, Y.; Fan, M.; Shen, Z. A New and Different Insight into the Promotion Mechanisms of Ga for the Hydrogenation of Carbon Dioxide to Methanol over a Ga-Doped Ni(211) Bimetallic Catalyst. *Nanoscale* **2019**, *11* (20), 9969–9979. https://doi.org/10.1039/C9NR01245A.
